# Positive Drought Feedbacks Increase Tree Mortality Risk in Dry Woodlands of the US Southwest

**DOI:** 10.1002/ece3.72667

**Published:** 2025-12-10

**Authors:** Kyle C. Rodman, Kaylie J. Wilkerson, Andreas P. Wion, David W. Huffman, Anita J. Antoninka, Mariola Barrera, Neil S. Cobb, Miranda D. Redmond

**Affiliations:** ^1^ Ecological Restoration Institute Northern Arizona University Flagstaff Arizona USA; ^2^ Southwest Office Forest Stewards Guild Santa Fe New Mexico USA; ^3^ School of Forestry Northern Arizona University Flagstaff Arizona USA; ^4^ Biodiversity Outreach Network Flagstaff Arizona USA; ^5^ Department of Environmental Science, Policy, and Management University of California Berkeley California USA

**Keywords:** arbuscular mycorrhizae, biotic interactions, crown dieback, *Juniperus monosperma*, *Pinus edulis*, tree mortality

## Abstract

Global changes in temperature and aridity are increasing the frequency of extreme drought events. Such changes can have pronounced impacts on dryland ecosystems, which exist at the margins of plant physiological tolerances. Pinyon–juniper (PJ) woodlands—a dryland vegetation type spanning 40 million ha in western North America—are a model system for the impacts of drought, where recurrent short‐interval drought events may trigger feedback mechanisms that influence future drought resistance. Leveraging a long‐term monitoring network in PJ woodlands of the United States (US) Southwest, we sought to understand how interactions between recurrent drought events influence tree mortality risk. We developed generalized linear mixed models to predict patterns of recent (i.e., 2014–2023) tree mortality based on biophysical variables, tree size, and prior drought‐driven changes (ca. 1998–2014) in forest conditions. We then used these models to quantify how mortality risk has shifted over time. Tree density and stand basal area declined substantially throughout our 1998–2023 monitoring period. Since 2014, tree mortality was more common and spatially extensive than new tree recruitment, and nearly half of the surviving trees experienced crown dieback. Tree size influenced biotic interactions and responses to environmental conditions, and soil organic matter and mycorrhizal fungi communities buffered individuals against drought. Shifts in woodland demographics (e.g., reduced stand densities, crown dieback) led to a 28.2% increase in mortality risk between 2014 and 2023 for trees that survived this period, a pattern that was consistent across species. Recent drought events have triggered widespread tree mortality and dieback in PJ woodlands of the US Southwest. These events also increase future tree mortality risk, overcoming system inertia created by local edaphic conditions and compensatory responses.

## Graphical abstract

Global changes in temperature and aridity can have pronounced impacts on dryland ecosystems, which exist at the margins of plant physiological tolerances. We investigated the drivers of recent tree mortality in pinyon–juniper (PJ) woodlands—a dryland vegetation type spanning 40 million ha in western North America. Shifts in woodland demographics (e.g., reduced stand densities, crown dieback) led to a 28.2% relative increase in mortality risk between 2014 and 2023, overcoming system inertia created by local edaphic conditions and compensatory responses.

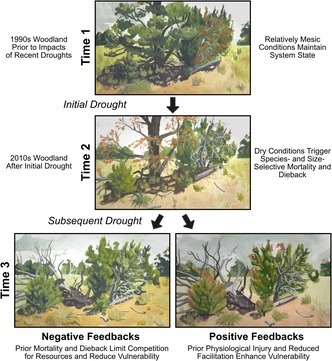

## 1 Introduction

Drought events are increasing in frequency and severity, with critical implications for social and ecological systems (Moss et al. 2024; Gazol et al. 2025). Warming temperatures associated with anthropogenic climate change are a primary driver of these increases, and they are creating novel combinations of heat and aridity that are unprecedented within the history of extant plant communities (Diffenbaugh and Scherer 2011; Williams et al. 2022). As a result, drought‐driven changes in forest and woodland ecosystems have been widely documented (McDowell et al. 2020; Hartmann et al. 2022). Drought can increase tree mortality rates (Senf et al. 2020; Hammond et al. 2022), modify community structure and composition (Bennett et al. 2015; Batllori et al. 2020), and reduce rates of carbon sequestration (Liu et al. 2023). Drought events may also interact with one another in complex ways to influence vegetation dynamics through “climate memory” or other legacy effects (Anderegg, Schwalm, et al. 2015; Anderegg et al. 2020; Kannenberg et al. 2020). How such events interact to shape forest and woodland ecosystem responses remains a key research need, which has implications for earth system models and human adaptation to future change (Müller and Bahn 2022).

Dryland ecosystems occupy nearly half of the terrestrial land surface and are critical to the well‐being of billions of people (Reynolds et al. 2007; Prăvălie 2016). Pinyon–juniper (PJ) woodlands, a dryland vegetation type spanning ca. 40 million ha in western North America, are composed of short‐statured trees (i.e., typically < 10 m in height) belonging to the genera *Pinus* and *Juniperus* (Romme et al. 2009; Redmond et al. 2023). PJ woodlands are notable for their cultural importance (Rhode and Madsen 1998; Whitehair et al. 2024) and support a range of taxa that includes birds, mammals, and arthropods (Balda and Masters 1980; Bombaci and Pejchar 2016; Uhey et al. 2020; Woolet et al. 2023). They are also a model system for the impact of drought. Widespread mortality and dieback events occurred in PJ woodlands during the mid‐1950s, 1990s, and early 2000s (Negrón and Wilson 2003; Breshears et al. 2005; Lloret and Kitzberger 2018), and the constituent species exhibit different physiological traits that influence drought resistance (Adams et al. 2017; McDowell et al. 2016). Pinyons (e.g., two‐needle pinyon; *P. edulis* ) are isohydric and have strong stomatal control, which reduces the potential for hydraulic failure but also limits photosynthetic capacity, whereas junipers (e.g., one‐seed juniper; *J. monosperma* ) are anisohydric and maintain open stomata and photosynthesis even under extreme drought (McDowell et al. 2008; *but see* Martinez‐Villalta and Garcia‐Forner 2017). Pinyons are also more susceptible to biotic agents (e.g., bark beetles, twig beetles) when compared with junipers (Flake and Weisberg 2019; Floyd et al. 2009). Mortality and crown dieback of both pinyon and juniper species have also occurred across the southwestern US throughout the 2010s and 2020s due to several intense drought years (Williams et al. 2022), which have exceeded important physiological thresholds and influenced population demographics (Kannenberg et al. 2021; Shriver et al. 2022; Wion et al. 2022).

**FIGURE 1:**
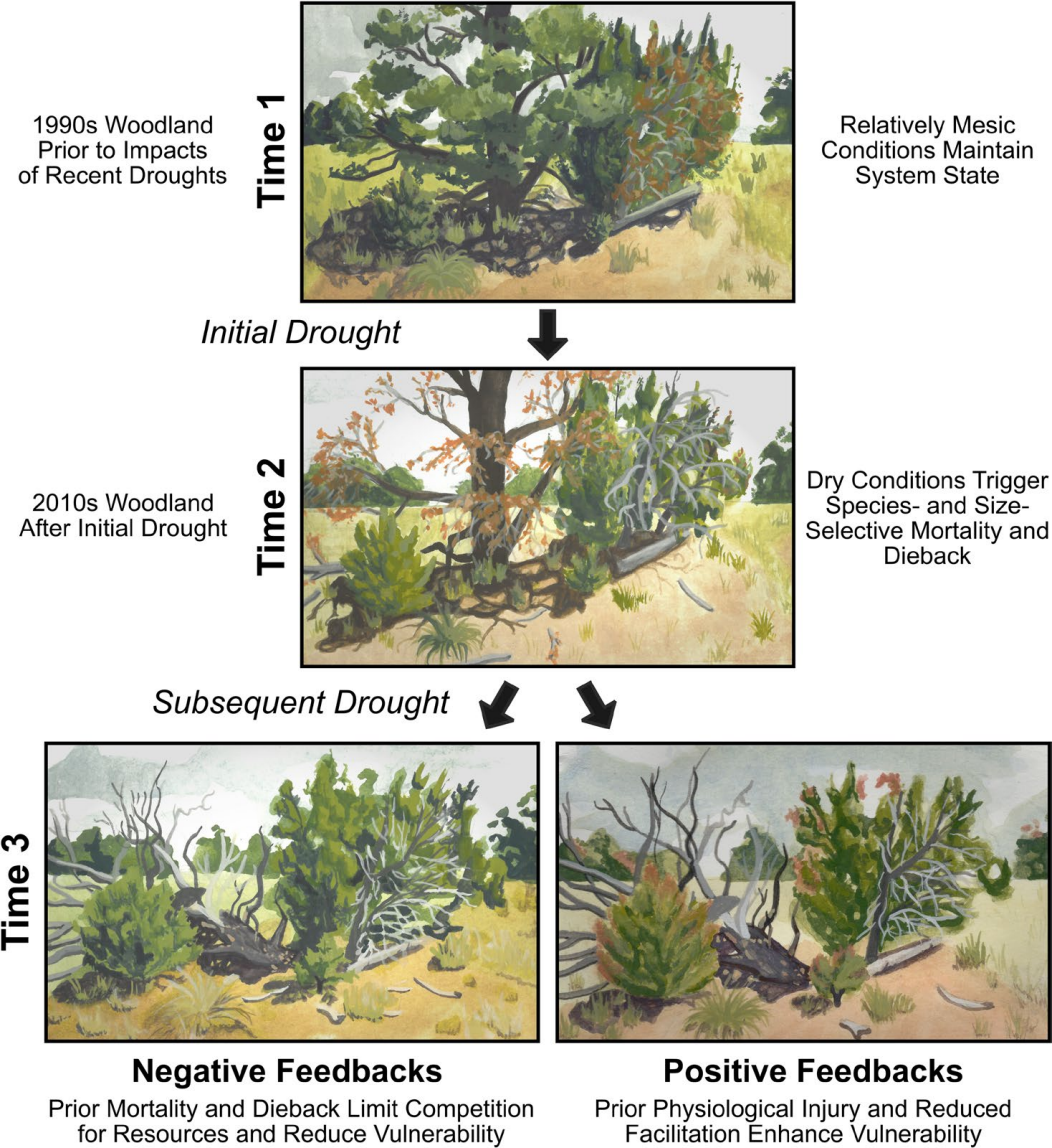
Dry woodlands may respond to recurrent, short‐interval drought events through multiple pathways. Top: A severe drought causes widespread tree mortality and non‐lethal crown dieback. Left pathway: Reduced competition for resources may moderate the effects of the subsequent drought (i.e., negative feedbacks). Right pathway: Instead, reduced facilitation and prior physiological injury could increase tree mortality risk (i.e., positive feedbacks). Original art by Mariola Barrera.

Initial tree mortality and dieback may influence PJ woodland ecosystems through pathways that either moderate or exacerbate the effects of subsequent drought (Figure 1). When initial disturbances alter the occurrence or severity of subsequent ones, they are considered “linked”, and such interactions can drive either positive or negative feedback dynamics (Simard et al. 2011; Burton et al. 2020; Dudney et al. 2025). Drought can trigger tree‐level physiological changes, drive selective mortality of less resistant individuals, and alter competitive or facilitative interactions (Clark et al. 2016; Trugman et al. 2021; Müller and Bahn 2022). Tree mortality in PJ systems differs strongly across sizes and species (Floyd et al. 2009; Negrón and Wilson 2003; Shriver et al. 2022), suggesting that initial droughts may remove the most sensitive individuals from a community. Likewise, denser stands are associated with a higher likelihood of mortality for many coniferous species in the US West, indicating that density reduction could enhance the drought tolerance of remaining individuals (Bradford and Bell 2017; Noel et al. 2023). However, existing research is mixed with respect to density‐dependent mortality in PJ woodlands (Bowker et al. 2012; Floyd et al. 2009; Greenwood and Weisberg 2008; Meddens et al. 2015; Schultz et al. 2023). Prior mortality or dieback may instead increase susceptibility to subsequent drought (Mueller et al. 2005) because of physiological impairment of surviving trees (Flake and Weisberg 2019) or declines in soil moisture availability (Morillas et al. 2017).

Fine‐scale processes such as plant–soil interactions and the availability of nurse structures (e.g., trees, shrubs, logs) influence tree survival through processes such as competition and facilitation (Peterman et al. 2013; Gehring et al. 2017; Urza et al. 2019; De Vries et al. 2023; Ferrenberg et al. 2025) that might be altered by drought events. For example, symbiotic soil organisms including mycorrhizal fungi can support tree resistance to drought (Pickles and Simard 2017). Pinyons associate with ectomycorrhizal (EM) fungi, whereas junipers and many shrubs have arbuscular mycorrhizal (AM) associations (Rousseau and Reid 1989; Haskins and Gehring 2004; Olsson et al. 2010). Tree mortality typically reduces EM colonization and alters soil microbial communities, though impacts to AM communities are less clear (Swaty et al. 2004; Hopkins et al. 2018; Liao et al. 2025). Drought also has the potential to influence soil chemistry. In dryland systems, woody plants can create islands of fertility which have elevated levels of soil nutrients that facilitate the establishment of smaller plants (Ferrenberg et al. 2025). However, soil nutrients such as carbon and nitrogen often have short residence times (Neff et al. 2009) and may decline following tree mortality as documented in other systems (Xiong et al. 2011). Mortality of larger trees can also reduce nurse structure availability, which may influence microclimatic conditions and the survival of tree seedlings (Floyd et al. 2015; Redmond et al. 2018; Urza et al. 2019). Thus, short‐interval drought events could interact through mechanisms related to stand structure, plant–soil interactions, and other biological legacies (Figure 1), which may be further mediated by abiotic conditions (e.g., Bertness and Callaway 1994). Disentangling these effects requires long‐term data spanning broad environmental gradients.

**FIGURE 2:**
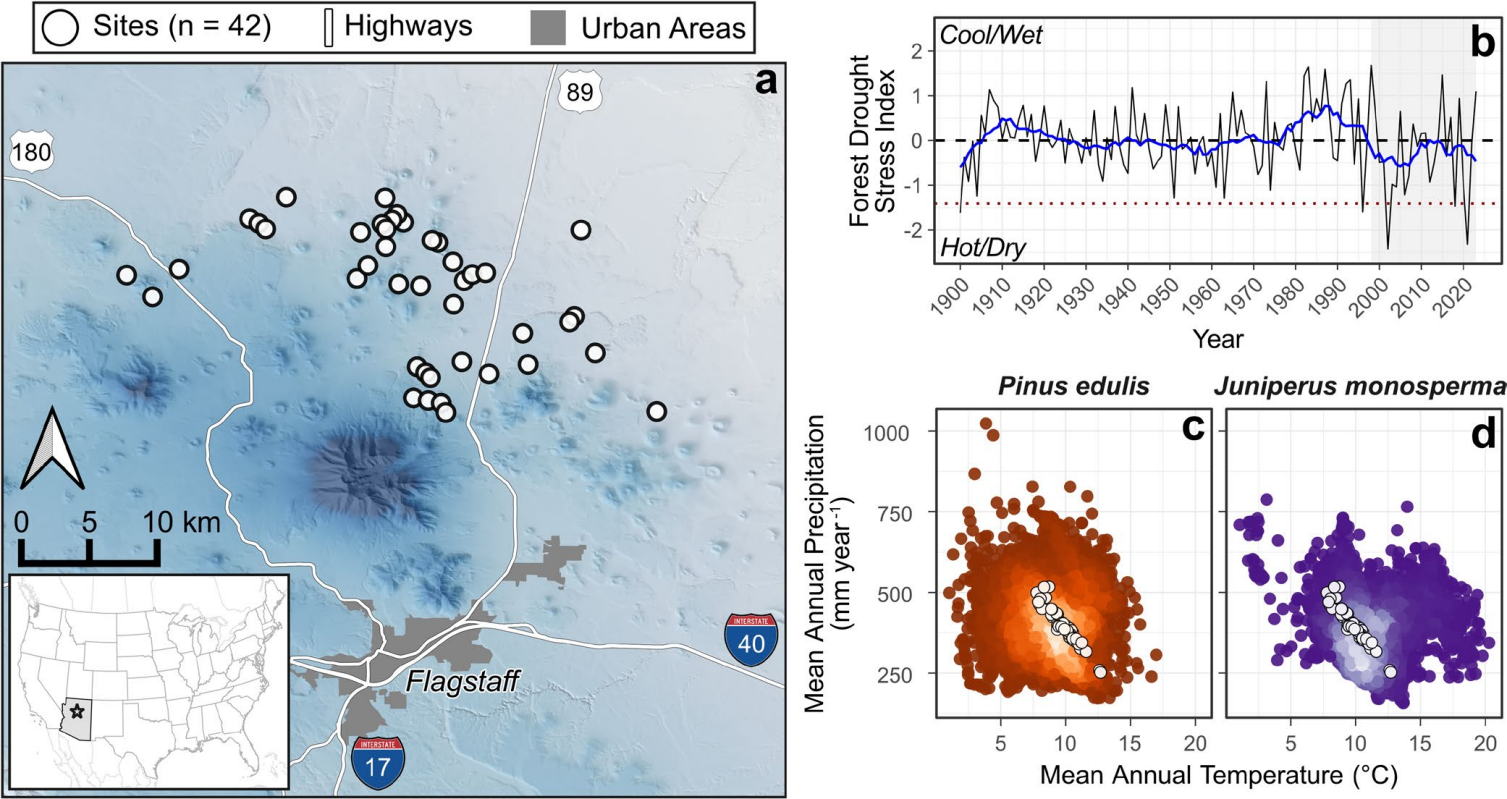
Description of the study area in pinyon ( *Pinus edulis* ) and juniper ( *Juniperus monosperma* ) woodlands of northern Arizona, US. Panel (a) shows the locations of 42 permanent monitoring sites, with background shading representing elevation from ca. 1350 m (light blue) to 3850 m (dark blue). In panel (b), the solid black line gives annual values of the Forest Drought‐Stress Index (FDSI) (Williams et al. 2013) within the study area, and the blue line shows a 10‐year running mean. The red dashed line in (b) indicates a drought‐stress threshold associated with forest decline. Colored background points in (c, d) show typical climatic conditions (i.e., 1991–2020 climate normals) in 10,000 random locations across each species' range (Wilson et al. 2013), with lighter colors representing greater point density. In (c, d) black and white points give the average climate of individual monitoring sites shown in (a).

This study leveraged a unique, long‐term (ca. 25 years old with remeasurements roughly every 5 years) monitoring network in PJ woodlands of the US Southwest to assess how interactions between recent recurrent drought events influenced tree mortality risk. This network spans much of the climatic distribution of two‐needle pinyon and one‐seed juniper (Figure 2) and covers a broad range of stand structural stages and edaphic conditions. Our monitoring period also includes the driest period (i.e., 2000–2021) that has occurred throughout the US Southwest in at least 1200 years (Williams et al. 2022), providing an analog for future climate impacts in this region. In this study, we focused on the following questions: (Q1) How have PJ woodlands changed throughout a 25‐year megadrought? (Q2) What biophysical factors were most strongly related to recent tree survival, and are they suggestive of positive or negative feedbacks? (Q3) How have recent demographic shifts in PJ woodlands altered near‐term mortality risk? Answering these questions helps to improve our understanding of how biotic interactions, environmental gradients, and legacies of past disturbance shape the future of woody plant communities in dryland ecosystems.

## 2 Materials and Methods

### 2.1 Study Area

Our study area, centered at 35.5° N and 111.6° W, covers ca. 1500 km^2^ of PJ woodlands in northern Arizona, US (Figure 2a). The average climate of this area is continental and semi‐arid, with mean annual temperatures (30‐year normals from 1991 to 2020) of 9.9°C and mean annual precipitation of 396 mm year ^−1^. Average minimum January temperatures are −7.3°C, July maximum temperatures are 29.8°C, and diurnal fluctuations typically exceed 16°C. There is a notable dry period in June (1.6% of annual precipitation) and wet period from July to September (42.6% of annual precipitation), with more consistent precipitation across other months (ranging from 4.5% to 9.3% of annual precipitation) (PRISM Climate Group, Oregon State University 2023). Soils are derived from basalt, basaltic cinder, or mixed igneous parent materials, and texture and organic matter (OM) content vary considerably across sites (Redmond et al. 2015; Chaney et al. 2016). Humans have inhabited and used this landscape for at least 13,000 years before present (Downum 1993); the surrounding area is of cultural significance to at least 13 modern Indigenous nations (Native Land Map API 2021; Wupatki National Monument‐People 2025). Forced displacement and genocide of Indigenous peoples occurred throughout the late 1800s and 1900s. Contemporary land use includes cattle grazing, firewood harvesting, and wind energy production.

Two‐needle pinyon and one‐seed juniper are the dominant tree species in our study area, together representing 92.8% of total tree density and 87.1% of total basal area (BA). Average temperature and precipitation of the research sites include those found in > 70% and > 85%, respectively, of the distribution of these species across the US West (PRISM Climate Group, Oregon State University 2023; Wilson et al. 2013; Figure 2c,d). PJ woodlands incorporate a diverse array of structural conditions; our sites range from open savanna systems at lower elevations to dense woodlands intermixed with more mesic tree species at high elevations. Ponderosa pine ( *P. ponderosa* ) and alligator juniper ( *J. deppeana* ) are present on wetter sites, in limited numbers. Utah juniper ( *J. osteosperma* ) can also be found in nearby areas, and all three juniper species (*J. deppeana, J. monosperma, J. osteosperma*) are known to hybridize in the Southwest. Typical shrubs include broom snakeweed ( *Gutierrezia sarothrae* ), rubber rabbitbrush ( *Ericameria nauseosa* ), and cliffrose ( *Purshia stansburyana* ). Herbaceous plant communities include forbs such as King's lupine ( *Lupinus kingii* ), western tansymustard ( *Descurainia pinnata* ), and woolly plantain ( *Plantago patagonica* ), as well as the grasses blue grama ( *Bouteloua gracilis* ), mutton bluegrass ( *Poa fendleriana* ), and mountain muhly ( *Muhlenbergia montana* ).

### 2.2 Field Data Collection and Laboratory Methods

From 1998 to 2001, we established 42 permanent monitoring sites that span the elevational range (1680 to 2300 m) and topographic conditions of PJ woodlands in our study area (further described in Redmond et al. 2015). At each site, we installed a 10 × 200‐m belt transect, divided it into twenty 10 × 10‐m fixed‐area plots (100 m^2^), and sampled alternating plots along each transect (i.e., 10 plots total per transect). At a subset of sites (*n* = 7) with lower tree densities, we installed longer transects (up to 300 m), resulting in up to 15 plots per transect. During site establishment, we stem‐mapped all seedlings and trees within each plot boundary and recorded the diameter at root collar (DRC), height, species, and status (live or dead) of each individual. We permanently marked plot corners using rebar and tagged a subset of trees on each plot to facilitate resampling efforts in 2004, 2006–2007, 2010, 2014, 2018, and 2022–2023. In each remeasurement, we determined the status of all trees and recorded the DRC, height, and location of any newly established seedlings. For our analyses, we assumed that individual DRC did not vary throughout the study period, because trees are slow growing in PJ woodlands, and measurement error often exceeds the effects of growth (Appendix S2). We remeasured a portion of the sites in 2010 (*n* = 28) and 2018 (*n* = 18) but visited all sites in the remaining sampling periods. Across all survey periods, we monitored a total of 5086 individuals (adults and juveniles) on 416 field plots. We also quantified the amount of living crown on each tree, as well as plot‐scale ground cover in 2014 and 2022–2023 remeasurements. To characterize ground cover, we used the line‐point intercept method with a 14‐m transect diagonally across each plot and points located at 0.5‐m increments, as described in Redmond et al. (2015).

We quantified edaphic conditions at our sites using two sampling efforts in 2014 and 2023. In 2014, we collected soil samples (2.5‐cm diameter × 10‐cm depth) at each site to describe surface soil texture and OM content. These soil samples were processed at the Colorado State University Soil Laboratory in Fort Collins, Colorado. Soil texture was characterized in the laboratory using the hydrometer method, and OM was quantified using hydrogen peroxide and hydrochloric acid (Gee and Or 2002). We also used a targeted sampling effort in 2023 to characterize AM communities (Haskins and Gehring 2004) and their relationship with the survival of small juniper trees. To do so, we first identified plots (*n* = 87) with large (i.e., > 25 cm DRC), live junipers that were present in the 2022 measurements. Large, live trees help facilitate the effective sampling of mycorrhizal communities. From this subset, we then identified plots (*n* = 34) that contained live juniper seedlings (i.e., < 5‐cm DRC) in 2014 that were remeasured for survival in 2022. We completed sampling and processing for 26 of these plots as follows. We collected soil samples (11.4‐cm diameter × 10‐cm depth) below the north dripline of the large, live juniper tree closest to plot center. Following methods described in Johnson et al. (1999), we sieved, washed, and stained root materials to accentuate AM structures. We then visually assessed the presence of AM hyphae, vesicles, and arbuscules at 150 root intersection points using 200× magnification to calculate Total AM colonization following McGonigle et al. (1990) (Appendix S1).

### 2.3 Analytical Methods

To answer *Q1*, focusing on changes in woodland conditions over time, we used records of tree status, species, and DRC to describe shifts in density, BA, and relative dominance from 1998 to 2023. We calculated relative dominance by summing the percentages of tree density and BA belonging to a given species; these values range from 0 (species absent) to 200 (species comprises 100% of total density and BA) (Curtis and McIntosh 1951). We calculated these metrics within each site and remeasurement to describe localized patterns of change. We then summarized changes in structure and composition across the study area using the mean values of all sites within the full remeasurement periods (i.e., 1998–2001, 2004, 2007, 2014, 2022–2023). Eight sites burned in wildfires that occurred in 2021 or 2022, and two experienced large‐scale tree removal from mechanical treatments in the late 2010s. We included these sites in graphical descriptions of local change until the year in which fire or management occurred but excluded them from descriptions of average trends across the study area, to ensure these comparisons tracked the same sites over time. These sites were excluded from all quantitative analyses. Finally, we characterized crown dieback and mortality of two‐needle pinyon and one‐seed juniper between 2014 and 2023 using Live Crown class (Table 1) and status, respectively, of all trees that were recorded in both measurements. We determined if a tree experienced non‐lethal dieback in this period if Live Crown declined between 2014 and 2023.

**TABLE 1:** Summary of variables considered for inclusion in generalized linear mixed models of tree survival from 2014 to 2023. Shortened names of each variable are given in parentheses.

Variable name	Method of calculation (units)	Rationale
Average climatic water deficit (average CWD)	Unmet evaporative demand of the atmosphere (average mm year^−1^ for the 1991–2020 period). CWD was calculated using temperature, precipitation, soils data, and topography in a modified Thornthwaite water balance model at a monthly timestep. Plot‐scale	Tree responses to drought are likely to vary across environmental gradients. CWD is an indicator of moisture stress experienced by plants
Diameter at root collar (DRC)	Basal diameter of a tree near ground level (cm), recorded during field plot installation or 2014 remeasurements. Tree‐scale	Tree size can influence biotic interactions and environmental sensitivity
Live basal area (Live BA)	Cross‐sectional area (m^2^ ha^−1^) of all live trees present in a plot in 2014. Plot‐scale	Neighborhood density can promote facilitative or competitive effects in woodlands
Live crown	Percentage of tree crown with live foliage, recorded in the field in 2014. Subdivided into four categories: < 15%, 16%–50%, 51%–90%, > 90%. Tree‐scale	Leaf area supports photosynthesis and influences physiological performance
Proportion BA loss	The proportion of live BA present in a plot during study initiation (i.e., 1998–2001) that died by 2014. Plot‐scale	Preceding tree mortality may increase resource availability and competitive interactions or reduce facilitation of small trees
Soil organic matter (OM)	Percentage of the top 10 cm of the soil horizon composed of organic matter, based on field‐derived soil samples collected in 2014. Site‐scale	OM can influence moisture retention and nutrient availability. OM may decline following tree dieoff
Total arbuscular mycorrhizal colonization (total AM)	The percentage of root tips with evidence of AM colonization based on soil samples collected in 2023. Plot‐scale	AM form mutualistic relationships with juniper and facilitate moisture and nutrient acquisition. Dieoff or dieback may alter soil communities
Wood cover	The proportion of the ground surface covered in woody debris (i.e., > 1‐cm diameter) during 2014 measurements. Recorded in the field using the line‐point intercept method. Plot‐scale	Wood cover can enhance soil moisture and provide nurse effects for smaller trees. Wood cover may increase following tree mortality

To answer *Q2*, focusing on how recent survival was related to biophysical factors, ontogeny, and drought‐caused changes of the early 2000s, we developed three generalized linear mixed models (GLMMs) to predict tree survival between 2014 and 2023 using variables described in Table 1. Further descriptions of these variables and methods of calculation are available in Appendix S2. We treated our response as a binary, tree‐scale value (i.e., survival or mortality), using a binomial error structure and complementary log–log link (i.e., Gompit regression; Salas‐Eljatib and Weiskittel 2020). To account for spatial dependence (i.e., multiple plots within each site and multiple trees within each plot), we used a nested random intercept term of plot within site. For the first two models, we analyzed survival of two‐needle pinyon (*n* = 370; Model #1) and one‐seed juniper (*n* = 757; Model #2) that met the following criteria: (1) individuals that were present in both 2014 and 2022–2023 remeasurements and (2) > 5 cm in height or > 0.5‐cm DRC in 2014. We excluded the smallest individuals (primarily first‐year germinants) because they were often missing in remeasurements and had very high mortality rates. In each model, we tested fixed‐effect terms of Average CWD, DRC, Live BA, Live Crown, Proportion BA Loss, Soil OM, and Wood Cover (Table 1). Because trees may have different environmental responses across life stages, we included two‐way interaction terms of DRC × Live BA and DRC × Average CWD. Likewise, because competitive release might be more apparent on plots with both high initial BA and high mortality, we included a two‐way interaction term of Live BA × Proportion BA Loss. We excluded other two‐ and three‐way interaction terms because they led to model convergence failure or overfitting.

In Model #3, we focused on the survival of small juniper (i.e., individuals < 5‐cm DRC in 2014, but excluding first‐year germinants) as it related to tree size and AM colonization. We focused specifically on small junipers for this analysis because pinyons have different mutualistic soil communities that are comparatively well studied (e.g., Gehring and Whitham 1994; Gehring et al. 2017), and because advanced regeneration of juniper may help to support future woodland persistence in the study area (Redmond et al. 2015). We acknowledge that this analysis, limited to plots that contained large, live trees, inherently focuses on less drought‐impacted sites. In this model, we tested for fixed effects of DRC and Total AM colonization, as well as a two‐way interaction between them. To help interpret the results of other models and infer how soil communities might respond to future tree mortality, we also developed three univariate GLMMs to describe how Total AM related to (1) Live BA of juniper in 2014, (2) Soil OM in 2014, and (3) Average CWD from 1991 to 2020 (Appendix S1).

We developed full versions of each model (#1–#3) using each of the potential covariates and interactions described above. First, we scaled and centered all continuous, numeric variables following Gelman (2008) to compare effect sizes and improve model stability. We then used an all‐subsets model selection procedure to identify the set of covariates and two‐way interactions that minimized the sample‐size‐corrected Akaike Information Criterion (AICc) (hereafter, “top model”). Though we primarily focus on the top model, we also briefly interpret the results of competing models to assess the directionality and consistency of covariate effects (Tables S4.2, S4.5 and S4.8). Top models met necessary assumptions (e.g., spatial independence, normal dispersion, lack of collinearity) based on standard residual diagnostics. We described model goodness of fit using pseudo *R* ^2^ following Nakagawa et al. (2017) as well as the area under the curve (AUC) statistic for fixed effects (i.e., *R* ^2^_m_, AUC_m_) and the full model (i.e., *R* ^2^_c_, AUC_c_). We completed all analyses in R software v. 4.4.0 (R Core Team 2024) using the *glmmTMB* (Brooks et al. 2017), *MuMIn* (Bartón 2018), *pROC* (Robin et al. 2011), *DHARMa* (Hartig 2018), *ncf* (Bjornstad 2019), and *performance* (Lüdecke et al. 2021) packages.

### 2.4 Predicting Mortality Risk Due to Demographic Shifts

To answer *Q3*, focusing on how demographic shifts have influenced mortality risk in PJ woodlands, we used Models #1 and #2 (described in Section 2.3.) to predict the survival probability of pinyon and juniper trees present in 2014 and 2022–2023 remeasurements. We predicted tree‐scale values, then (1) calculated the mean survival probability by year and (2) estimated upper and lower bounds of predicted mean values by adding and subtracting, respectively, one standard error of the mean as follows:

$$\mu_{\mathrm{yr}}=\frac{\sum_{i=1}^{n}p{\left(\text{Survival}\right)}_{i,\mathrm{yr}}}{n_{\mathrm{yr}}}$$

$$\sigma_{\mathrm{yr}}=\sqrt{\frac{\sum_{i=1}^{n_{\mathrm{yr}}}{\left(p{\left(\text{Survival}\right)}_{i,\mathrm{yr}}-\mu_{\mathrm{yr}}\right)}^{2}}{n_{\mathrm{yr}}}}$$

$$\text{Survival}{\left(\text{upper},\text{lower}\right)}_{\mathrm{yr}}=\mu_{\mathrm{yr}}\pm \frac{\sigma_{\mathrm{yr}}}{\sqrt{n_{\mathrm{yr}}}}$$

where *p*(Survival)_*i,yr*_ represents the model‐predicted survival probability of tree *i* in year *yr* and *n*_*yr*_ represents the total number of individuals in the population in year *yr*. Finally, we converted interval‐specific survival probabilities to annualized mortality rates using the following equation:

$$\text{Mortality}=\left\{\left(1-\left(\frac{\sum_{i=1}^{n}p{\left(\text{Survival}\right)}_{i}}{n}\right)\right)/8.5\right\}\times 100$$

Here, 8.5 represents the average length of the sampling period in years (i.e., 2014 to 2022–2023). We used a constant of 8.5 because random effect structures in the GLMMs accounted for site‐level differences in sampling intervals (i.e., 8 or 9 years), and roughly half of all trees were surveyed each year (2022 or 2023).

## 3 Results

### 3.1 Changes in Woodland Condition Throughout a 25‐Year Megadrought

**FIGURE 3:**
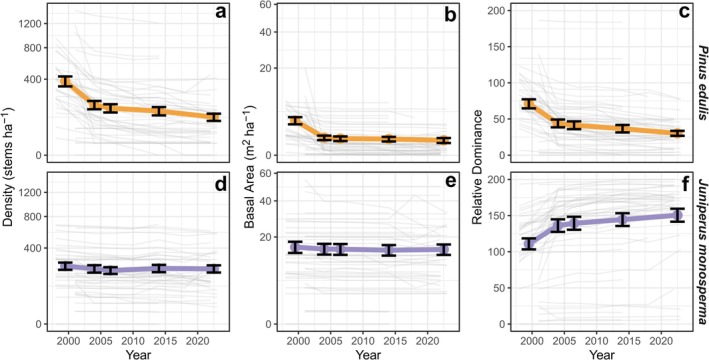
Changes in tree density (a, d; all stems taller than 5 cm or > 0.5 cm in diameter), basal area (b, e), and relative species dominance (c, f) in pinyon ( *Pinus edulis* ) and juniper ( *Juniperus monosperma* ) woodlands in northern Arizona, USA from 1998 to 2023. In each panel, gray lines show trends within individual sites, and thicker, colored lines show trends across all sites. Points give the mean value of each variable within remeasurement intervals (1998–2001, 2004, 2006–2007, 2014, and 2022–2023) when the majority of sites were surveyed, and error bars show the mean ±1 standard error.

The structure and composition of PJ woodlands in our study area shifted between 1998 and 2023 (Figure 3, Table S4.1). Live tree density and BA of two‐needle pinyon declined by 74% and 81.6%, respectively (Figure 3a,b). At the same time, live one‐seed juniper densities declined by 9.2% and BA declined by 6.1% (Figure 3d,e). Overall, these changes led to a 58.2% reduction in the relative dominance of pinyon and a 36.2% increase in the dominance of juniper (Figure 3c,f). Pinyon shifted towards intermediate tree sizes due to selective mortality of both the largest and smallest individuals, whereas juniper size structure remained relatively consistent over time (Figure S4.1).

**FIGURE 4:**
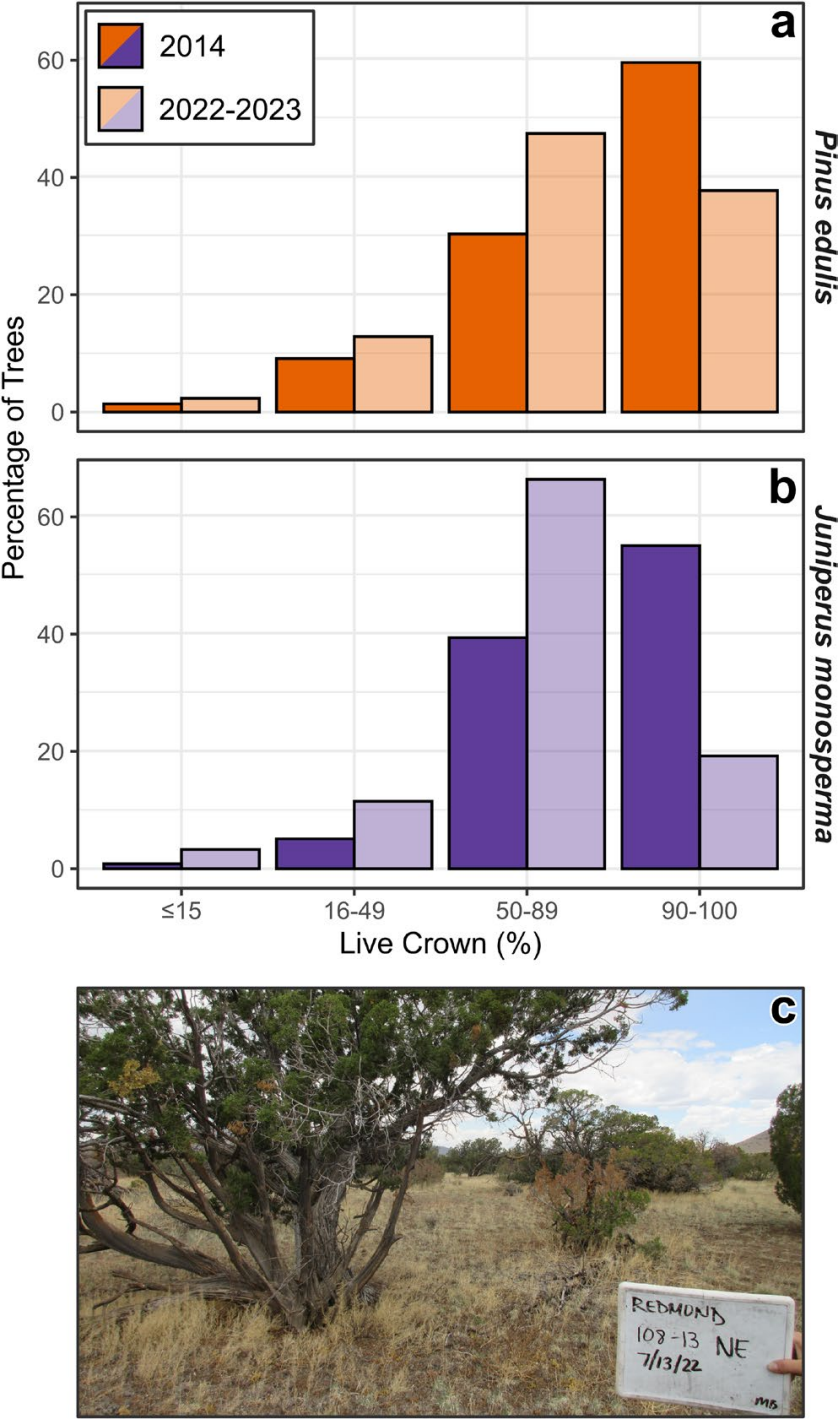
Non‐lethal dieback occurred throughout pinyon ( *Pinus edulis* ) and juniper ( *Juniperus monosperma* ) woodlands of northern Arizona, USA, between 2014 and 2023. Bar heights in (a, b) show the percentage of live trees of each species with different amounts of living foliage (*x*‐axis) during 2014 and 2022–2023 field surveys. A photo of recent crown dieback (taken in 2022) of several junipers is shown in (c).

Substantial changes also occurred from 2014 to 2023, the most recent remeasurement period. Overall, 18.4% of trees present in 2014 died by 2023 (an annualized mortality rate of 2.2% year^−1^) and mortality occurred in 41.8% of monitoring plots. In comparison, 34.0% of live trees initially present at these sites died between 1998 and 2004. Between 2014 and 2023, mortality was nearly twice as common for pinyons (26.3% of trees) as for junipers (14.5% of trees). New establishment in this period (*n* = 210 seedlings) represented 16.6% of the 2014 population but was present in just 13.4% of the monitoring plots. Non‐lethal crown dieback was present in 47.9% of surviving trees (Figure 4). This pattern was relatively consistent across species, with 46.3% of surviving pinyon and 48.6% of surviving juniper showing evidence of dieback (Figure 4a,b).

### 3.2 Predictions of Tree Survival and Interactions Between Short‐Interval Droughts

**FIGURE 5:**
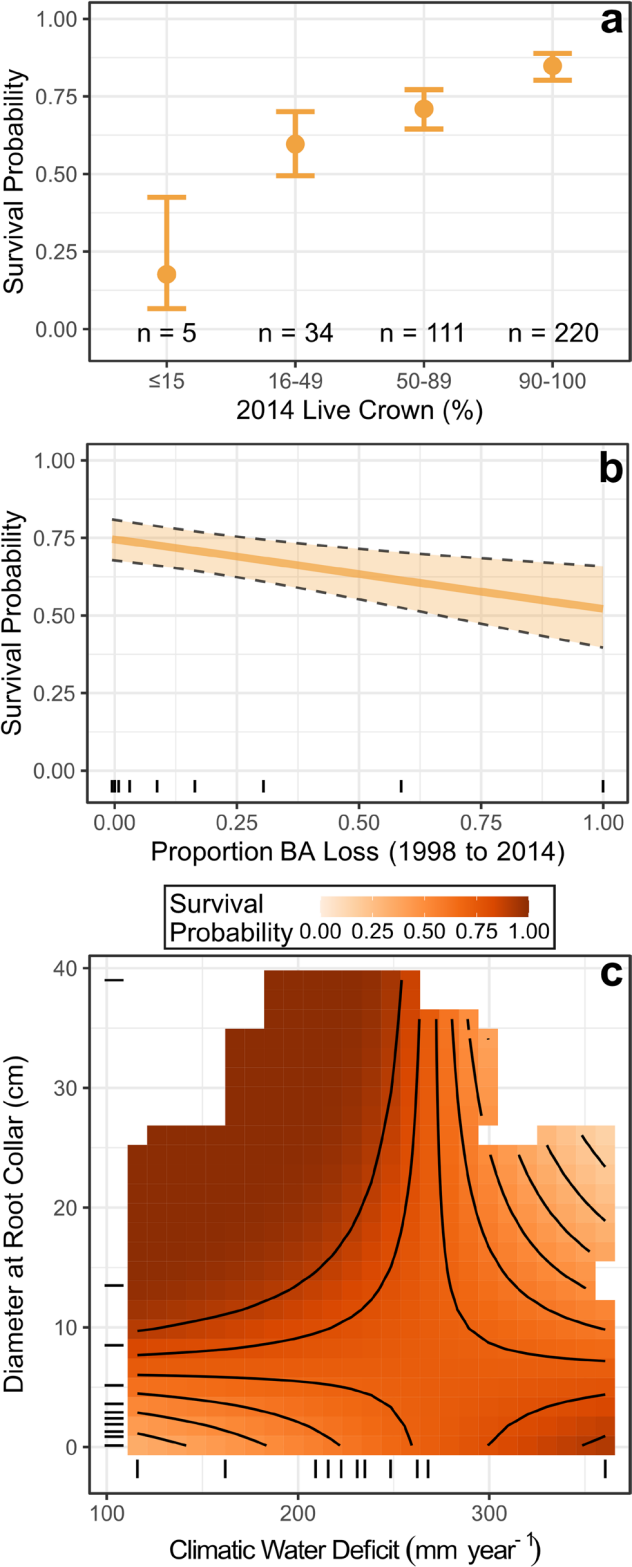
Results from the model of two‐needle pinyon ( *Pinus edulis* ) survival at long‐term monitoring sites in northern Arizona, USA. Individual panels show model‐predicted values of tree survival probability across the ranges of individual covariates (a, b) or pairs of covariates (c), assuming the mean values of other model terms. In (b, c), tick marks along the *x*‐ and *y*‐axes show the decile distributions of observed data, and background shading in (c) is removed in areas without observations. Error bars (a) and confidence bands (b) show the mean prediction ±1 standard error.

The top model of pinyon survival (Model #1) included Live Crown, Average CWD, DRC, and Proportion BA Loss (Figure 5, Tables S4.2–S4.4). Survival was positively associated with Live Crown, indicating that trees with full, healthy crowns prior to 2014 were more likely to survive from 2014 to 2023 (Figure 5a). Similarly, trees on plots without previous mortality had the highest survival probabilities (Figure 5b). A two‐way interaction between Average CWD and DRC indicated that intermediate to large pinyon trees (i.e., DRC > 15 cm; Figure S4.1d) were less likely to survive on hot, dry sites (i.e., CWD > 300 mm year^−1^), whereas small trees had relatively low survival across all sites (Figure 5c). Though this model was only marginally better than other competing models, the effects of Live Crown, Average CWD, DRC, and Proportion BA Loss were generally strong and consistent across models with partial support (Table S4.2). Fixed effects in the model explained 15% of the variation in pinyon survival (*R* ^2^_m_ = 0.15; AUC_m_ = 0.70) and random effects explained an additional 14% (*R* ^2^_c_ = 0.29; AUC_c_ = 0.86).

**FIGURE 6:**
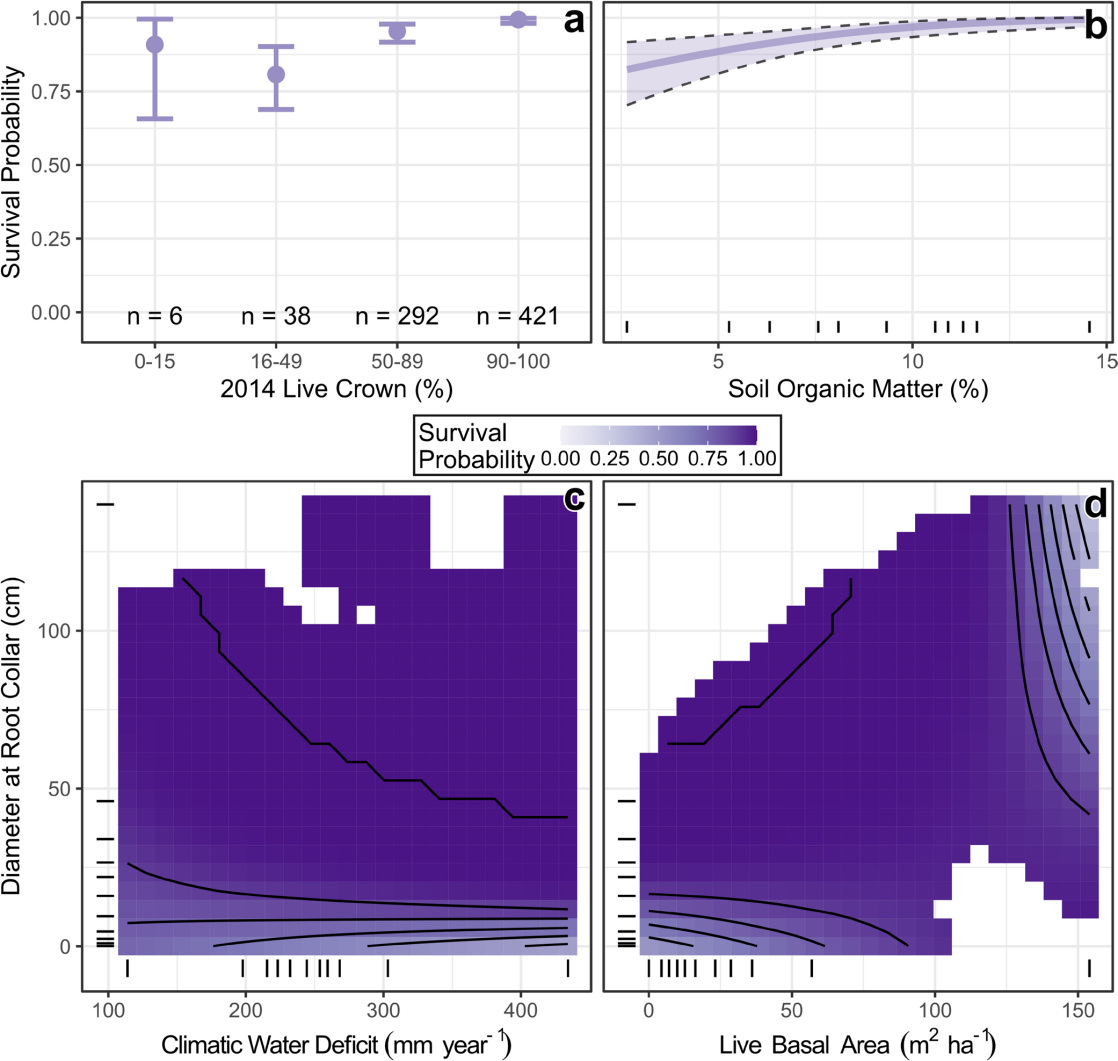
Results from the model used to predict one‐seed juniper ( *Juniperus monosperma* ) survival at long‐term monitoring plots in northern Arizona, USA. Individual panels show model‐predicted values of tree survival probability across the ranges of individual covariates (a, b) or pairs of covariates (c, d), assuming the mean values of other model terms. In (b, c, d), tick marks along the *x*‐ and *y*‐axes show the decile distributions of observed data, and background shading in (c, d) is removed in areas without observations. Error bars (a) and confidence bands (b) show the mean prediction ±1 standard error.

The top model of juniper survival (Model #2) included Live Crown, Soil OM, Average CWD, DRC, and Live BA (Figure 6, Tables S4.5–S4.7). Survival was greatest for individuals with high Live Crown in 2014 (Figure 6a). Soil OM was positively related to survival (Figure 6b), suggesting that trees growing on sites with deep litter layers and high soil OM were less prone to mortality. An interaction between Average CWD and DRC indicated that smaller trees (i.e., DRC < 15 cm; Figure S4.1i) were more sensitive to climatic conditions, whereas large trees survived at relatively high rates across all site types (Figure 6c). Finally, an interaction between Live BA and DRC suggested that smaller trees benefited from higher plot‐scale BA, whereas the largest trees (i.e., > 50 cm DRC) were more likely to survive in open areas (Figure 6d). Proportion BA Loss was included in several models with partial support and had a negative relationship with survival, suggesting that trees on plots with prior mortality were most vulnerable to subsequent drought (Table S4.5). Fixed effects in the model explained 32% of the variation in juniper survival (*R* ^2^_m_ = 0.32; AUC_m_ = 0.78) and random effects explained an additional 21% (*R* ^2^_c_ = 0.53; AUC_c_ = 0.95).

**FIGURE 7:**
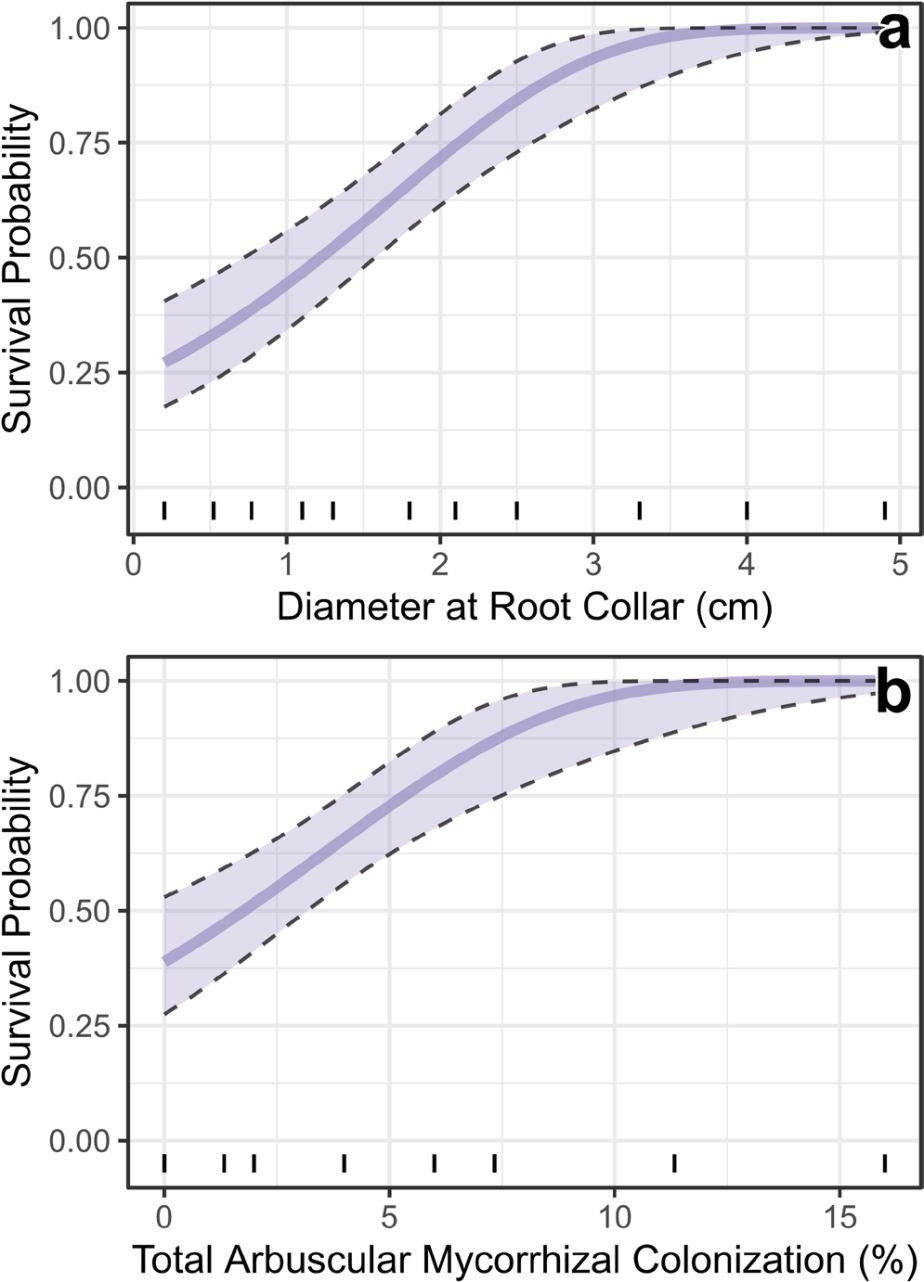
Results from the top model used to predict survival of small (< 5 cm DRC) one‐seed juniper ( *Juniperus monosperma* ) in long‐term monitoring plots in northern Arizona, USA. Individual panels show model‐predicted values of tree survival probability across the ranges of individual covariates, assuming the mean values of other model terms. Tick marks along the *x*‐axes show the decile distributions of observed data. Confidence bands show the mean prediction ±1 standard error.

The top model of small juniper (i.e., < 5 cm DRC) survival (Model #3) included DRC, Total AM Colonization, and no interaction between them (Figure 7, Tables S4.8–S4.10); survival increased with both DRC (Figure 7a) and AM colonization (Figure 7b). Fixed effects in this model explained 39% of the variation in survival (*R* ^2^_m_ = 0.39; AUC_m_ = 0.76) and random effects explained an additional 17% (*R* ^2^_c_ = 0.56; AUC_c_ = 0.90). Patterns of AM colonization on these sites may also help to explain broader trends between juniper survival and other biophysical factors. Total AM was positively associated with Soil OM (*p* = 0.02) and live juniper BA in 2014 (*p* < 0.01) and had a weakly negative relationship with Average CWD (*p* = 0.07) (Appendix S1).

### 3.3 Demographic Shifts in Woodlands and Susceptibility to Future Drought Events

**FIGURE 8:**
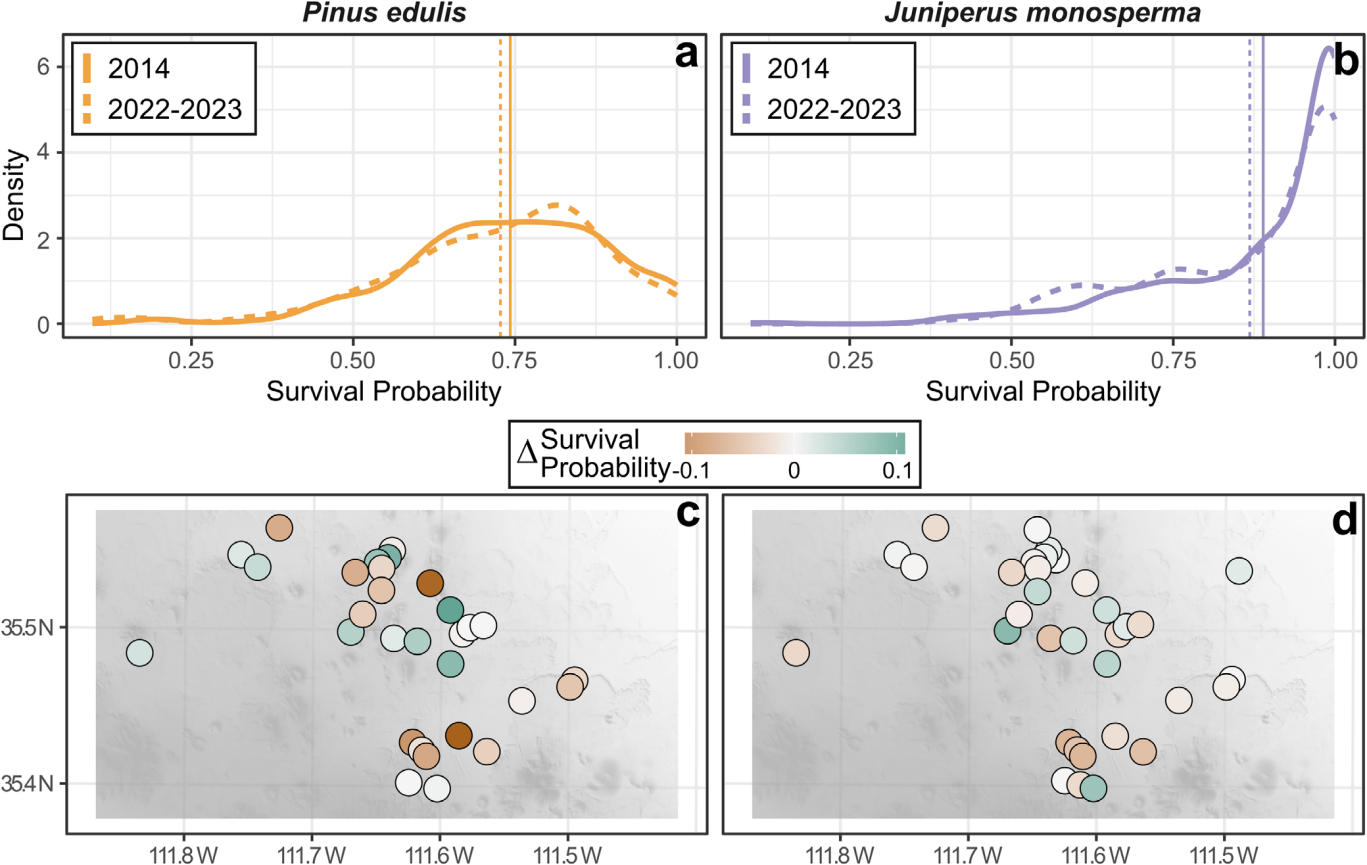
Changes in survival probability between 2014 and 2023 in pinyon ( *Pinus edulis* ; a, c) and juniper ( *Juniperus monosperma* ; b, d) woodlands of northern Arizona, USA. The distribution of tree‐level survival probabilities in (a, b) shows predictions for all live individuals present in 2014 (solid lines) or 2022–2023 (dashed lines). Mean survival probabilities in each time period in (a, b) are shown with vertical lines. Point colors in (c, d) show how changes in tree density, size structure, and crown dieback have altered mean survival probabilities of each species within individual sites.

Using fitted models in Figures 5 and 6, we predicted that tree‐level survival probabilities declined for each species between 2014 and 2023 (Figure 8). Specifically, average pinyon pine survival probability declined from 0.743 (2014; range = 0.735–0.750) to 0.727 (2023; range = 0.718–0.737); this represents a predicted increase in annualized mortality from 3.0% (2014) to 3.2% (2023) (Figure 8a). Likewise, average survival probability of juniper declined from 0.889 (range = 0.884–0.895) to 0.869 (range = 0.863–0.874), representing annualized mortality rates of 1.3% (2014) and 1.5% (2023) (Figure 8b). These patterns were variable across the landscape, with 53.3% of sites showing declines in pinyon pine survival probabilities (Figure 8c), and 61.8% showing declines for juniper (Figure 8d) between 2014 and 2023. Across species, survival probabilities declined slightly from 0.835 in 2014 (range = 0.830 to 0.839) to 0.827 in 2023 (range = 0.821–0.832), representing annualized mortality rates of 1.9% and 2.0% year^−1^, respectively (Figure S4.2).

These apparently subtle changes reflect two opposing processes—(1) selective mortality of more drought‐susceptible individuals (e.g., pinyon and smaller individuals of each species) and (2) an increase in vulnerability for individuals that survived. For example, 60.9% of trees that were alive in both 2014 and 2023 showed declines in survival probabilities over this period, relative to just 29.6% showing increases (the remaining 9.5% showed no change). Likewise, the average predicted survival probability of trees that were alive in both time periods declined from 0.876 in 2014 (range = 0.871–0.881; 1.5% mortality year^−1^) to 0.841 in 2023 (range = 0.836–0.847; 1.9% mortality year^−1^). This equates to a 28.2% relative increase in mortality risk for the live trees that survived between 2014 and 2023.

## 4 Discussion

Global increases in temperature and aridity are leading to more frequent drought events, which may impact forest and woodland ecosystems in complex ways (Anderegg et al. 2020; Müller and Bahn 2022; Moss et al. 2024; Figure 1). Whereas the interplay between ecological disturbances such as wildfire and insect outbreaks is comparatively well understood (e.g., Simard et al. 2011), interactions between drought‐caused tree mortality events remain an area of limited research, with broad implications for earth system models and ecosystem processes such as carbon storage (Anderegg, Schwalm, et al. 2015; Kannenberg et al. 2020). Leveraging a long‐running PJ monitoring network in the US Southwest, we found that drought events of the past three decades continue to have meaningful effects on woodland dynamics and that positive feedbacks are likely to increase the risk of future tree mortality. In contrast, we found no evidence of negative feedback dynamics in which initial drought‐caused mortality could ameliorate the impacts of subsequent events. Our analyses also demonstrated that size structure and community composition influence sensitivity to environmental conditions, edaphic factors play an important role in modulating tree survival, and crown foliage loss (i.e., drought‐caused dieback) may be a valuable indicator of gradual, longer‐term ecosystem degradation.

### 4.1 Positive Feedbacks and Size‐Dependent Responses

The structure and composition of PJ woodlands have changed rapidly in our study area since the 1990s, and this trend is likely to accelerate throughout the 21st century (McDowell et al. 2016; Noel, Schlaepfer, Butterfield, et al. 2025). Indeed, recent mortality and crown dieback of both pinyon and juniper have been widely documented across the US Southwest (Meddens et al. 2015; Kannenberg et al. 2021; Wion et al. 2022). Our findings (Figure 8) and those of prior research (e.g., Mueller et al. 2005; Flake and Weisberg 2019) suggest that such changes will trigger positive feedback mechanisms that amplify future tree mortality risk. Beyond these increases in tree mortality, drought can inhibit a wide range of additional processes in dry woodlands, such as seed production (Redmond et al. 2012; Wion et al. 2025), seedling recruitment (Shriver et al. 2022), tree radial growth (Williams et al. 2013; Evans et al. 2024), and defense against insect colonization (Gaylord et al. 2013; Trowbridge et al. 2021). Drought‐caused changes in these processes are likely to outweigh any negative feedbacks (Figure 1) and overwhelm system inertia that has contributed to longer‐term population gains over the past century (e.g., Shriver et al. 2025), driving transitions towards woody shrubs or herbaceous vegetation (e.g., Rodman et al. 2022; Noel et al. 2023, Noel, Schlaepfer, Barrett, et al. 2025).

However, not all PJ woodlands are equally vulnerable to decline, and size structure and community composition can strongly influence drought sensitivity. For example, in our study area, we found that small pinyon or juniper (i.e., < 5 cm DRC) had three times higher mortality rates than other trees between 2014 and 2023 (29.4% vs. 10.1%). These high levels of recent seedling mortality, combined with limited regeneration (i.e., < 15% of monitoring plots had new tree seedling establishment), indicate that seedling establishment is a key bottleneck for future woodland persistence (Redmond et al. 2015; Shriver et al. 2022; Noel et al. 2023). The sensitivity of small trees to environmental conditions also differs strongly between species. For example, small pinyon pines had relatively high rates of mortality across a range of site types (Figure 5c), whereas small junipers were most likely to die on dry sites (Figure 6c). One possible explanation for this interspecific difference is that small pinyons are often found beneath overstory trees and shrubs (Redmond et al. 2018), which help to moderate air temperatures (Urza et al. 2019). In contrast, juniper seedlings can be found in open areas without this microclimatic buffering effect and may be more sensitive to broad‐scale environmental variation.

Large pinyon trees (i.e., > 40 cm DRC) also had high mortality rates throughout our study period (Figure S4.1). This large‐tree mortality is likely due to the hydraulic limitations of tree size in moisture‐limited systems (Bennett et al. 2015; Trugman et al. 2021), as well as the preferential colonization of larger pinyon trees by the bark beetle species *Ips confusus* (Floyd et al. 2015; Negrón and Wilson 2003). Indeed, the mortality of large pinyon pine disproportionately occurred on dry sites (Figure 5c), where individuals may be predisposed to both hydraulic failure and bark beetle colonization due to more stressful baseline conditions. In contrast, large juniper had low mortality rates across a range of site conditions (Figure 6c). Individual size influences functional traits and the relationships between individuals and their environment (Violle et al. 2012; Barton 2024). This is particularly true for trees, where both size and species are related to traits such as root structure and depth that can influence drought resistance and the ability to obtain soil resources (Trugman et al. 2021). Such interactions highlight challenges in predicting future dynamics in dry woodlands, which will vary based on population demographics and community structure.

### 4.2 Plant–Soil Interactions and Crown Dieback Are Potential Mechanisms Driving Feedbacks

Plant–soil interactions are important mechanisms driving positive drought feedbacks in PJ woodlands. For example, survival of one‐seed juniper, now the dominant species across our study area, is positively related to soil OM (Figure 6b). In PJ woodlands of the US Southwest, OM is greatest in areas with tree cover and is dominated by nutrient pools with short residence times (Neff et al. 2009), which could rapidly decline following tree mortality events (Xiong et al. 2011). Thus, edaphic conditions could become less suitable for juniper as tree cover declines. Likewise, positive relationships between arbuscular mycorrhizal (AM) colonization and juniper survival (Figure 7b) and between juniper BA and Total AM (Table S1.1) suggest that live junipers may help to support drought resistance in neighboring, conspecific individuals by increasing the prevalence of AM fungal symbionts. However, AM fungi are also associated with other species in these ecosystems, including many herbaceous plants and woody shrubs (Haskins and Gehring 2004); thus, AM fungi may persist even when juniper numbers decline. In contrast, pinyon pine is the primary host for EM fungi (not studied here) in our study area, and EM can show substantial declines following drought‐related tree mortality (Swaty et al. 2004; Kilpeläinen et al. 2017; Hopkins et al. 2018). Furthermore, EM fungi have a higher carbon cost to trees than AM fungi (Olsson et al. 2010), and this association may challenge the ability of pinyon pine to survive under stressful conditions. Thus, differences in mycorrhizal associations may help to explain increases in juniper dominance at our sites and other areas across the US Southwest. Such changes in mycorrhizal communities and shifts towards AM‐associated tree species are part of a larger global trend related to the increasing frequency and severity of drought events (Swaty et al. 2004; Hopkins et al. 2018; Liao et al. 2025).

Patterns of crown dieback may be useful to identify and predict future tree mortality in dry woodlands. Considerable work has focused on climatic thresholds associated with tree mortality in forest and woodland ecosystems (Williams et al. 2013; Anderegg, Flint, et al. 2015; Law et al. 2019; Rodman et al. 2022; Wion et al. 2022). Our results suggest that identifying locations that have experienced recent physiological stress using remotely sensed observations of crown dieback may help to refine such predictions. Dieback is a common response to drought in woodlands, particularly for juniper species (Gaylord et al. 2013), and can be influenced by community structure and environmental stress gradients (Flake and Weisberg 2019; Kannenberg et al. 2021). In our study area, nearly half of the trees that survived from 2014 to 2023 showed evidence of recent crown dieback. Declines in live foliage can be mapped using methods such as aerial surveys from fixed‐wing aircraft (Coleman et al. 2018) or optical remote sensing techniques (Senf et al. 2015; Campbell et al. 2020), helping to identify woodlands that are particularly vulnerable to future drought. Such information may support management prioritization schemes such as the resist–accept–direct (RAD) framework to help identify opportunities for intervention (Schuurman et al. 2022). For example, using mechanical treatments or tree planting to resist or direct transitions before overstory tree mortality occurs, or planning for and accepting transitions towards shrubs or herbaceous vegetation on marginal sites (Noel et al. 2023; Noel, Schlaepfer, Butterfield, et al. 2025; Redmond et al. 2023).

### 4.3 Management Implications

Active management of forests and woodlands of the US West has been proposed as a method for reducing crown‐fire risk, increasing understory herbaceous cover, and enhancing tree vigor and resilience (Allen et al. 2002; Davis et al. 2024; Phillips et al. 2024). In some cases, such actions are believed to forestall change and “buy time” for local adaptation to changing climatic conditions (Bradford and Bell 2017; Noel et al. 2023; Rodman, Bradford, et al. 2025). Best management practices in PJ woodlands, however, are both a point of contention and uncertainty. On the one hand, facilitation by nurse structures plays a critical role in tree regeneration throughout PJ woodlands (Floyd 1982; Everett et al. 1986; Redmond et al. 2018); smaller pinyons and junipers likely benefit from facilitation in dense stand conditions (Floyd et al. 2015). However, these relationships may become neutral or competitive as individuals increase in size (Miriti 2006; Urza et al. 2019). Indeed, our results suggest that a decline in tree cover may reduce small‐tree survival through loss of facilitative effects, but we also observed potential benefits of open conditions to the largest trees (Figure 6d). Tree loss may also influence hydrological properties, which will become increasingly important in a more arid future. For example, hydraulic lift of soil moisture by tree roots to the shallow subsurface strata can be a stabilizing process in savanna and woodland ecosystems that declines following tree removal or mortality (Yu and D'Odorico 2015; Morillas et al. 2017). Thus, management actions to reduce PJ woodlands densities may increase drought resistance in some areas (Noel et al. 2023) but have a neutral or detrimental effect in others. PJ woodlands contain a wide array of habitat types, successional stages, disturbance regimes, and structural conditions that preclude a one‐size‐fits‐all approach to management (Baker and Shinneman 2004; Romme et al. 2009). Experimental research using management actions to promote drought resistance and foster ecosystem resilience is exceedingly rare in PJ woodlands (Redmond et al. 2023; but see Huffman et al. 2019), yet such work will be critical to inform management actions in a warmer, drier future.

### 4.4 Study Limitations

Our study period coincided with the most extreme dry period of the last 1200 years (Williams et al. 2022), but was preceded by the wettest period in the same time frame (Cook et al. 2025). These climatic events are overlaid upon longer‐term population gains in many PJ woodlands over the past several centuries (Shriver et al. 2025). Taken together, these processes may have led to the colonization of suboptimal microsites and structural overshoot of individuals (Greenwood and Weisberg 2008; Jump et al. 2017) immediately before a 20‐year megadrought. This unique combination of events makes it challenging to use data from this period to forecast future dynamics. Thus, we focused our modeling on recent and near‐term‐future conditions, rather than longer‐term projections into the future. Likewise, this study focused on the interactive effects of extreme, short‐interval drought events during an exceptionally dry period (Figure 2b). It is plausible that negative feedback processes would have been more evident during a period in which trees had sufficient time and resources for recovery from physiological impairment after initial drought. In addition, though we utilized a long‐term monitoring network, 25 years is still only a small snapshot of the dynamics of PJ woodlands, where trees grow slowly, individuals are long‐lived, and infrequent events can have lasting effects on longer‐term processes. Thus, continued research from an interdisciplinary perspective, including Indigenous knowledge, historical photography (Clifford et al. 2011; Vankat 2022), and paleoecology (Shriver et al. 2025) would be valuable to develop a more comprehensive picture of long‐term woodland change.

## 5 Conclusion

Extreme drought events can have dramatic and long‐lasting effects on dry woodlands. Here, we demonstrated that drought events in the US Southwest can trigger crown dieback, tree mortality, and alter other important components of woodland ecosystems. These effects, in turn, can drive positive feedback mechanisms that increase vulnerability to future drought events. We also found that patterns of recent tree mortality and dieback may help to forecast near‐term future changes in arid woodlands, which can aid in the planning and implementation of climate‐informed management frameworks. More broadly, positive feedbacks have now been identified across a range of temperate forests and woodlands dominated by coniferous trees (Anderegg et al. 2020). As drought events are expected to become more frequent and severe over the upcoming decades (Müller and Bahn 2022; Moss et al. 2024), these feedbacks have critical implications for models of vegetation dynamics from local to global scales. Indeed, future climatic conditions may trigger nonlinear responses in woodlands due to processes such as climate memory (Anderegg, Schwalm, et al. 2015) and altered population and community structure (Clark et al. 2016). Finally, our findings illustrate that often‐overlooked components of ecosystems (e.g., individual ontogeny, community composition, plant–soil interactions) are likely to shape dryland responses throughout a warmer, drier future.

## Author Contributions

**Kyle C. Rodman:** conceptualization (lead), data curation (equal), formal analysis (lead), investigation (lead), methodology (equal), project administration (lead), software (lead), visualization (lead), writing – original draft (lead), writing – review and editing (lead). **Kaylie J. Wilkerson:** conceptualization (supporting), data curation (supporting), formal analysis (supporting), investigation (supporting), methodology (supporting), writing – original draft (supporting), writing – review and editing (supporting). **Andreas P. Wion:** conceptualization (supporting), data curation (supporting), methodology (supporting), writing – original draft (supporting), writing – review and editing (supporting). **David W. Huffman:** conceptualization (supporting), methodology (supporting), project administration (supporting), writing – original draft (supporting), writing – review and editing (supporting). **Anita J. Antoninka:** conceptualization (supporting), data curation (supporting), methodology (supporting), project administration (supporting), resources (supporting), supervision (supporting), writing – original draft (supporting), writing – review and editing (supporting). **Mariola Barrera:** conceptualization (supporting), methodology (supporting), visualization (supporting), writing – original draft (supporting), writing – review and editing (supporting). **Neil S. Cobb:** conceptualization (supporting), data curation (equal), methodology (supporting), writing – original draft (supporting), writing – review and editing (supporting). **Miranda D. Redmond:** conceptualization (supporting), data curation (equal), investigation (supporting), methodology (supporting), project administration (supporting), writing – original draft (supporting), writing – review and editing (supporting).

## Funding

This work was supported by the U.S. Forest Service, SW Forest Health and Wildfire Prevention Act.

## Conflicts of Interest

The authors declare no conflicts of interest.

## Supporting information


**Data S1:** ece372667‐sup‐0001‐Supinfo.pdf.

**Data S2:** ece372667‐sup‐0002‐Supinfo.pdf.

## Data Availability

All data, analytical codes, and statistical model outputs from this study are available through Zenodo (Rodman, Wilkerson, et al. 2025) using the following link: https://doi.org/10.5281/zenodo.16044413. A reproducible data document demonstrating key decisions in the analyses is provided as Supporting Information.
