# Supplementary material for: Positive Drought Feedbacks Increase Tree Mortality Risk in Dry Woodlands of the US Southwest
Source: Ecol Evol. 2025 Dec 10;15(12):e72667. doi: 10.1002/ece3.72667 (PMC12696023; doi:10.1002/ece3.72667)
Supplement: Supplementary file 1 — Data S1: ece372667‐sup‐0001‐Supinfo.pdf. [file ECE3-15-e72667-s002.pdf]

Reproducible Research Document for *'Positive drought feedbacks increase tree mortality risk in dry woodlands of the US Southwest'*

2025-10-30

Contents

1 Overview **2**

2 Data Wrangling and Processing **3**

2.1 "Step1a-CleanFieldData.R" 3
2.2 "Step1b-GetDaymet.txt" 3
2.3 "Step1c-GetTerrain.txt" 4
2.4 "Step1d-HLI_Calcs.R" 4
2.5 "Step1e-GIDS_Downscaling.R" 4
2.6 "Step1f-CWD_Calcs.R" 4
2.7 "Step1g-MergeData.R" 4
2.8 "Step1h-GetClimateDataForSiteMap.R" 4

3 Data used in model development **4**

3.1 Full_ID 5
3.2 Site 5
3.3 Section 6
3.4 TreeID 6
3.5 Species 6
3.6 DRC_cm 7
3.7 Height_cm 7
3.8 crown_class 8
3.9 liveBA_sqM_ha 8
3.10 BA_diff_sqM_ha_2014Minus2000 9
3.11 Total_AM_perc 9
3.12 OrganicMatter_Percent 10
3.13 meanCWD_1991_2020 10
3.14 wood 11
3.15 Survived 11

4 Model development and checking **12**

4.1 Model #1: Survival of pinyon pine 13

4.2 Model #2: Survival of juniper 19

4.3 Model #3: Small juniper survival 25

5 Using fitted model objects to make predictions of mortality risk **30**

6 References **36**

1 Overview

This document provides supplementary information describing our data, model development, and residual diagnostics for long-term permanent plots in pinyon-juniper woodlands of northern Arizona, USA. We completed a remeasurement of these sites in 2022 & 2023, following a widespread dieback and mortality event in the region. Our primary objective was to determine how initial (ca. 2000-2004) drought-caused changes in forest structure and composition at these sites influenced responses to the more recent drought event, and how such interactions might influence future mortality risk of arid woodlands throughout the US West. As shown in the figure below, there are reasons to believe that an initial drought, and drought-caused changes in forest conditions could either increase or decrease the risk of tree mortality.

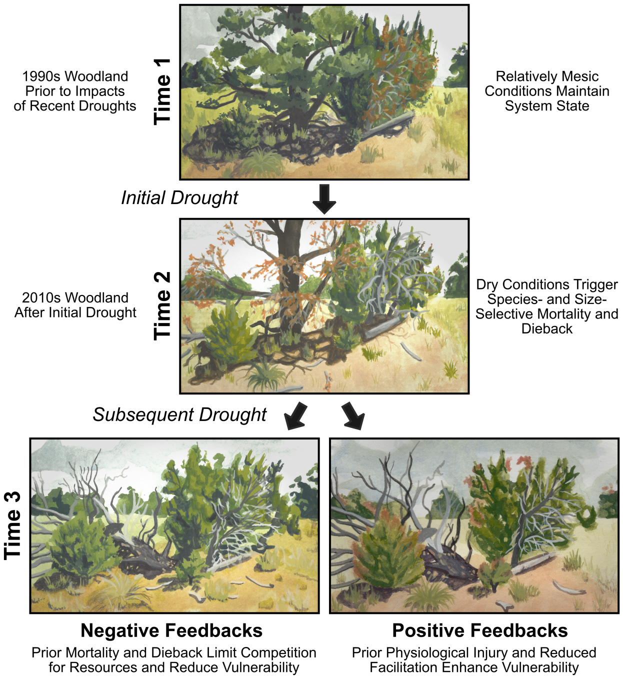

The sites were originally established to span topographic and edaphic gradients within the 1500 sq. km study area. However, due to the diverse environmental conditions of this area, they also span much of the climatic range of each species. Likewise, our study period (1998-2023) includes several intense drought years and some moderately wet years

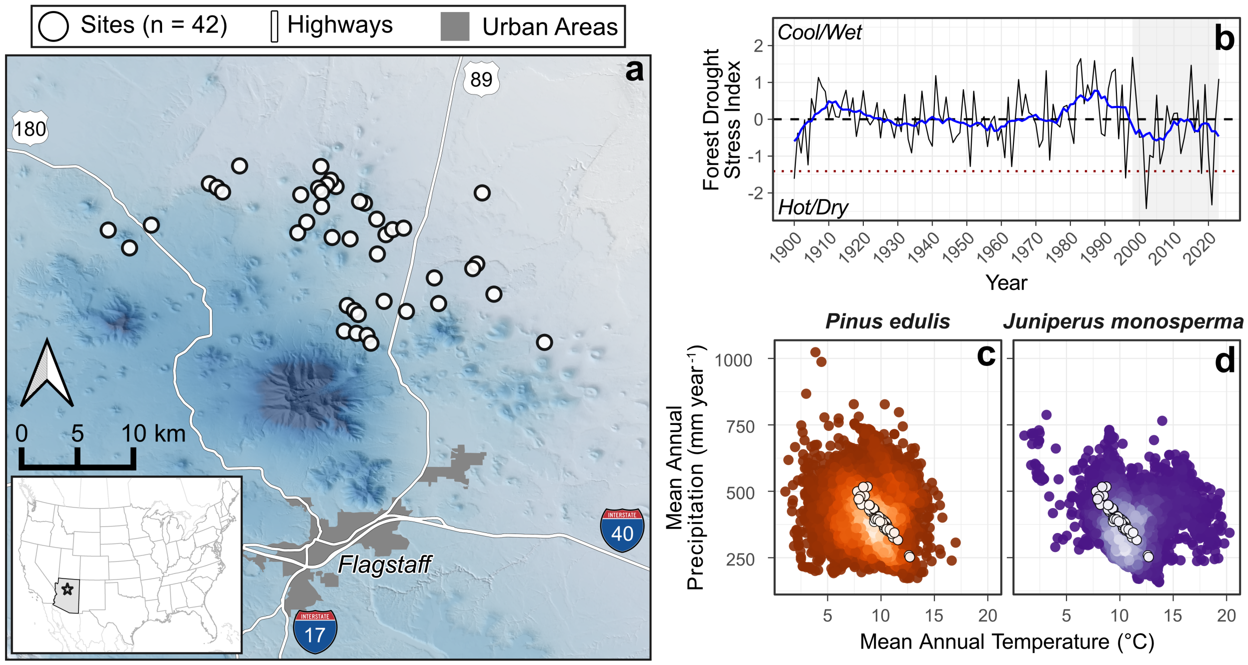

This reproducible research document is subdivided into several sections which provide (Sec 2) information on data wrangling and processing, (Sec 3) descriptions of the "analysis-ready" data used in models, (Sec 4) steps involved in model fitting and checking, and (Sec 5) how models were used to develop future projections. For additional information, all code, data files, and model outputs are provided in an archive through Zenodo, as described in the Data Availability Statement of the manuscript.

2 Data Wrangling and Processing

Data wrangling and processing in this project involved several steps: from cleaning and organizing field data, obtaining spatial data describing weather and terrain, using these data to develop model covariates, and merging field data and covariates to create "analysis ready" files used in subsequent sections. A detailed description of these steps is provided in the following scripts included in the data archive subfolder "\Code\Step1-DataPrep":

2.1 "Step1a-CleanFieldData.R"

This is the primary script used to clean and organize field data in R. It pulls in data from line-point intercept transects, tree lists, plot locations (spatial data), and soil surveys, merging them into summary files with tree- and plot-scale information. Several error checks are also performed to identify "jesus trees" that died in one survey and come back to life, identify irregular or unrealistic values in covariates, and filter out plots that experienced fire during our study period.

2.2 "Step1b-GetDaymet.txt"

This script, a Java-based file saved in the general .txt format, is used to download climate data from the Daymet dataset produced by Oakridge National Laboratory (https://daymet.ornl.gov/). The script was run using Google Earth Engine in the online code editor platform

2.3 "Step1c-GetTerrain.txt"

This script, a Java-based file saved in the general .txt format, is used to download a 10-m digital elevation model from the USGS 3DEP dataset (https://www.usgs.gov/3d-elevation-program), and also calculate slope and aspect (in degrees) using this file. The script was run using Google Earth Engine in the online code editor platform.

2.4 "Step1d-HLI_Calcs.R"

This script uses elevation, slope, and aspect grids described above to calculate heat load index (HLI) following equation 3 in McCune and Keon (2002). This file is not used directly as a covariate, but used to help calculate climatic water deficit (CWD) below.

2.5 "Step1e-GIDS_Downscaling.R"

This script spatially downscales monthly weather data from Daymet (total precipitation, minimum temperature, maximum temperature) from 1-km to 30-m resolution using the spatial Gradient and Inverse Distance Squared (GIDS) method of Nalder and Wein (1998) and Flint and Flint (2012). Source code for this R function was developed by Rodman et al. (2020).

2.6 "Step1f-CWD_Calcs.R"

This script uses inputs of soil available water capacity (AWC) in the top 200 cm of the soil profile from POLARIS (Chaney et al. 2016), along with HLI (developed in "Step1d…") and 30-m weather variables (developed in "Step1e…") to calculate climatic water deficit (CWD) using the modified Thornthwaite methods of Lutz et al. 2010 and using R source code of Redmond (2022).

2.7 "Step1g-MergeData.R"

This script extracts spatial variables (e.g., annual Climatic Water Deficit) at the locations of field plots or sites, and merges the various field-based datasets describing ground cover, soils, forest structure, and tree information. The result is the "analysis ready" files "PlotsForRegen.csv" and "TreesForSurvival.csv" which are described in further detail below.

2.8 "Step1h-GetClimateDataForSiteMap.R"

This script uses climate data from PRISM (PRISM Climate Group, 2023) to calculate the Forest Drought Stress Index (FDSI) of Williams et al. (2013). This index is a useful indicator of interannual variation in weather conditions (e.g., winter precipitation and summer vapor pressure deficit) that influence tree growth in the US Southwest. The script also compares average climate of the sites to locations throughout the range of *Juniperus monosperma* and *Pinus edulis* (Wilson et al. 2013), the two focal species in this study. These data summaries are only used in a descriptive way for the methods section of the paper, and in developing panels b, c, and d in the site map figure (Figure 2)

3 Data used in model development

This section provides a brief overview of the data used for model development in this project. We focus specifically on the "TreesForSurvival.csv" file developed in the "Step1g-MergeData.R" script. This is the primary file used for analysis in our project.

First, load a few necessary packages for this and the following code chunks.

```
### Bring in necessary packages
package.list <- c("here","tidyverse", "glmmTMB", "splines", "MuMIn", "performance",
                  "DHARMa", "ncf", "sf", "pROC", "terra", "tidyterra", "patchwork",
                  "RColorBrewer")

## Installing them if they aren't already on the computer
new.packages <- package.list[!(package.list %in% installed.packages()[,"Package"])]
if(length(new.packages)) install.packages(new.packages)

## And loading them
for(i in package.list){library(i, character.only = T)}
```

Next, lets read in the analysis-ready data file

```
## Trees for survival models
survTrees <- read_csv(here("Data", "AnalysisReady", "TreesForSurvival.csv"))
```

```
## Rows: 1140 Columns: 24
## -- Column specification --------------------------------------------------------
## Delimiter: ","
## chr  (3): Full_ID, TreeID, Species
## dbl (21): Site, Section, DRC_cm, Height_cm, crown_class, liveBA_sqM_ha, BA_d...
##
## i Use `spec()` to retrieve the full column specification for this data.
## i Specify the column types or set `show_col_types = FALSE` to quiet this message.
```

Note that this file is read in as a tibble using the dplyr package, and by default prints out a bit of information about it. This dataset is structured such that each row represents a single tree, and columns represent its fate between 2014 and 2022/2023, and several potential covariates. Here, we highlight some of the most important columns, however a full README.md file is provided in the data archive. The most important columns are as follows:

3.1 Full_ID

A unique identifier for each tree, which combines the site (referred to as a "transect" in the manuscript), section (referred to as a plot in the manuscript), and treeID, separated by "_" characters

```
## Summarizing the number of individuals. Note that this == nrow(survTrees)
length(unique(survTrees$Full_ID))
```

```
## [1] 1140
```

3.2 Site

Also referred to as a "transect" in the manuscript. This code refers to the belt transect (i.e., a cluster of ca. 10, 10x10m fixed-area plots) on which a tree was located. Note that this dataset includes only 34 of the original sites, as several were excluded from analysis due to recent fire or management activities

```r
## Summarizing the number of sites
length(unique(survTrees$Site))
```

```
## [1] 34
```

3.3 Section

Also referred to as a "plot" in the manuscript. This code refers to plot number (within a site) on which a tree was located. Numbers repeat across sites, so section numbers are only considered unique when merged with the site field. This analysis includes trees from 261 of the original plots. This subset includes only plots that had trees present in 2014, and were not burned or treated with forest management activities after 2014

```r
## Summarizing the number of unique plots within sites
length(unique(paste(survTrees$Site, survTrees$Section)))
```

```
## [1] 261
```

3.4 TreeID

This code refers to tree number (within a site and plot) of a given individual. It is used to track individuals across remeasurements. Numbers repeat across sites and plots, so tree numbers are only considered unique when merged with these two fields (i.e., "Full_ID"). Note that 1,140 unique values are present, which is == nrow(survTrees) & == length(unique(survTrees$Full_ID))

```r
## Summarizing the number of unique tree IDs within sites and sections
length(unique(paste(survTrees$Site, survTrees$Section, survTrees$TreeID)))
```

```
## [1] 1140
```

3.5 Species

A one-letter code which identifies the species of an individual tree. J = *Juniperus monosperma* or one-seed juniper; P = *Pinus edulis* or two-needle pinyon. This dataset has already been restricted to these two species which comprise ca. 90% of all basal area and individuals at our sites. However, individuals of other species were used to calculate data summaries in certain fields, as described below. The table below shows the number of juniper and pinyon individuals in the analysis-ready data

```r
## Summarizing the number of individuals of each species
table(survTrees$Species)
```

```
##
##   J   P
## 764 376
```

3.6 DRC_cm

The field-measured diameter at root collar (DRC) of an individual in either 1998/2001 (i.e., plot installation) or 2014 measurements, whichever was considered to be the most reliable measurement (as used in Redmond et al. 2015). The boxplot below shows the distribution of DRC values by species.

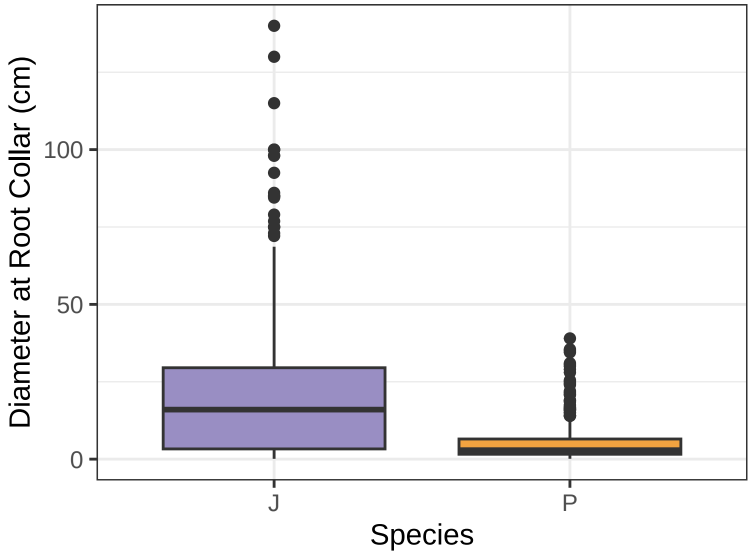

3.7 Height_cm

The field-measured height of an individual in either 1998/2001 or 2014, whichever was considered to be the most reliable measurement (as used in Redmond et al. 2015). The boxplot below shows the distribution of height values by species.

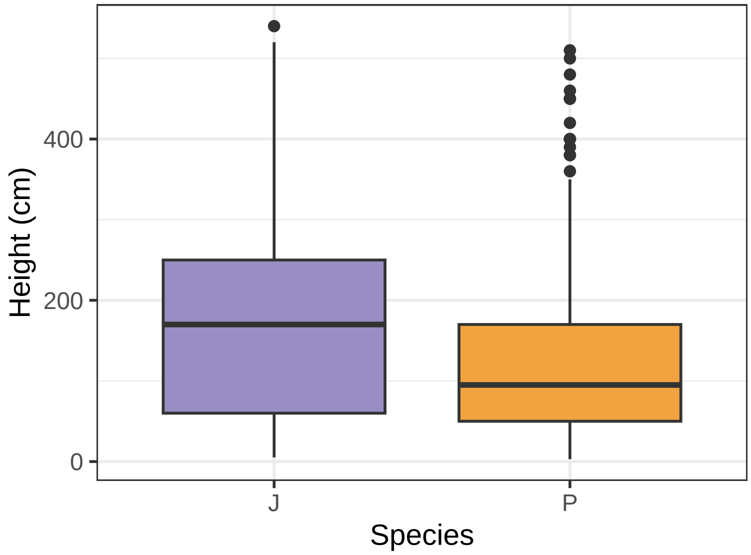

3.8 crown_class

Referred to as “Live Crown” in the manuscript. Percentage of tree crown with live foliage, recorded in the field in 2014. Subdivided into 4 categories: 4 (< 15%), 3 (16-50%), 2 (51-90%), and 1 (> 90%).

```
## Summarizing the number of individuals within each Live Crown class and species
table(survTrees$Species, survTrees$crown_class)
```

```
##
##         1   2   3   4
##    J 421 292  38   6
##    P 220 111  34   5
```

```
## Or alternatively, in the more meaningful percentage values
survTrees$crown <- case_when(
        survTrees$crown_class == "1"  ~ ">= 90",
        survTrees$crown_class == "2"  ~ "50-89",
        survTrees$crown_class == "3"  ~ "16-49",
        survTrees$crown_class == "4"  ~ "<= 15")
table(survTrees$Species, survTrees$crown)
```

```
##
##      <= 15 >= 90 16-49 50-89
##    J     6   421    38   292
##    P     5   220    34   111
```

```
## And remove the temp column
survTrees$crown <- NULL
```

3.9 liveBA_sqM_ha

Cross-sectional area (sq. m/ha) of all live trees present in a plot in 2014. Calculated using DRC^2 * 0.00007854 * 100. This formula converts measured DRC values to cross-sectional area (in meters) and then converts them to units/ha using an expansion factor (100) based on the plot size (0.01 ha). The following boxplot shows how these values are distributed on plots that contain individuals of each species.

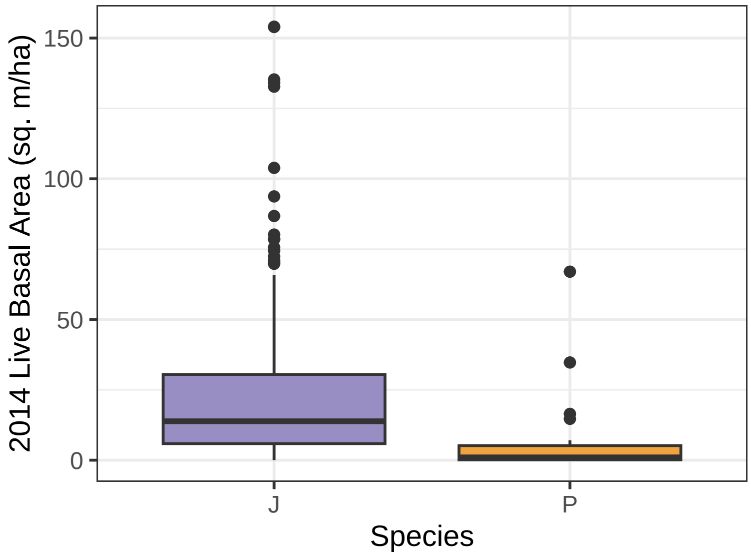

3.10 BA_diff_sqM_ha_2014Minus2000

Used to calculate "Proportion BA Loss" in the manuscript (calculated as 1-(liveBA_sqM_ha/(liveBA_sqM_ha-BA_diff_sqM_ha_2014Minus2000)). The change in live BA present in a plot between study initiation (i.e., 1998-2001) and 2014, where greater negative values indicate greater declines. The following boxplot shows how these values are distributed on plots that contain individuals of each species.

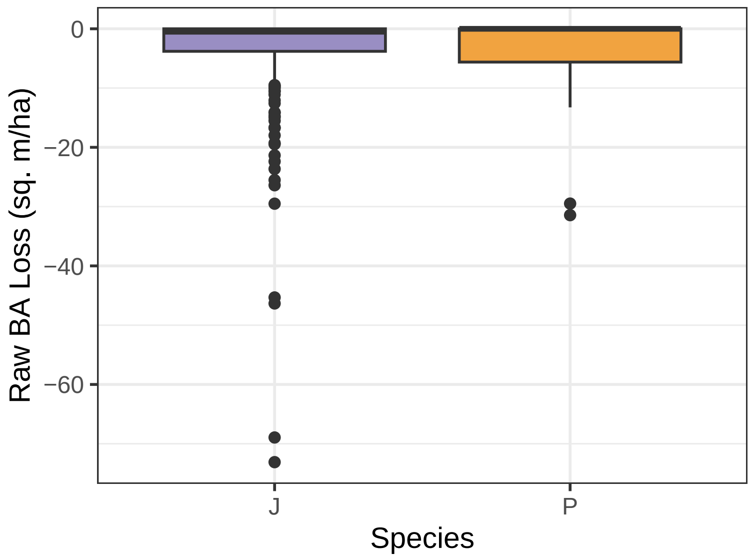

. . . and the variable used in analyses

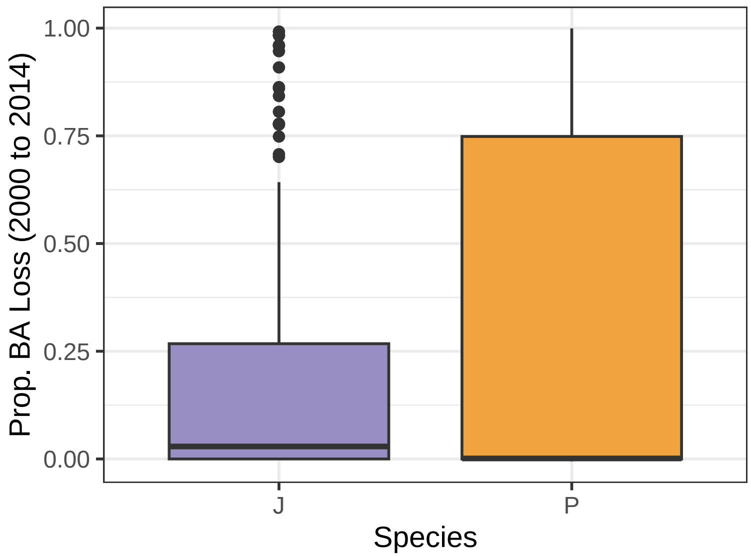

3.11 Total_AM_perc

The percentage of root tips with evidence of AM colonization based on soil samples collected in 2023. Further detail on methods of sample collection and processing are provided in Appendix S1 associated with this manuscript. The following boxplot shows how these values are distributed on plots that contain small juniper (the focus of analyses using these data).

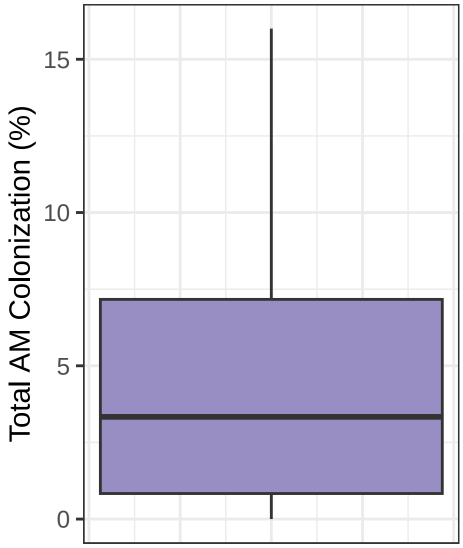

3.12 OrganicMatter_Percent

Percentage of the top 10 cm of the soil horizon composed of organic matter, based on field-derived soil samples collected in 2014. The following boxplot shows how these values are distributed on plots that contain individuals of each species.

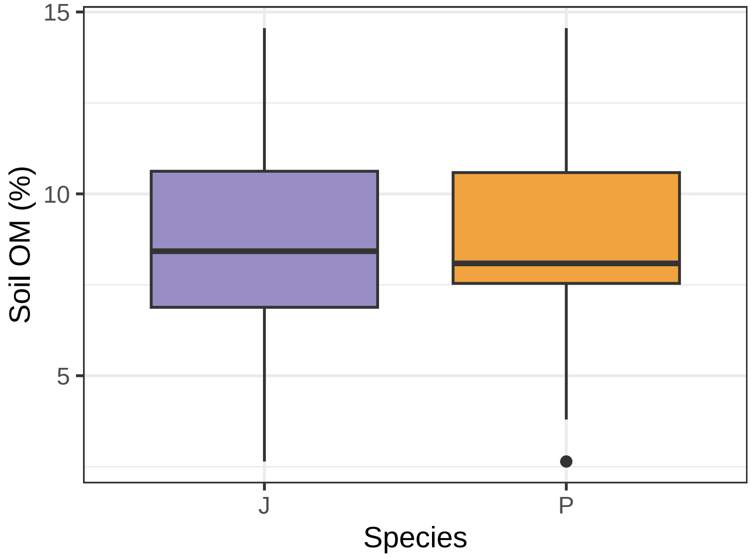

3.13 meanCWD_1991_2020

Climatic water deficit represents the unmet evaporative demand of the atmosphere (average mm/yr for the 1991-2020 period), estimated from the monthly water balance model of Lutz et al. (2010) as implemented in “Step1f. . . ” script above using GIS-derived soils, topography, and weather data. The following boxplot shows how these values are distributed on plots that contain individuals of each species.

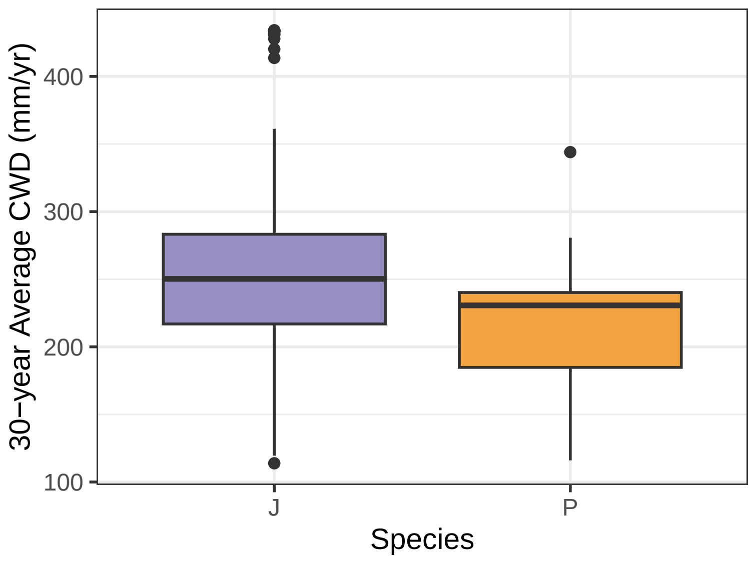

3.14 wood

The proportion of the ground surface covered in woody debris (i.e., > 1 cm diameter) during 2014 measurements. Recorded in the field using line-point intercept transects. The following boxplot shows how these values are distributed on plots that contain individuals of each species.

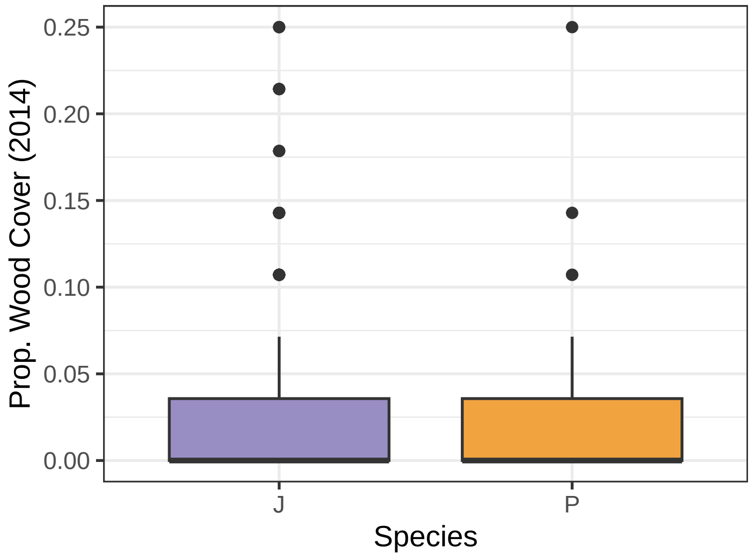

3.15 Survived

Response variable in our models. Whether a tree (initially alive in 2014) survived to remeasurements in 2022/2023. Values: 1 = Survived, 0 = Died. The following table shows the fate of individuals in the data by species

```
## Summarizing the number of individuals of each species
table(survTrees$Species, survTrees$Survived)
```

```
##
##       0   1
##   J 111 653
##   P  99 277
```

4 Model development and checking

Next, we will show examples of model fitting and evaluation used to develop statistical models for this study. Further detail on these steps is provided in "\Code\Step2\Step2-AnalysisAndDataVis\Step2b-SurvivalAnalysis.R" and "\Code\Step2\Step2-AnalysisAndDataVis\Step2c-AnalysisPlots.R". Some additional descriptive plots/analyses are performed in "\Code\Step2-AnalysisAndDataVis\Step2a-DescriptivePlots.R", but are not discussed at length here. The three models are:

- **Model #1**: A model of survival for all two-needle pinyon pine that were (1) alive in 2014, (2) on plots that were remeasured in 2022/2023, and (3) > 0.5 cm DRC OR > 5 cm in height. The full model, including all potential covariates, follows the general form of Survival ~ Average CWD * DRC + Live BA * DRC + Live BA * Prop BA Loss + Soil OM + Wood + Live Crown + (1|Site/Section) where "*" symbols indicate an interaction term, and (1|Site/Section) refers to a nested random intercept term of plot within transect (to account for dependence among individuals within a plot, and plots within a transect). The model is specified using a binomial error structure.
- **Model #2**: A model of survival for all one-seed juniper that were (1) alive in 2014, (2) on plots that were remeasured in 2022/2023, and (3) > 0.5 cm DRC OR > 5 cm in height. The full model, including all potential covariates, follows the general form of Survival ~ Average CWD * DRC + Live BA * DRC + Live BA * Prop BA Loss + Soil OM + Wood + Live Crown + (1|Site/Section) where "*" symbols indicate an interaction term, and (1|Site/Section) refers to a nested random intercept term of plot within transect (to account for dependence among individuals within a plot, and plots within a transect). The model is specified using a binomial error structure.
- **Model #3**: A model of survival for small juniper (i.e., < 5 cm DRC) that were (1) alive in 2014, (2) on plots that were remeasured in 2022/2023, (3) had data describing Total AM colonization, and (4) > 0.5 cm DRC OR > 5 cm in height. The full model, including all potential covariates, follows the general form of Survival ~ DRC * Total AM + (1|Site/Section) where "*" symbols indicate an interaction term, and (1|Site/Section) refers to a nested random intercept term of plot within transect (to account for dependence among individuals within a plot, and plots within a transect). The model is specified using a binomial error structure.

With each of these models, we used "all subsets" model selection (using the MuMIn::dredge() function in R) to identify the combination of fixed-effects terms that minimized AICc. We then performed standard tests of residual distributions and spatial independence of residuals using the DHARMa and ncf packages in R. All steps of model fitting and checking are shown below, with separate subsections for each model

But first, reading in the other datasets, and doing a few pre-processing steps

```
##############################################################################
### Read in files

## Plot locations to check for spatial autocorrelation
plots <- st_read(here("Data", "Spatial", "SiteLocations",
                      "plotCenters_5_11_22_WGS84.shp"), quiet = T) %>%
  st_transform(crs = "+proj=utm +zone=12 +datum=NAD83 +units=m +no_defs +type=crs")
      ## Converting to UTMs so that distances are in metres, not decimal degrees

## Get list of all juniper trees in recent survey to calculate live juniper BA
```

```r
allJ <- read_csv(here("Data", "Processed", "AllTrees_corrected.csv")) %>%
  filter(Species == "J" & Status == "L" & Year == 2014)

################################################################################
### Some data prep

## Modifying two variables for analysis
survTrees$propBA_Loss <- with(survTrees,
                              1-(liveBA_sqM_ha/
                                   (liveBA_sqM_ha-BA_diff_sqM_ha_2014Minus2000)))
survTrees$propBA_Loss <- ifelse(survTrees$propBA_Loss <0, 0, survTrees$propBA_Loss)
survTrees$crown_class <- factor(survTrees$crown_class,
                                levels = c("4", "3", "2", "1"), ordered = T)
                  ## This is treated as an ordinal factor rather than an unordered categorical term

## Subsetting to include only complete rows, and filter by species for the two models
subTrees <- survTrees %>% filter(!is.na(crown_class)) ## Get rid of 13 rows without crown health info i[illegible]
pinyon <- subTrees %>% filter(Species == "P")
juniper <- subTrees %>% filter(Species == "J")

## Getting tree-level data on survival and fungal communities for model #3
mycoSurvivalTrees <- survTrees %>%
  filter(Species == "J" & DRC_cm < 5 & !is.na(DRC_cm) & !is.na(Total_AM_perc)) %>%
  dplyr::select(Site, Section, DRC_cm, Total_AM_perc, OrganicMatter_Percent,
                meanCWD_1991_2020, Survived) %>%
  mutate(Total_AM_Pts = round(Total_AM_perc/100*150), ## No. pts is out of a total of 150
         Total_noAM_Pts = 150 - Total_AM_Pts) ## So, No. of "failures" is 150 - number of AM points
```

4.1 Model #1: Survival of pinyon pine

For model #1, we go through the steps of fitting the full model. Note that we first test a simple non-linear effect of Average CWD (a natural spline with two degrees of freedom), with the idea that some intermediate value of climate may represent optimal conditions for survival. However, this term led to likely overfitting and was excluded here.

```r
## First, does non-linear term for CWD make sense? Ecologically, it might be that survival optima are a[illegible]
cwdMod1 <- glmmTMB(Survived ~
                     scale(meanCWD_1991_2020, scale = sd(meanCWD_1991_2020)*2) +
  (1|Site/Section), data = pinyon, family = binomial(link = "cloglog"))
cwdMod2 <- glmmTMB(Survived ~
                     ns(scale(meanCWD_1991_2020, scale = sd(meanCWD_1991_2020)*2), df = 2) +
                     (1|Site/Section), data = pinyon, family = binomial(link = "cloglog"))
AICc(cwdMod1, cwdMod2) ## Lower AICc in more complex model
```

```
##         df     AICc
## cwdMod1  4 413.8998
## cwdMod2  5 412.0197
```

```r
predict(cwdMod1, newdata = data.frame(meanCWD_1991_2020 = c(0,100,200,300,400,500,600),
                                      Site = "999", Section = "999"), allow.new.levels = T, type = "res[illegible]
```

```
## [1] 0.7672026 0.7669971 0.7667915 0.7665858 0.7663801 0.7661743 0.7659684
```

```r
predict(cwdMod2, newdata = data.frame(meanCWD_1991_2020 = c(0,100,200,300,400,500,600),
                                      Site = "999", Section = "999"), allow.new.levels = T, type = "resp
```

```
## [1] 0.7980647 0.7441012 0.7087532 0.6995087 0.7208010 0.7690282 0.8347440
```

```r
# While AICc is improved, we should use simpler model. Lower survival in intermediate climates isn't re

## Fit full pinyon model -- all variables are scaled in the manner of Gelman (2008) to permit compariso
pinyonSub <- pinyon %>% filter(!is.na(wood)) ## Removing a few rows without cover/soils data, since the
pinyonMod <- glmmTMB(Survived ~ scale(DRC_cm, scale = sd(DRC_cm)*2)*
                       scale(liveBA_sqM_ha, scale = sd(liveBA_sqM_ha)*2) +
                       scale(propBA_Loss, scale = sd(propBA_Loss)*2)*
                       scale(liveBA_sqM_ha, scale = sd(liveBA_sqM_ha)*2) +
                       scale(meanCWD_1991_2020, scale = sd(meanCWD_1991_2020)*2)*
                       scale(DRC_cm, scale = sd(DRC_cm)*2) +
                       crown_class + ## Removing CC * size interaction which causes convergence errors
                       scale(wood, scale = sd(wood)*2) +
                       scale(OrganicMatter_Percent, scale = sd(OrganicMatter_Percent)*2) +
                      (1|Site/Section), data = pinyonSub, family = binomial(link = "cloglog"),
                     na.action = "na.fail")
```

Next, we perform model selection using this fitted model object

```r
## Model selection using all subsets
#pinyonModSelection <- dredge(pinyonMod)

## Based on model selection, the top model follows the form "Survived ~ crown_class +
  # propBA_Loss + meanCWD_1991_2020*DRC_cm + (1|Site/Section), so fit this model again
finalPinyonMod <- glmmTMB(Survived ~ crown_class +
                            scale(propBA_Loss, scale = sd(propBA_Loss)*2) +
                            scale(meanCWD_1991_2020, scale = sd(meanCWD_1991_2020)*2)*
                            scale(DRC_cm, scale = sd(DRC_cm)*2) + (1|Site/Section),
                          data = pinyon, family = binomial(link = "cloglog"))
                      ## Here, we refit it with the full dataset, because ground cover and
                      # other variables with missing values had no effect in the model
```

Using the final model, we test a few residual diagnostics, autocorrelation, and quantify goodness of fit using the MuMIn::r.squaredGLMM ar pROC::AUC functions.

```r
## Testing assumptions. All look good

# Overall residuals and collinearity
resd <- simulateResiduals(finalPinyonMod)
plot(resd)
```

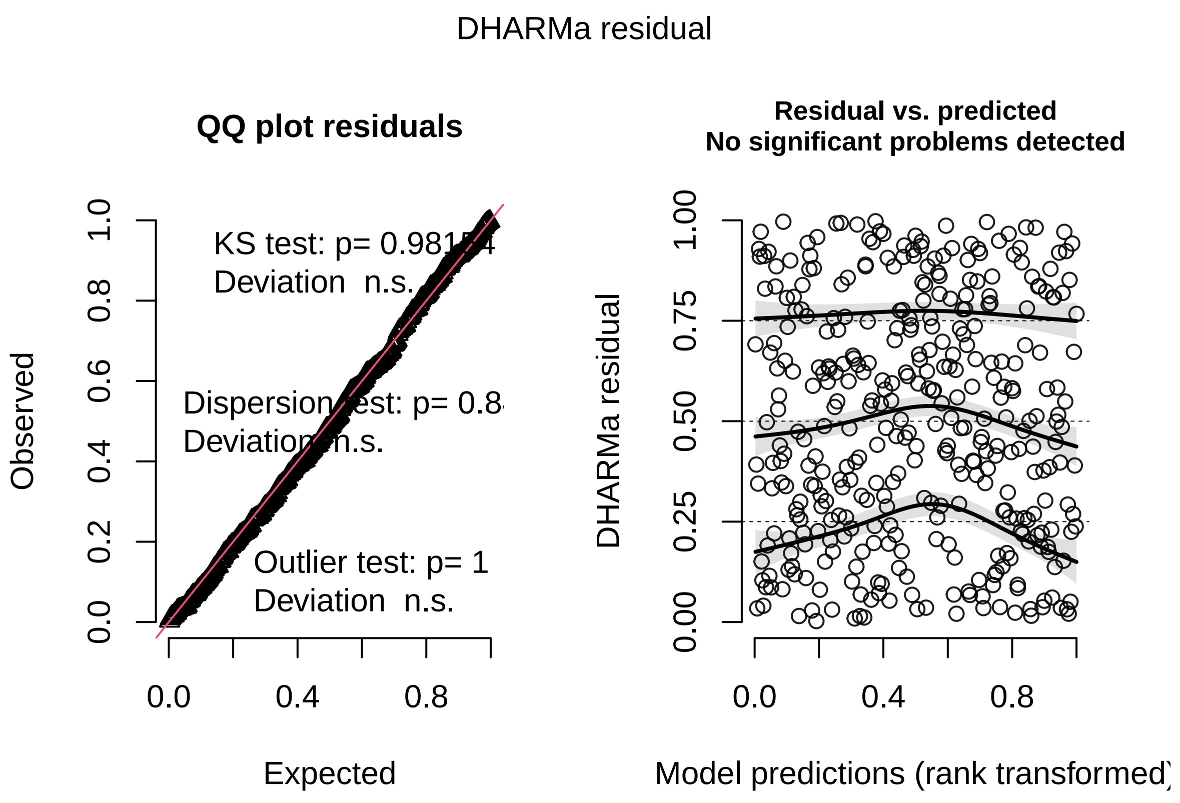


```r
testDispersion(resd)
```

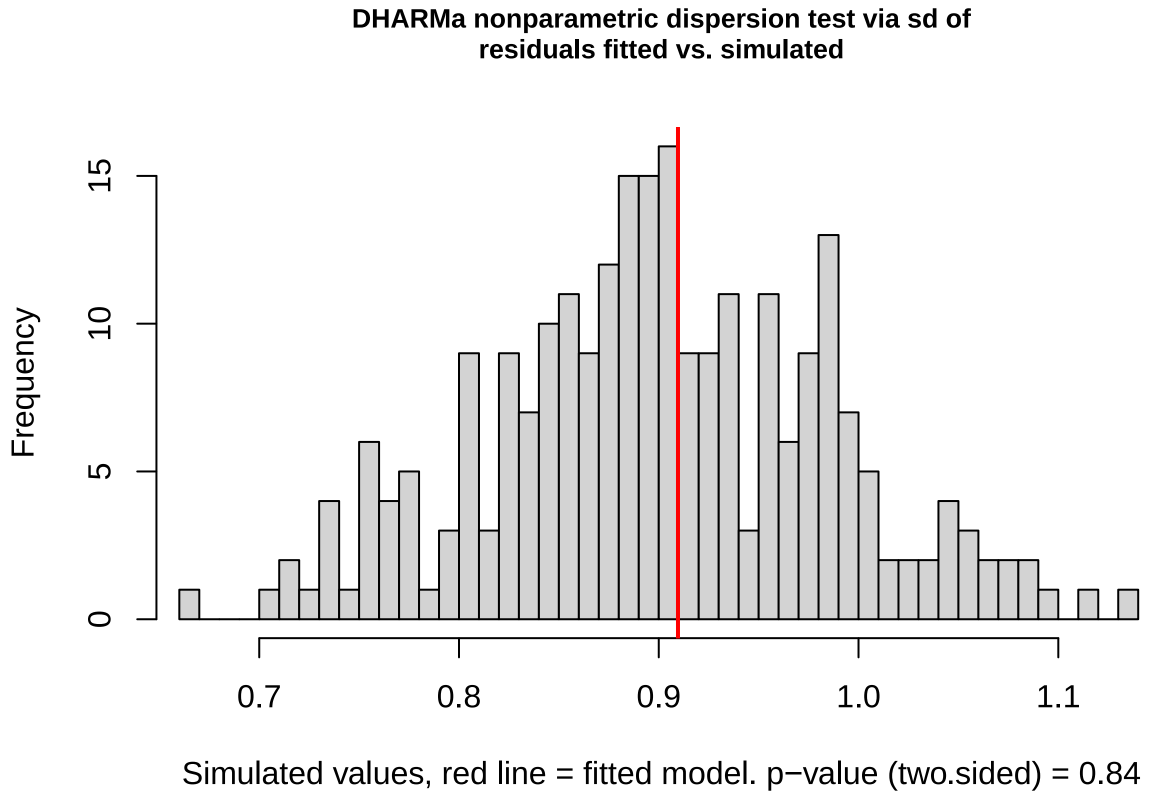


```
##
##  DHARMa nonparametric dispersion test via sd of residuals fitted vs.
##  simulated
##
## data:  simulationOutput
## dispersion = 1.0111, p-value = 0.84
## alternative hypothesis: two.sided
```

```
check_collinearity(finalPinyonMod)
```

```
## # Check for Multicollinearity
##
## Low Correlation
##
##                                                                                                     Term
##                                                                                              crown_class
##                                                          scale(propBA_Loss, scale = sd(propBA_Loss) * 2)
##                                              scale(meanCWD_1991_2020, scale = sd(meanCWD_1991_2020) * 2)
##                                                                    scale(DRC_cm, scale = sd(DRC_cm) * 2)
##  scale(meanCWD_1991_2020, scale = sd(meanCWD_1991_2020) * 2):scale(DRC_cm, scale = sd(DRC_cm) * 2)
##   VIF    VIF 95% CI Increased SE Tolerance Tolerance 95% CI
##  1.02 [1.00,  3.22]         1.01      0.98     [0.31, 1.00]
##  1.01 [1.00, 25.27]         1.01      0.99     [0.04, 1.00]
##  1.04 [1.00,  1.68]         1.02      0.96     [0.60, 1.00]
##  1.10 [1.03,  1.33]         1.05      0.91     [0.75, 0.97]
```

```
##  1.13 [1.05,  1.34]         1.06      0.89     [0.75, 0.95]
```

```
# Spatial autocorrelation
  # Join with plot coordinates
pinyon <- pinyon %>%
  mutate(Tran_Sect = paste(Site, Section, sep = "-")) %>%
  left_join(plots, by = "Tran_Sect") %>%
  st_as_sf()
pinyon$X <- st_coordinates(pinyon)[,1]; pinyon$Y <- st_coordinates(pinyon)[,2]
  # Aggregate residuals to plot-level for testing
refit <- recalculateResiduals(resd, group = as.character(pinyon$Tran_Sect))
refit$X <- aggregate(pinyon$X, list(as.factor(as.character(pinyon$Tran_Sect))), mean)$x
refit$Y <- aggregate(pinyon$Y, list(as.factor(as.character(pinyon$Tran_Sect))), mean)$x
  # Spline correlogram for all points, aggregated to plot-level
plot(spline.correlog(refit$X, refit$Y, refit$scaledResiduals,
                     latlon = F, resamp = 200, quiet = T)) ## Testing at max distance. Roughly 30,000 m
```

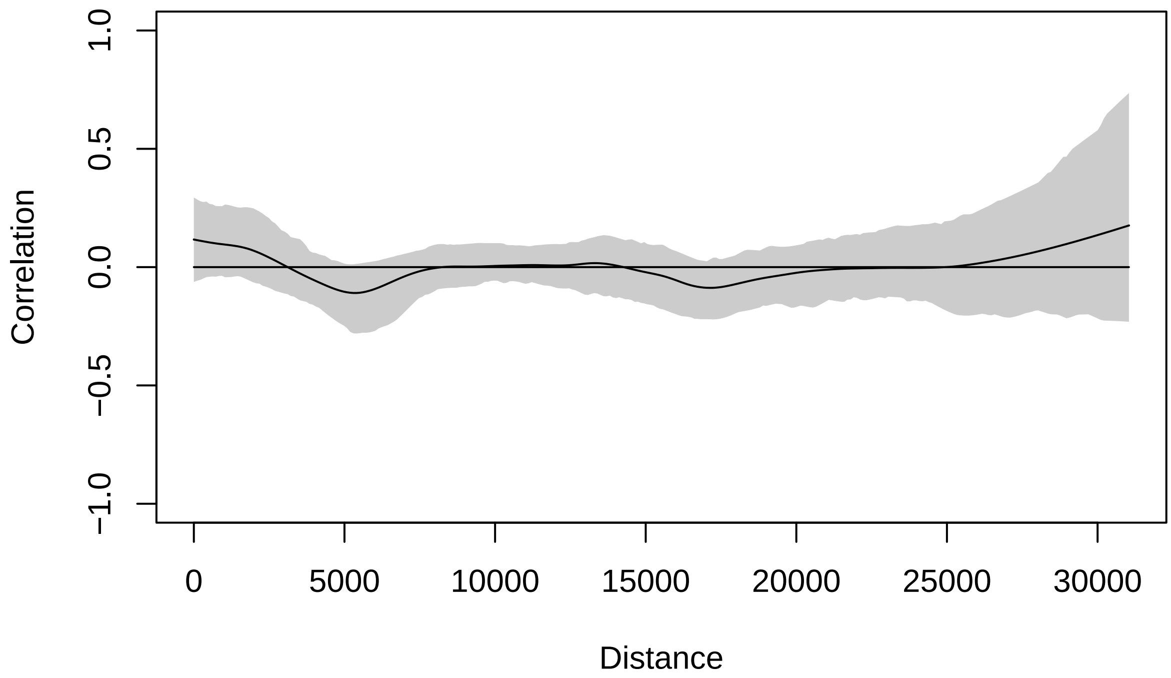


```
testSpatialAutocorrelation(refit, refit$X, refit$Y) ## Significant, but not particularly problematic au
```

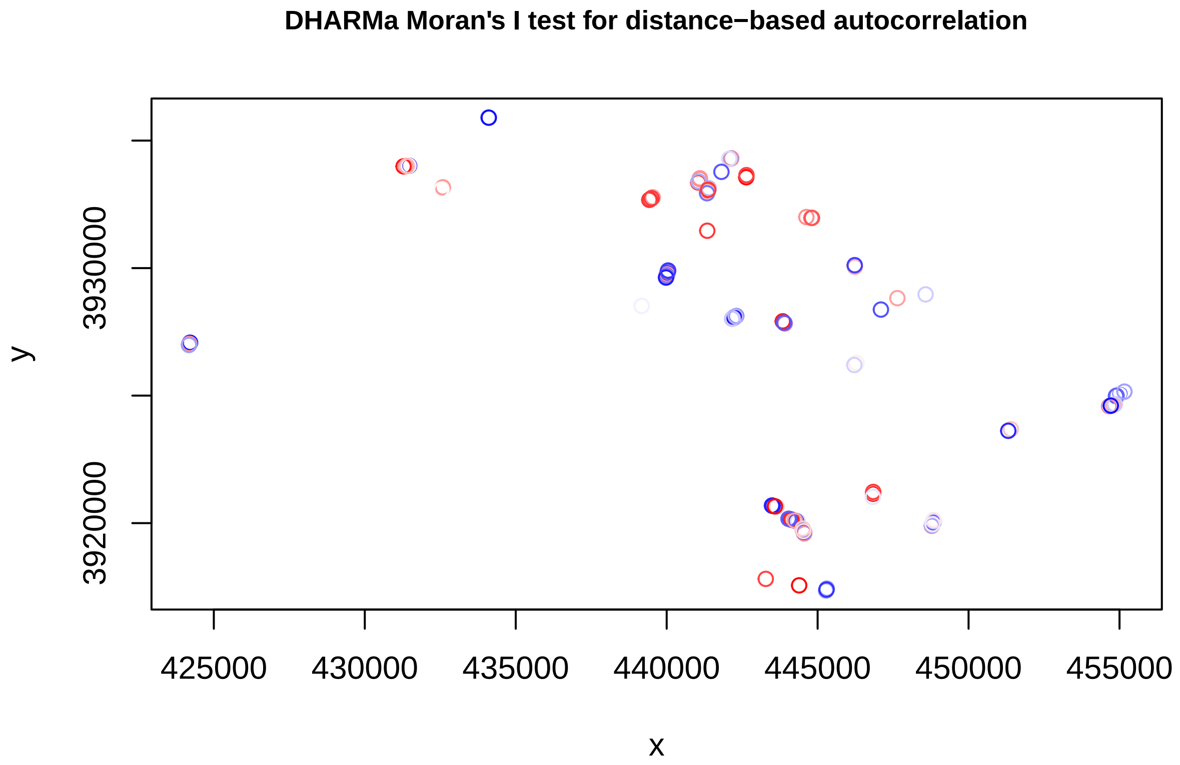


```
##
##  DHARMa Moran's I test for distance-based autocorrelation
##
## data:  refit
## observed = 0.1420898, expected = -0.0076336, sd = 0.0520386, p-value =
## 0.004013
## alternative hypothesis: Distance-based autocorrelation
```

```r
## Testing model goodness of fit
auc(pinyon$Survived, predict(finalPinyonMod, newdata = pinyon,
                             re.form = NA, type = "response"))
```

```
## Setting levels: control = 0, case = 1

## Setting direction: controls < cases

## Area under the curve: 0.699
```

```r
auc(pinyon$Survived, predict(finalPinyonMod, newdata = pinyon,
                             re.form = NULL, type = "response"))
```

```
## Setting levels: control = 0, case = 1
## Setting direction: controls < cases
```

```
## Area under the curve: 0.8611
```

```r
r.squaredGLMM(finalPinyonMod)
```

```
##                    R2m       R2c
## theoretical 0.1427088 0.2780362
## delta       0.1484945 0.2893082
```

So, model #1 looks pretty good, all things considered. Some minimal but likely unproblematic spatial autocorrelation. Residuals look great, and no evidence of collinearity based on VIFs.

4.2 Model #2: Survival of juniper

Like model #1, we go through the steps of fitting the full model. Note that we first test a simple non-linear effect of Average CWD (a natural spline with two degrees of freedom), with the idea that some intermediate value of climate may represent optimal conditions for survival. Here again, the model seems to suggest overfitting at the tails, so we stuck with a simple linear term.

```r
## First, does non-linear term for CWD make sense?
cwdMod1 <- glmmTMB(Survived ~ scale(meanCWD_1991_2020,
                                    scale = sd(meanCWD_1991_2020)*2) +
                     (1|Site/Section), data = juniper,
                   family = binomial(link = "cloglog"))
cwdMod2 <- glmmTMB(Survived ~ ns(scale(meanCWD_1991_2020,
                                       scale = sd(meanCWD_1991_2020)*2), df = 2) +
                     (1|Site/Section), data = juniper,
                   family = binomial(link = "cloglog"))
AICc(cwdMod1, cwdMod2) ## Lower AICc in more complex model
```

```
##         df     AICc
## cwdMod1  4 596.7117
## cwdMod2  5 593.9727
```

```r
predict(cwdMod1, newdata = data.frame(meanCWD_1991_2020 = c(0,100,200,300,400,500,600),
                                      Site = "999", Section = "999"),
        allow.new.levels = T, type = "response")
```

```
## [1] 0.7557758 0.8246861 0.8835911 0.9298001 0.9624135 0.9826250 0.9933010
```

```r
predict(cwdMod2, newdata = data.frame(meanCWD_1991_2020 = c(0,100,200,300,400,500,600),
                                      Site = "999", Section = "999"),
        allow.new.levels = T, type = "response")
```

```
## [1] 0.9005811 0.8803717 0.8700269 0.8741487 0.8940062 0.9240903 0.9556912
```

```r
# We should use the simpler model, lower survival in intermediate climates isn't really logical

## Fit full juniper model
juniperSub <- juniper %>% filter(!is.na(wood)) ## Removing rows without cover/soils data
```

```r
juniperMod <- glmmTMB(Survived ~ scale(DRC_cm, scale = sd(DRC_cm)*2)*
                        scale(liveBA_sqM_ha, scale = sd(liveBA_sqM_ha)*2) +
                        scale(propBA_Loss, scale = sd(propBA_Loss)*2)*
                        scale(liveBA_sqM_ha, scale = sd(liveBA_sqM_ha)*2) +
                        scale(meanCWD_1991_2020, scale = sd(meanCWD_1991_2020)*2)*
                        scale(DRC_cm, scale = sd(DRC_cm)*2) +
                        crown_class + ## Removing CC by DRC interaction because it leads to singular mod
                        scale(wood, scale = sd(wood)*2)+
                        scale(OrganicMatter_Percent, scale = sd(OrganicMatter_Percent)*2)+
                        (1|Site/Section),
                      data = juniperSub, family = binomial(link = "cloglog"),
                      na.action = "na.fail")
```

Next, we perform model selection using this fitted model object

```r
## Model selection using all subsets
#juniperModSelection <- dredge(juniperMod)

## Based on model selection, the top model follows the form "Survived ~ DRC_cm*liveBA_sqM_ha + meanCWD_
finalJuniperMod <- glmmTMB(Survived ~ scale(DRC_cm, scale = sd(DRC_cm)*2)*
                             scale(liveBA_sqM_ha, scale = sd(liveBA_sqM_ha)*2) +
                             scale(meanCWD_1991_2020, scale = sd(meanCWD_1991_2020)*2)*
                             scale(DRC_cm, scale = sd(DRC_cm)*2) +
                             crown_class +
                             scale(OrganicMatter_Percent, scale = sd(OrganicMatter_Percent)*2) +
                             (1|Site/Section),
                           data = juniper, family = binomial(link = "cloglog")) ## Adding back in the r
```

Using the final model, we test a few residual diagnostics, autocorrelation, and quantify goodness of fit using the MuMIn::r.squaredGLMM ar pROC::AUC functions.

```r
## Testing assumptions. These also look pretty decent

# Overall residuals and collinearity
resd <- simulateResiduals(finalJuniperMod)
plot(resd)
```

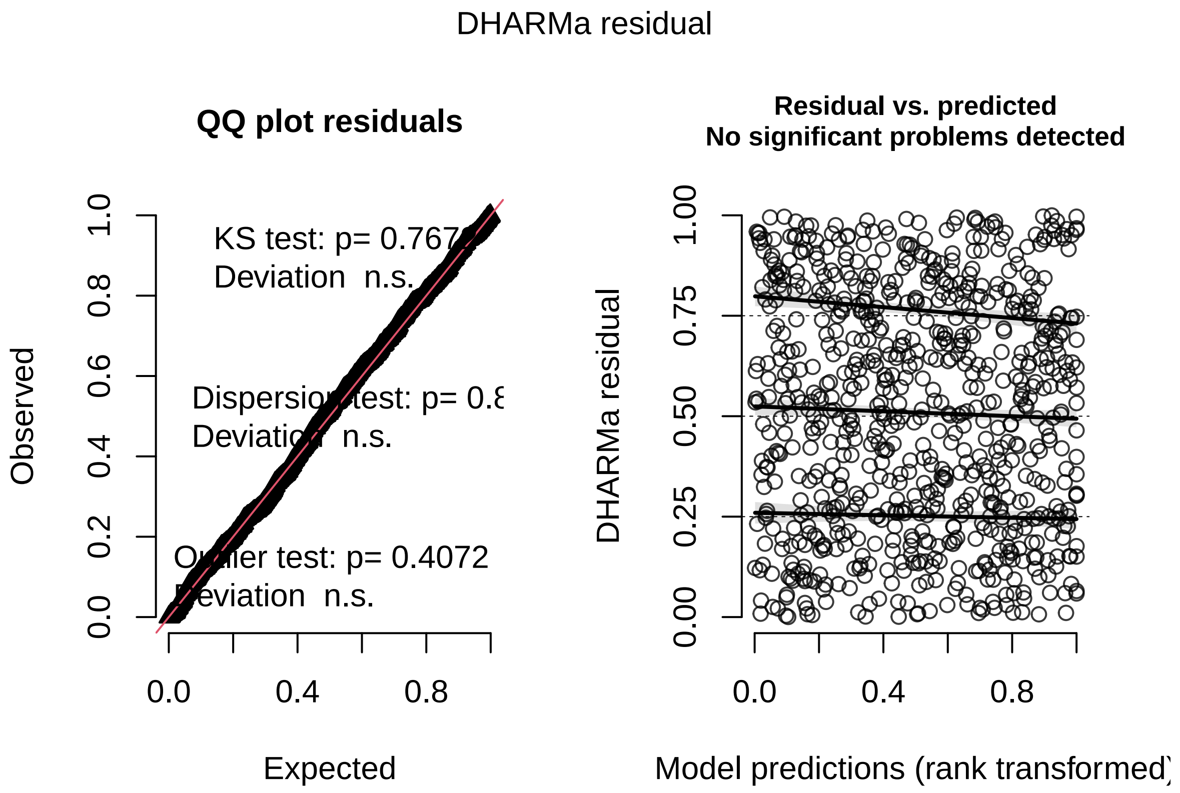


```
testDispersion(resd)
```

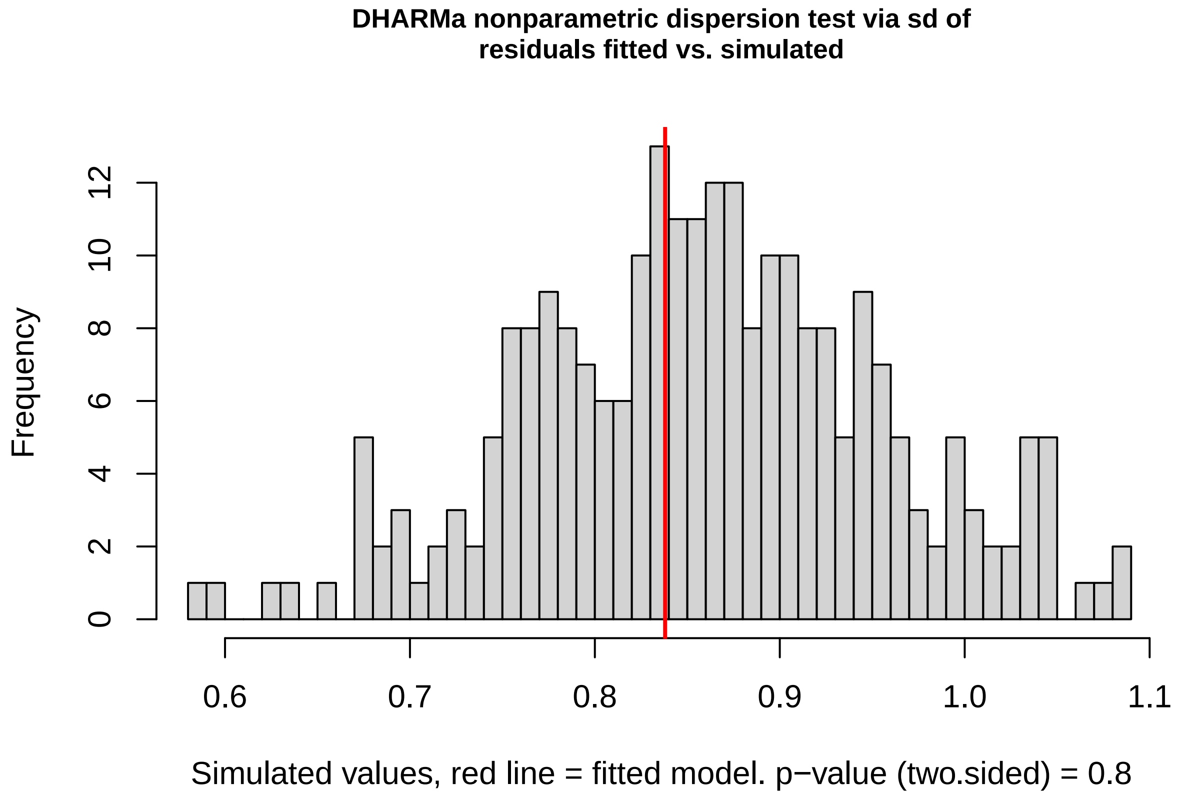


```
##
##  DHARMa nonparametric dispersion test via sd of residuals fitted vs.
##  simulated
##
## data:  simulationOutput
## dispersion = 0.97431, p-value = 0.8
## alternative hypothesis: two.sided
```

```
check_collinearity(finalJuniperMod)
```

```
## # Check for Multicollinearity
##
## Low Correlation
##
##                                                                                 Term
##                                                   scale(DRC_cm, scale = sd(DRC_cm) * 2)
##                                       scale(liveBA_sqM_ha, scale = sd(liveBA_sqM_ha) * 2)
##                               scale(meanCWD_1991_2020, scale = sd(meanCWD_1991_2020) * 2)
##                                                                          crown_class
##                       scale(OrganicMatter_Percent, scale = sd(OrganicMatter_Percent) * 2)
##          scale(DRC_cm, scale = sd(DRC_cm) * 2):scale(liveBA_sqM_ha, scale = sd(liveBA_sqM_ha) * 2)
##  scale(DRC_cm, scale = sd(DRC_cm) * 2):scale(meanCWD_1991_2020, scale = sd(meanCWD_1991_2020) * 2)
##   VIF   VIF 95% CI Increased SE Tolerance Tolerance 95% CI
##  2.11 [1.90, 2.36]         1.45      0.47     [0.42, 0.53]
##  1.34 [1.24, 1.48]         1.16      0.74     [0.67, 0.80]
```

```
##  1.18 [1.11, 1.31]         1.09      0.85     [0.77, 0.90]
##  1.16 [1.09, 1.29]         1.08      0.86     [0.78, 0.92]
##  1.16 [1.09, 1.29]         1.08      0.86     [0.78, 0.92]
##  1.69 [1.54, 1.88]         1.30      0.59     [0.53, 0.65]
##  1.24 [1.15, 1.37]         1.11      0.81     [0.73, 0.87]
```

```
# Spatial autocorrelation
  # Join with plot coordinates
juniper <- juniper %>%
  mutate(Tran_Sect = paste(Site, Section, sep = "-")) %>%
  left_join(plots, by = "Tran_Sect") %>%
  st_as_sf()
juniper$X <- st_coordinates(juniper)[,1]; juniper$Y <- st_coordinates(juniper)[,2]
  # Aggregate residuals to plot-level for testing
refit <- recalculateResiduals(resd, group = as.character(juniper$Tran_Sect))
refit$X <- aggregate(juniper$X, list(as.factor(as.character(juniper$Tran_Sect))), mean)$x
refit$Y <- aggregate(juniper$Y, list(as.factor(as.character(juniper$Tran_Sect))), mean)$x
  # Spline correlogram for all points, aggregated to plot-level
plot(spline.correlog(refit$X, refit$Y, refit$scaledResiduals,
                     latlon = F, resamp = 200, quiet = T)) ## Testing at max distance. Roughly 30,000 m
```

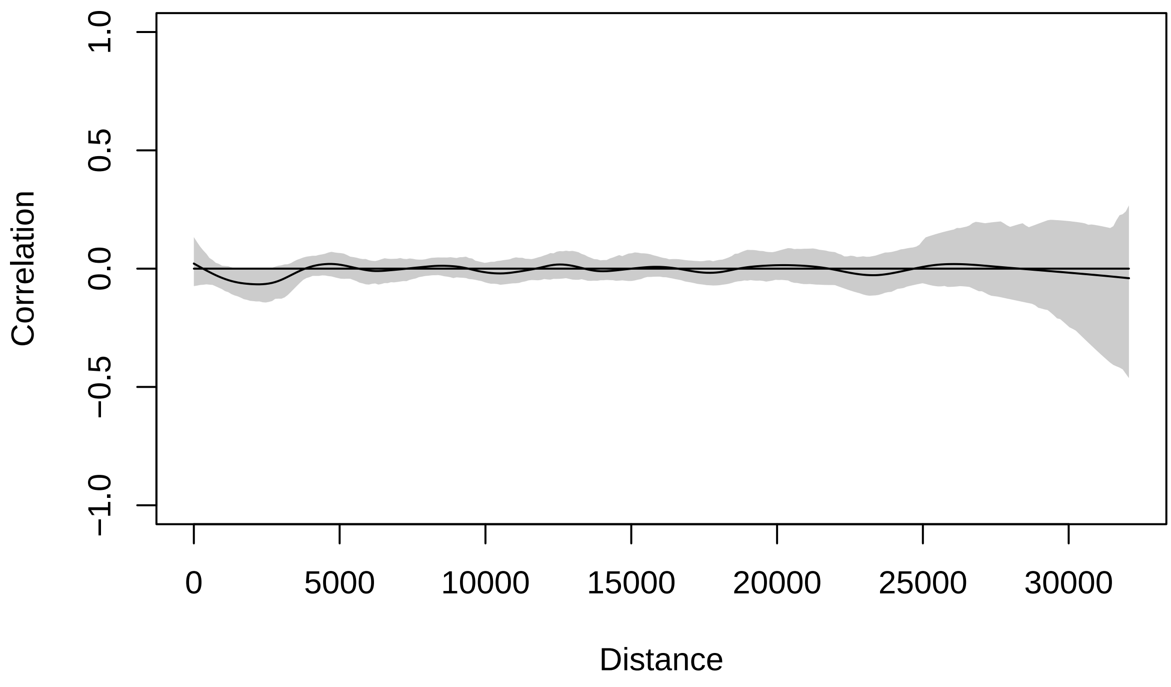


```
testSpatialAutocorrelation(refit, refit$X, refit$Y) ## Pretty much zero
```

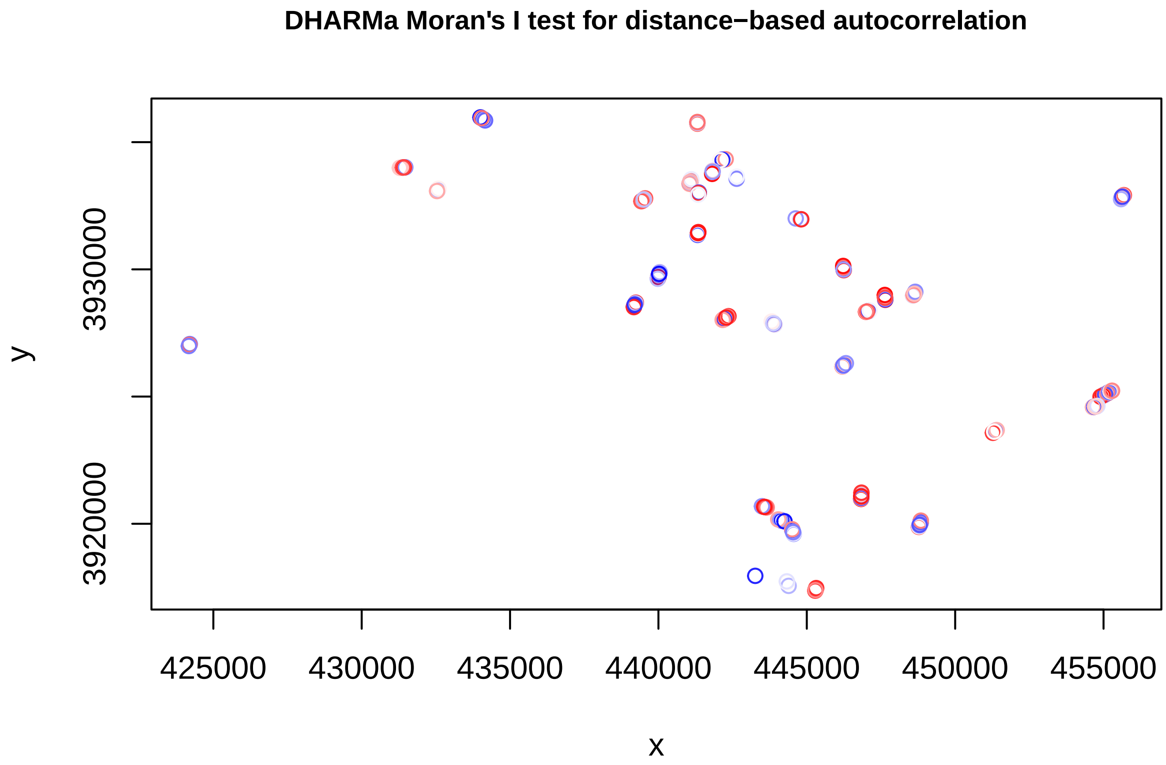


```
##
##  DHARMa Moran's I test for distance-based autocorrelation
##
## data:  refit
## observed = 0.0232180, expected = -0.0042373, sd = 0.0329548, p-value =
## 0.4048
## alternative hypothesis: Distance-based autocorrelation
```

```r
## Checking goodness of fit
auc(juniper$Survived, predict(finalJuniperMod, newdata = juniper,
                              re.form = NA, type = "response"))
```

```
## Setting levels: control = 0, case = 1
```

```
## Setting direction: controls < cases
```

```
## Area under the curve: 0.7806
```

```r
auc(juniper$Survived, predict(finalJuniperMod, newdata = juniper,
                              re.form = NULL, type = "response"))
```

```
## Setting levels: control = 0, case = 1
## Setting direction: controls < cases
```

```
## Area under the curve: 0.9487
```

```r
r.squaredGLMM(finalJuniperMod)
```

```
##                   R2m       R2c
## theoretical 0.3181037 0.5357594
## delta       0.3166388 0.5332921
```

Again, no real concerns here. The model assumptions seem to be met.

4.3 Model #3: Small juniper survival

This model is specifically focused on the survival of small juniper, as it relates to individual size and plot-scale AM colonization. Because we only processed samples for AM colonization for a subset of plots (n = 26), this model includes only two possible covariates and one interaction. The analyses are still completed at the tree scale, because DRC varies at this level.

So, first, let's fit a full model with the two terms and a two-way interaction

```r
## Fit full model
sizeByAMMod <- glmmTMB(Survived  ~ scale(DRC_cm, scale = sd(DRC_cm)*2)*
                         scale(Total_AM_perc, scale = sd(Total_AM_perc)*2) +
                         (1|Site/Section),
                       data = mycoSurvivalTrees, family = binomial(link = "cloglog"),
                       na.action = "na.fail")
```

Then perform model selection using all subsets, as before

```r
## Model selection using all subsets
#smallJuniperModSelection <- dredge(sizeByAMMod)

## Based on model selection, the top model follows the form "Survived ~ DRC_cm + Total AM + (1|Site/Sec
sizePlusAMMod <- glmmTMB(Survived  ~ scale(DRC_cm, scale = sd(DRC_cm)*2)+
                           scale(Total_AM_perc, scale = sd(Total_AM_perc)*2) +
                           (1|Site/Section),
                       data = mycoSurvivalTrees, family = binomial(link = "cloglog"))
```

Using the final model, we test a few residual diagnostics, autocorrelation, and quantify goodness of fit using the MuMIn::r.squaredGLMM ar pROC::AUC functions.

```r
## Testing assumptions. These also look pretty decent

# Overall residual distributions and collinearity
resd <- simulateResiduals(sizePlusAMMod)
plot(resd) ## Looks great
```

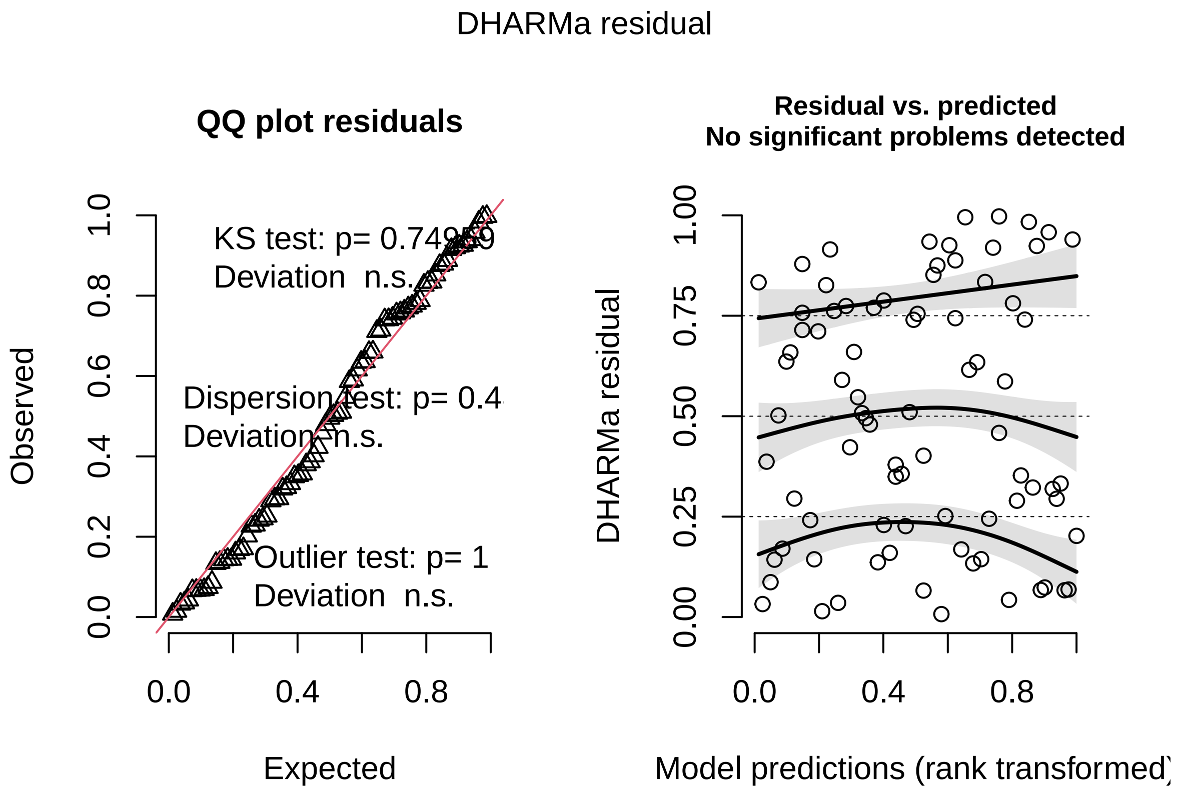


```
testDispersion(resd) ## No problems there either
```

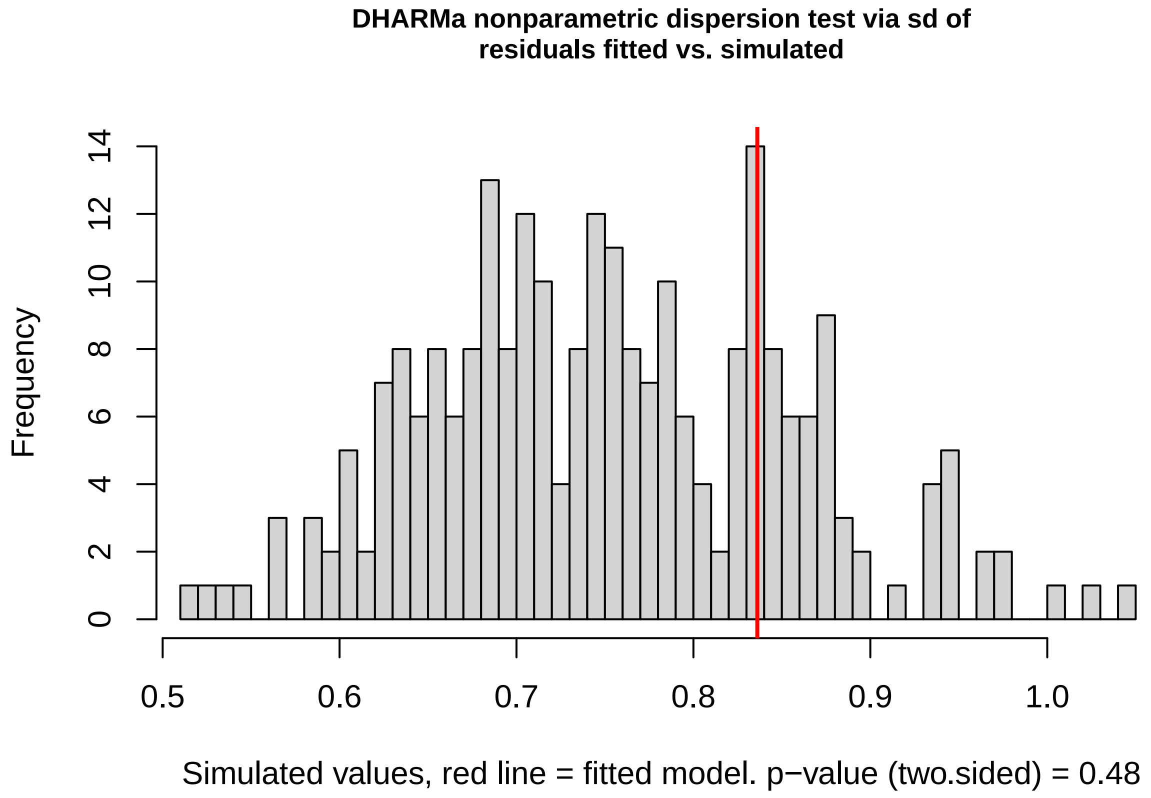


```
##
##  DHARMa nonparametric dispersion test via sd of residuals fitted vs.
##  simulated
##
## data:  simulationOutput
## dispersion = 1.1109, p-value = 0.48
## alternative hypothesis: two.sided
```

```
check_collinearity(sizePlusAMMod) ## Likewise
```

```
## # Check for Multicollinearity
##
## Low Correlation
##
##                                                   Term  VIF   VIF 95% CI
##                 scale(DRC_cm, scale = sd(DRC_cm) * 2) 1.57 [1.27, 2.23]
##  scale(Total_AM_perc, scale = sd(Total_AM_perc) * 2) 1.57 [1.27, 2.23]
##  Increased SE Tolerance Tolerance 95% CI
##         1.25      0.64     [0.45, 0.79]
##         1.25      0.64     [0.45, 0.79]
```

```
# Spatial autocorrelation
  # Join with plot coordinates
mycoSurvivalTrees <- mycoSurvivalTrees %>%
  mutate(Tran_Sect = paste(Site, Section, sep = "-")) %>%
```

```
  left_join(plots, by = "Tran_Sect") %>%
  st_as_sf()
mycoSurvivalTrees$X <- st_coordinates(mycoSurvivalTrees)[,1]
mycoSurvivalTrees$Y <- st_coordinates(mycoSurvivalTrees)[,2]
  # Aggregate residuals to plot-level for testing
refit <- recalculateResiduals(resd, group = as.character(mycoSurvivalTrees$Tran_Sect))
refit$X <- aggregate(mycoSurvivalTrees$X,
                     list(as.factor(as.character(mycoSurvivalTrees$Tran_Sect))), mean)$x
refit$Y <- aggregate(mycoSurvivalTrees$Y,
                     list(as.factor(as.character(mycoSurvivalTrees$Tran_Sect))), mean)$x
  # Spline correlogram for all points, aggregated to plot-level
plot(spline.correlog(refit$X, refit$Y, refit$scaledResiduals,
                     latlon = F, resamp = 200, quiet = T)) ## Testing at max distance. Roughly 30,000 m
```

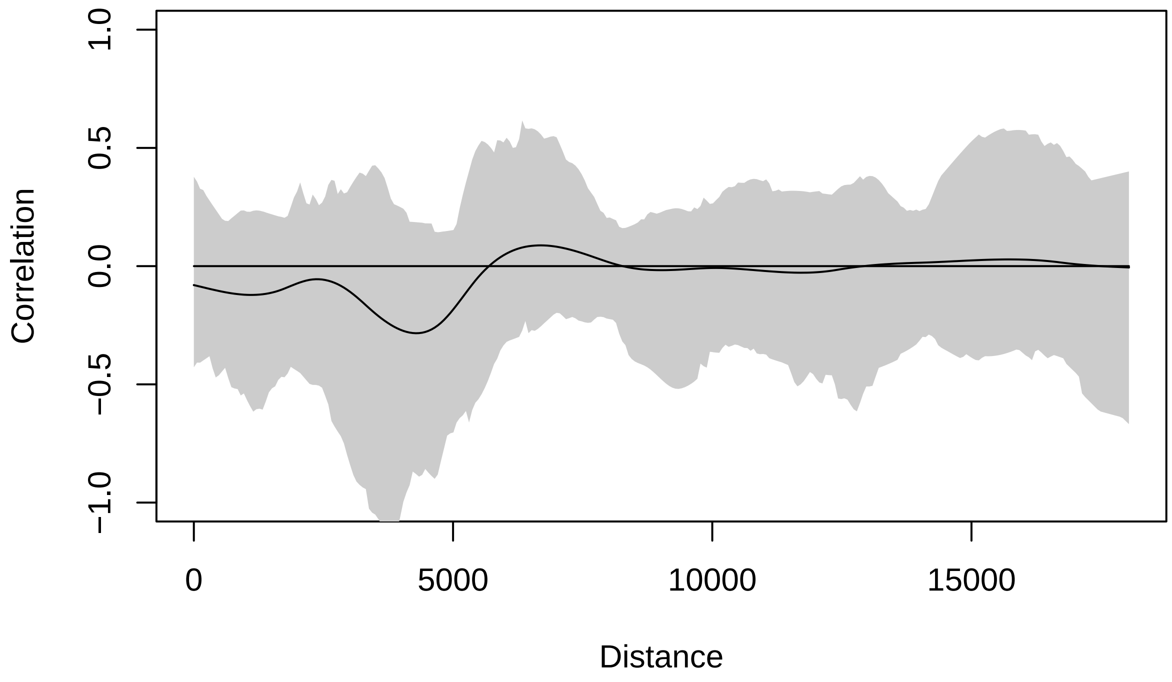


```
testSpatialAutocorrelation(refit, refit$X, refit$Y) ## Basically 0. No problems here either
```

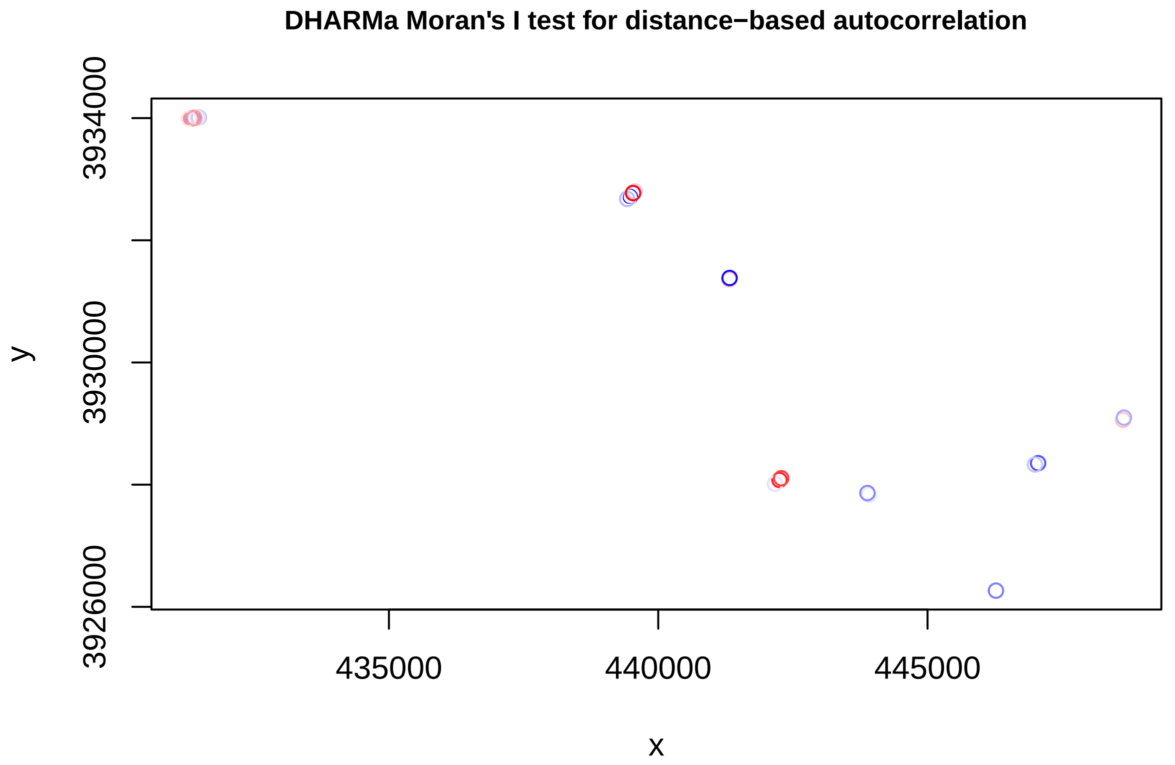


```
##
##  DHARMa Moran's I test for distance-based autocorrelation
##
## data:  refit
## observed = -0.050272, expected = -0.040000, sd = 0.170449, p-value =
## 0.9519
## alternative hypothesis: Distance-based autocorrelation
```

```
## View model goodness of fit
auc(mycoSurvivalTrees$Survived, predict(sizePlusAMMod, newdata = mycoSurvivalTrees,
                                        re.form = NA, type = "response"))
```

```
## Setting levels: control = 0, case = 1
```

```
## Setting direction: controls < cases
```

```
## Area under the curve: 0.7566
```

```
auc(mycoSurvivalTrees$Survived, predict(sizePlusAMMod, newdata = mycoSurvivalTrees,
                                        re.form = NULL, type = "response"))
```

```
## Setting levels: control = 0, case = 1
## Setting direction: controls < cases
```

```
## Area under the curve: 0.9009
```

```r
r.squaredGLMM(sizePlusAMMod)
```

```
##                   R2m       R2c
## theoretical 0.3859042 0.5587154
## delta       0.3854818 0.5581039
```

This one looks even better than the other two, despite the smaller sample size. So, we conclude based on the tests on each model in the above subsections that these models are appropriate for inference with our data given their current structure.

5 Using fitted model objects to make predictions of mortality risk

This section is our final analysis for the manuscript, and shows changes in tree-scale and site-scale mortality probability from 2014 to 2023, based on observed changes in basal area, size structure, individual live crown, and species composition. To do this, we use final versions of Models #1 and #2 above to predict survival probability for each live tree present in 2014, and each live tree present in 2022/2023. Importantly, this includes additional individuals not used to develop the models - for example new recruitment that is present in 2022/2023.

There are a few steps involved in this. First, we read in the full tree list (i.e., one row per tree and remeasurement period), and other covariates (e.g., soils, climate) that had not yet been merged with the full dataset

```r
### Reading in and processing some datasets for landscape analysis of changes in mortality risk

## Full tree list, with one row per individual and measurement period (i.e., long form)
trees <- read_csv(here("Data", "Processed", "AllTrees_corrected.csv")) %>%
  filter(((!is.na(DRC_cm) & DRC_cm > 0.5)|
            (!is.na(Height_cm) & Height_cm > 5))) ##  Like other analyses, we are only including those

## Soils data - for tree-level predictions
soils <- read_csv(here("Data", "Processed", "SoilData.csv"))

## Getting plot locations to visualize changes in mortality probability and get fire info
plots <- st_read(here("Data", "Processed", "PlotBoundaries.shp"),
                 quiet = T) %>%
  rename("Tran_Sect" = Trn_Sct) %>%
  dplyr::select(Tran_Sect, frstFrY) %>%
  st_drop_geometry() %>%
  right_join(st_read(here("Data", "Spatial", "SiteLocations", "plotCenters_5_11_22_WGS84.shp"), quiet =
             by = "Tran_Sect")

## Map of average cwd for basemap/background
avgCwd <- rast(here("Data", "Spatial", "WaterBalance", "AverageCWD_1991-2020.tif"))
```

Next process these data to get them in a single data frame for prediction. This involves a bit of wrangling to get Average CWD and live BA for each period

```r
## Get 30-year average CWD
plotsSpatial <- st_read(here("Data", "Processed", "PlotBoundaries.shp"))
```

```
## Reading layer 'PlotBoundaries' from data source
##   'C:\Users\kcr92\OneDrive - Northern Arizona University\ERI Work\Manuscripts\Rodman_PJ_Mortality\Da
##   using driver 'ESRI Shapefile'
## Simple feature collection with 463 features and 3 fields
## Geometry type: MULTIPOLYGON
## Dimension:     XY
## Bounding box:  xmin: -111.857 ymin: 35.39001 xmax: -111.4273 ymax: 35.56529
## Geodetic CRS:  WGS 84
```

```r
cwdExtract <- exactextractr::exact_extract(avgCwd, plotsSpatial, "mean")
```

```
##   |                                                                                |
```

```r
climateSummarized <- cbind(plotsSpatial, cwdExtract)
climateSummarized <- climateSummarized %>%
  rename("meanCWD_1991_2020" = cwdExtract,
         "Tran_Sect" = Trn_Sct) %>%
  dplyr::select(Tran_Sect, meanCWD_1991_2020) %>%
  st_drop_geometry()

## Join it with tree data by plot ID
treesWithSpatial <- trees %>%
  mutate(Tran_Sect = paste(Site, Section, sep = "-")) %>%
  left_join(plots, by = "Tran_Sect") %>%
  left_join(climateSummarized, by = "Tran_Sect") %>%
  st_as_sf()

## Get live BA by plot and year, in Sq. m/ha
liveBA <- trees %>%
  filter(Status == "L") %>%
  mutate(Tran_Sect = paste(Site, Section, sep = "-")) %>%
  group_by(Tran_Sect, Year) %>%
  summarise(liveBA_sqM_ha = sum((DRC_cm^2)*0.00007854, na.rm = T)/0.01)

## Get just the initial BA ca. 2000
initialBA <- trees %>%
  filter(Year < 2002 & Status == "L") %>%
  # And group by plot ID and year to get live BA
  mutate(Tran_Sect = paste(Site, Section, sep = "-")) %>%
  group_by(Tran_Sect) %>%
  mutate(BA_sqM = (DRC_cm^2)*0.00007854) %>%
  summarise(initLiveBA_sqM_ha = sum(BA_sqM, na.rm = T)/0.01)

## Get the difference between initial and 2014 BA as metric of drought-caused loss
liveBA <- liveBA %>%
  right_join(initialBA, by = c("Tran_Sect"))

## Next, add any necessary variables to predict mortality probability
treesForPrediction <- treesWithSpatial %>%
```

```r
  left_join(liveBA, by = c("Tran_Sect", "Year")) %>%
  left_join(soils, by = c("Tran_Sect")) %>%
  filter(Species == "P"|Species == "J") %>%
  filter((Year == 2014|Year > 2021) & Status == "L") %>% ## Get rid of the small ones
  mutate(Year = ifelse(Year == 2014, "2014", "2022-2023"),
         propBA_Loss = 1-(liveBA_sqM_ha/(initLiveBA_sqM_ha))) %>%
  mutate(propBA_Loss = ifelse(propBA_Loss < 0, 0, propBA_Loss)) %>%
  filter(is.na(frstFrY)|frstFrY < 2014) %>%
  select(c(Year, Species, DRC_cm, liveBA_sqM_ha, propBA_Loss, meanCWD_1991_2020,
           OrganicMatter_Percent, crown_class, Site)) %>%
  na.omit()

## A quick look at this data frame that has been formatted for prediction. One row for each live tree p
head(treesForPrediction)
```

```
## Simple feature collection with 6 features and 9 fields
## Geometry type: POINT
## Dimension:     XY
## Bounding box:  xmin: -111.6705 ymin: 35.49906 xmax: -111.67 ymax: 35.4999
## Geodetic CRS:  WGS 84
## # A tibble: 6 x 10
##   Year      Species DRC_cm liveBA_sqM_ha propBA_Loss meanCWD_1991_2020
##   <chr>     <chr>    <dbl>         <dbl>       <dbl>             <dbl>
## 1 2014      J         30           21.3        0.399              132.
## 2 2022-2023 J         30            7.07       0.800              132.
## 3 2014      J         42.5         21.3        0.399              132.
## 4 2014      J         46           26.1        0.269              120.
## 5 2022-2023 J         46           26.1        0.269              120.
## 6 2014      J         34.7         26.1        0.269              120.
## # i 4 more variables: OrganicMatter_Percent <dbl>, crown_class <dbl>,
## #   Site <dbl>, geometry <POINT [°]>
```

Now, we take this data frame and use fitted model objects to predict interval-level survival probability (i.e., not annualized). Predictions are made on the link scale to allow us to incorporate prediction at that scale before transferring back to the response scale (i.e., interval-level survival probability). The tables below show the overall predictions of survival probability +- 1SE of prediction, in each time period

```r
### Now, predicting on these trees using fitted models - conditional mean +- 1 SE

## For pinyon
pinyonPredictions <- predict(finalPinyonMod, newdata = treesForPrediction,
                             type = "link", allow.new.levels = T, re.form = NA,
                             se = T) ## Set random effects to 0, so that we don't influence predictions

## For juniper
juniperPredictions <- predict(finalJuniperMod, newdata = treesForPrediction,
                              type = "link", allow.new.levels = T, re.form = NA,
                              se = T) ## Set random effects to 0

## And assign each tree to one of the predictions above based on species
treesForPrediction$predictedProb <- finalJuniperMod$modelInfo$family$linkinv(
  ifelse(treesForPrediction$Species == "P",
         pinyonPredictions$fit, juniperPredictions$fit))
```

```r
## Finally, define upper or lower prediction intervals as conditional mean +- 1SE
treesForPrediction$upper <- finalJuniperMod$modelInfo$family$linkinv(
  ifelse(treesForPrediction$Species == "P", pinyonPredictions$fit, juniperPredictions$fit) +
    ifelse(treesForPrediction$Species == "P", pinyonPredictions$se.fit, juniperPredictions$se.fit))
treesForPrediction$lower <- finalJuniperMod$modelInfo$family$linkinv(
  ifelse(treesForPrediction$Species == "P", pinyonPredictions$fit, juniperPredictions$fit) -
    ifelse(treesForPrediction$Species == "P", pinyonPredictions$se.fit, juniperPredictions$se.fit))
treesForPrediction$Species <-
  factor(ifelse(treesForPrediction$Species == "P", "Pinus edulis", "Juniperus monosperma"),
         levels = c("Pinus edulis", "Juniperus monosperma"))

## And aggregating by transect, Species, and year to get survey period-specific survival probability
survivalSppProbs <- treesForPrediction %>%
  group_by(Site, Species, Year) %>%
  summarise(meanSurvival = mean(predictedProb, na.rm = T),
            meanUpper = mean(upper, na.rm = T), meanLower = mean(lower, na.rm = T)) %>%
  ungroup()
```

```
## `summarise()` has grouped output by 'Site', 'Species'. You can override using
## the `.groups` argument.
```

```r
# Fixing duplicated coords
coords <- st_coordinates(st_centroid(survivalSppProbs))
survivalSppProbs$Long <- coords[,1]
survivalSppProbs$Lat <- coords[,2]
survivalSppProbs <- survivalSppProbs %>%
  group_by(Site, Species, Year, meanSurvival) %>%
  st_drop_geometry() %>%
  st_as_sf(coords = c("Long", "Lat"), crs = "EPSG:4326")
survivalSppProbs$Species <- factor(survivalSppProbs$Species,
                                   levels = c("Pinus edulis", "Juniperus monosperma"))
# Summarizing mean and SE
(meanSppValues <- treesForPrediction %>%
    st_drop_geometry() %>%
    group_by(Year, Species) %>%
    summarise(seSurvival = sd(predictedProb, na.rm = T)/sqrt(n()),
              predictedProb = mean(predictedProb, na.rm = T),
              xmin = predictedProb-seSurvival,
              xmax = predictedProb+seSurvival))
```

```
## `summarise()` has grouped output by 'Year'. You can override using the
## `.groups` argument.

## # A tibble: 4 x 6
## # Groups:   Year [2]
##   Year      Species              seSurvival predictedProb  xmin  xmax
##   <chr>     <fct>                     <dbl>         <dbl> <dbl> <dbl>
## 1 2014      Pinus edulis            0.00720         0.743 0.735 0.750
## 2 2014      Juniperus monosperma    0.00534         0.889 0.884 0.895
## 3 2022-2023 Pinus edulis            0.00984         0.727 0.718 0.737
## 4 2022-2023 Juniperus monosperma    0.00582         0.869 0.863 0.874
```

```
meanSppValues$Species <- factor(meanSppValues$Species,
                                levels = c("Pinus edulis", "Juniperus monosperma"))
```

Finally, plot some of these results

```
## Making plots of mapped changes in survival probability - separately by species
  # Tree-level
a <- ggplot(NULL, aes(x = predictedProb, group = Year, color = Species, linetype = Year)) +
    facet_grid(.~Species) +
    geom_vline(data = meanSppValues, aes(xintercept = predictedProb, color = Species, linetype = Year))
    geom_density(data = treesForPrediction, alpha = 0, linewidth = 1) +
    scale_color_manual(values = c("#f1a340", "#998ec3")) +
    theme(legend.position = "top") +
    theme_bw() + theme(strip.background = element_blank()) +
    xlab("Tree-Level Survival Probability") + ylab("Density") +
    theme(legend.position = "Top")

# Landscape-level
(survivalProbs2 <- survivalSppProbs %>%
  group_by(Site, Species) %>%
  arrange(Year, .by_group = T) %>%
  mutate(changeInSurvival = meanSurvival - lag(meanSurvival),
         decline = ifelse(changeInSurvival < 0, 1, 0)) %>%
  filter(Year == "2022-2023"))
```

```
## Simple feature collection with 65 features and 8 fields
## Geometry type: POINT
## Dimension:     XY
## Bounding box:  xmin: -111.8357 ymin: 35.3984 xmax: -111.4894 ymax: 35.56468
## Geodetic CRS:  WGS 84
## # A tibble: 65 x 9
## # Groups:   Site, Species [65]
##     Site Species              Year      meanSurvival meanUpper meanLower
##   * <dbl> <fct>                <chr>            <dbl>     <dbl>     <dbl>
##   1     2 Juniperus monosperma 2022-2023        0.985     0.997     0.968
##   2     6 Pinus edulis         2022-2023        0.719     0.852     0.575
##   3     6 Juniperus monosperma 2022-2023        0.999     1.00      0.986
##   4     8 Pinus edulis         2022-2023        0.769     0.844     0.684
##   5     8 Juniperus monosperma 2022-2023        0.939     0.965     0.899
##   6    24 Pinus edulis         2022-2023        0.784     0.846     0.725
##   7    24 Juniperus monosperma 2022-2023        0.801     0.872     0.721
##   8    28 Pinus edulis         2022-2023        0.670     0.764     0.574
##   9    28 Juniperus monosperma 2022-2023        0.956     0.980     0.912
## 10    30 Pinus edulis         2022-2023        0.863     0.921     0.789
## # i 55 more rows
## # i 3 more variables: geometry <POINT [°]>, changeInSurvival <dbl>,
## #   decline <dbl>
```

```
survivalProbs2 %>% st_drop_geometry() %>% group_by(Species) %>% summarise(mean(decline, na.rm = T)) ##
```

```
## # A tibble: 2 x 2
##   Species              `mean(decline, na.rm = T)`
```

```
##   <fct>                                        <dbl>
## 1 Pinus edulis                                 0.533
## 2 Juniperus monosperma                         0.618
```

```
b <- ggplot() +
    facet_grid(.~Species) +
    geom_spatraster(data = avgCwd, alpha = 0.7) +
    geom_spatvector(data = vect(survivalProbs2), size = 5, shape = 16, aes(color = changeInSurvival)) +
    scale_fill_gradient2(low = brewer.pal(9, "Greys")[9], mid = brewer.pal(9, "Greys")[5],
                         high = brewer.pal(9, "Greys")[1]) +
    scale_color_gradient2(
      low = '#a6611a', mid = '#f5f5f5', high = '#018571',
      midpoint = 0, guide = 'colourbar', aesthetics = 'color',
      name = expression(Delta*"Survival\nProbability"), na.value = "transparent"
    ) + theme_bw() + theme(strip.background = element_blank()) +
    theme(legend.position = "top")
```

```
## <SpatRaster> resampled to 500580 cells.
```

```
## Merge them
a+b+plot_layout(heights = c(0.7,1), ncol = 1)  + plot_annotation(tag_levels = 'a')
```

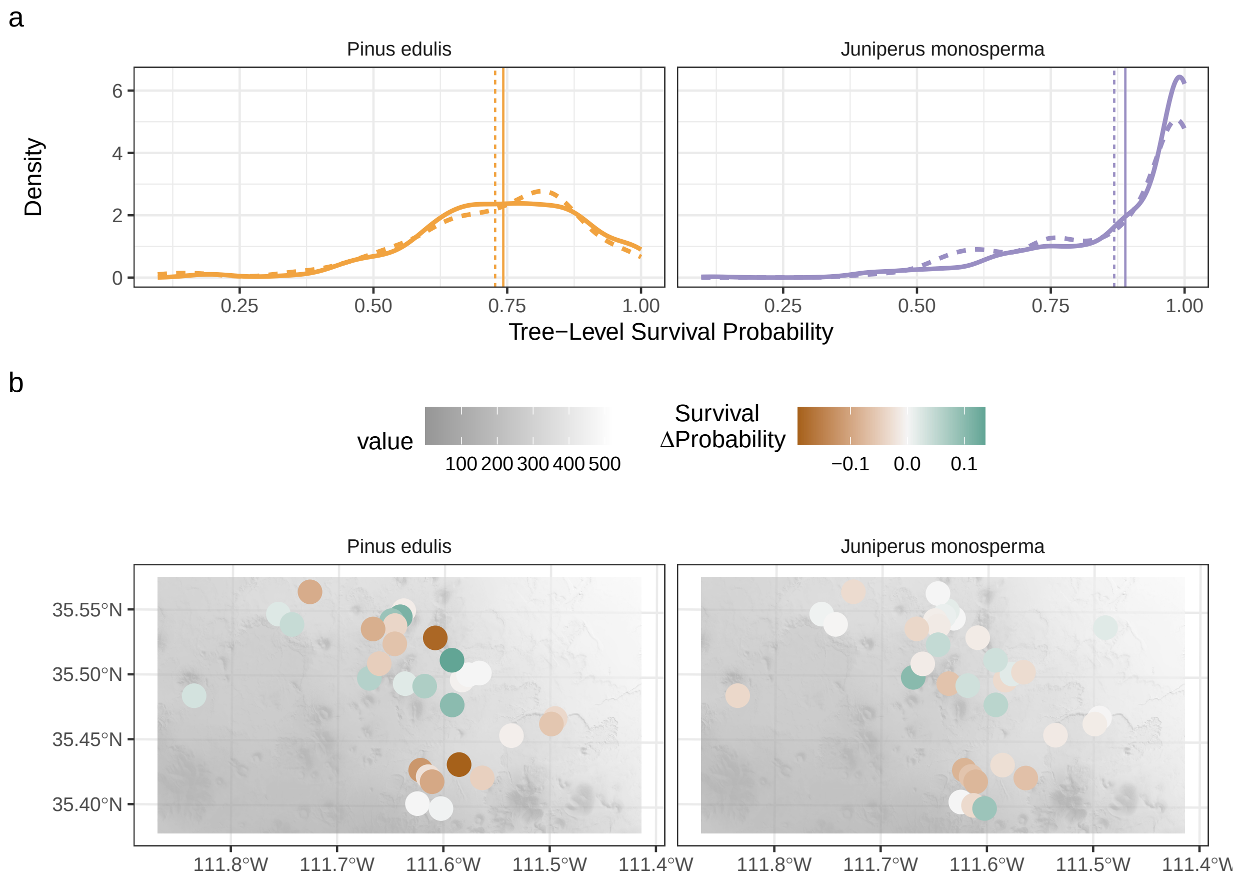

That's it! Thanks for making it to the end.

6 References

- Chaney, N. W., Wood, E. F., McBratney, A. B., Hempel, J. W., Nauman, T. W., Brungard, C. W., & Odgers, N. P. (2016). POLARIS: A 30-meter probabilistic soil series map of the contiguous United States. Geoderma, 274, 54-67.
- Flint, L. E., & Flint, A. L. (2012). Downscaling future climate scenarios to fine scales for hydrologic and ecological modeling and analysis. Ecological Processes, 1, 1-15.
- Gelman, A. (2008). Scaling regression inputs by dividing by two standard deviations. Statistics in medicine, 27(15), 2865-2873.
- Lutz, J. A., Van Wagtendonk, J. W., & Franklin, J. F. (2010). Climatic water deficit, tree species ranges, and climate change in Yosemite National Park. Journal of Biogeography, 37(5), 936-950.
- McCune, B., & Keon, D. (2002). Equations for potential annual direct incident radiation and heat load. Journal of vegetation science, 13(4), 603-606.
- Nalder, I. A., & Wein, R. W. (1998). Spatial interpolation of climatic normals: test of a new method in the Canadian boreal forest. Agricultural and forest meteorology, 92(4), 211-225.
- PRISM Climate Group, Oregon State University. (2023). http://prism.oregonstate.edu
- Redmond, M. D. (2022). CWD and AET function (Version V1. 0.3). Zenodo. https://doi.org/10.5281/zenodo.1146777.
- Redmond, M. D., Cobb, N. S., Clifford, M. J., & Barger, N. N. (2015). Woodland recovery following drought-induced tree mortality across an environmental stress gradient. Global Change Biology, 21(10), 3685-3695.
- Rodman, K. C., Veblen, T. T., Battaglia, M. A., Chambers, M. E., Fornwalt, P. J., Holden, Z. A., ... & Rother, M. T. (2020). A changing climate is snuffing out post-fire recovery in montane forests. Global Ecology and Biogeography, 29(11), 2039-2051.
- Thornton, P. E., Shrestha, R., Thornton, M., Kao, S. C., Wei, Y., & Wilson, B. E. (2021). Gridded daily weather data for North America with comprehensive uncertainty quantification. Scientific Data, 8(1), 190.
- Williams, A. P., Allen, C. D., Macalady, A. K., Griffin, D., Woodhouse, C. A., Meko, D. M., ... & McDowell, N. G. (2013). Temperature as a potent driver of regional forest drought stress and tree mortality. Nature climate change, 3(3), 292-297.
- Wilson, B. T., Lister, A. J., Riemann, R. I., & Griffith, D. M. (2013). Live tree species basal area of the contiguous United States (2000-2009).
