# Supplementary material for: Positive Drought Feedbacks Increase Tree Mortality Risk in Dry Woodlands of the US Southwest
Source: Ecol Evol. 2025 Dec 10;15(12):e72667. doi: 10.1002/ece3.72667 (PMC12696023; doi:10.1002/ece3.72667)
Supplement: Supplementary file 2 — Data S2: ece372667‐sup‐0002‐Supinfo.pdf. [file ECE3-15-e72667-s001.pdf]

Supplementary Information for:

Positive drought feedbacks increase tree mortality risk in dry woodlands of the US Southwest

Authors

Kyle C. Rodman [a,†], Kaylie J. Wilkerson [a], Andreas P. Wion [b], David W. Huffman [a], Anita J. Antoninka [c], Mariola Barrera [a], Neil S. Cobb [d], Miranda D. Redmond [e].

Author Affiliations

[a] Ecological Restoration Institute, Northern Arizona University, Flagstaff, AZ, USA.

[b] Southwest Office, Forest Stewards Guild, Santa Fe, NM, USA.

[c] School of Forestry, Northern Arizona University, Flagstaff, AZ, USA.

[d] Biodiversity Outreach Network, Flagstaff, AZ, USA.

[e] Department of Environmental Science, Policy, and Management, University of California, Berkeley, CA, USA.

[†] Corresponding author: kyle.rodman@nau.edu

Appendix S1: Methods Used to Describe Arbuscular Mycorrhizal Colonization (Total AM), and Relationships Between Total AM and Other Biophysical Variables

Field Sampling and Laboratory Processing Methods

To quantify root colonization by arbuscular mycorrhizal (AM) fungi, mutualists of one-seed juniper (*Juniperus monosperma*) and many shrub and herbaceous species at our sites (Haskins and Gehring, 2004), we used standard methods to collect soil cores, methods of (Johnson, O'Dell and Bledsoe, 1999) for sample processing, and the visual intersection method of McGonigle *et al.* (1990) to quantify root colonization. This section describes these methods, and how they were used with our soil samples collected in 2023.

We identified sampling sites using preliminary results of data collection from 2022 (i.e., sites surveyed in 2023 were not included), and tree records from these same sites in 2014 (Redmond *et al.*, 2015). Mycorrhizae can be most effectively sampled from large, live trees. Thus, we identified plots with large (i.e., > 25 cm diameter at root collar [DRC]) juniper trees that were alive in 2022 surveys (n = 87). Because we were interested in the effects of mycorrhizae on seedling survival, we then identified plots with smaller juniper (i.e., < 5 cm DRC *and* [> 0.5 cm DRC *or* > 5 cm in height]) that were alive in 2014, irrespective of their final status in 2022 measurements. This resulted in 34 candidate plots for sampling. From this subset of plots meeting our criteria, we collected soil cores from 26 plots on 8 sites. These plots were randomly selected from the larger list, and the remaining plots were omitted due to limited time available for laboratory processing. At each field site, we used soil core extractors (11.43-cm diameter x 10-cm depth), to collect soil samples for mycorrhizal analysis. For sampling, we first selected the large (i.e., > 25 cm DRC), live juniper tree that was closest to plot center. We then collected a soil core located at the north dripline of this tree. Soil cores were inserted 10 cm deep into the

soil with a sledgehammer, then sampled soil was immediately transferred to a Ziploc bag inside a plastic cooler for storage in the field. Each evening, we returned soil samples to the laboratory for storage in a freezer at ca. -20 °C until we could complete laboratory processing.

In the laboratory, we separated roots from other materials using a series of sieves. First, we washed these samples over a 5000 µm sieve by introducing a slow flow of water. This process was repeated with a 2000 µm sieve followed with a 1000 µm sieve, until roots were clear of any dissolved organic matter and fine soil particles. We then stored root samples in a freezer to limit respiration and decomposition until the following stages of sample processing could be completed. We added a random subsample of roots from each sample to small, plastic cassettes, which we placed in a beaker containing 2.5% potassium hydroxide (KOH) and covered with aluminum foil. We warmed the samples and KOH solution in a water bath (70-90 °C) for an hour inside a fume hood. This procedure cleared the cytoplasm out of the cortical cells, so we extended times as needed for thicker roots. After clearing, we emptied the KOH solution, and rinsed cassettes with tap water. If roots remained pigmented, we also soaked them in an alkaline hydrogen peroxide (H_2O_2) solution. Next, we submerged all root samples in a 1% hydrochloric acid (HCL) solution for one hour. Finally, we removed samples and added them to an ink-vinegar solution (staining roots to accentuate mycorrhizal structures), which we kept in a warm water bath for one hour. We stored these stained roots in distilled water in the refrigerator for two weeks, or in a sealed dry container in the freezer for a longer period, as needed.

We recorded the colonization of AM fungi in each sample using a compound microscope at 200x magnification. To do so, we arranged stained roots on a microscope slide with coverslips. A hairline graticule was inserted in the eyepiece of a compound microscope to act as a line of intersection with the roots. We then recorded the presence and category of AM and other fungal

structures at 150 grid intersections, evenly distributed throughout the same. We used six categories of intersections: (1) no fungal structures, (2) non-AM hyphae, (3) AM hyphae, (4) AM vesicles, (5) AM arbuscules, and (6) both arbuscules and vesicles. We used a physical counting device to record the number of intersections, and a single analyst (K. Wilkerson) completed all scoring to prevent observer-to-observer variation. We calculated total AM colonization as the sum of all points with evidence of AM hyphae, vesicles, or arbuscules.

Comparing Total AM with Live Juniper BA, Soil OM, and Average CWD

Total AM was only characterized for a subset of plots, as described above, thus we performed three exploratory analyses to better understand how mycorrhizal colonization might vary according to variables that were described throughout the rest of study area. These analyses aided in interpreting results of Models #1 and #2 in the main text, the potential mechanisms that soil communities might play in these models (e.g., relationships between tree survival and environmental variation/stand structure), and how soil communities might respond to future tree mortality. Specifically, we fit three individual generalized linear mixed models, each of which included Total AM as a plot-scale response variable. In each model, we used a binomial error structure with a complementary log-log link, where our plot-scale response was defined as the number of grid intersections that contained evidence of AM hyphae, vesicles, or arbuscules (# successes), out of the total 150 sample points (# trials). We used a random intercept term of site to account for spatial dependence among plots within each site. Because limited sample sizes ($n = 26$ plots) precluded multivariate analyses, each model included a single fixed effect. As covariates in individual models, we tested (1) average climatic water deficit (CWD) of a plot between 1991 and 2020, (2) the total basal area (BA) of all live one-seed juniper present on a plot in 2014, and (3) soil organic matter (OM) of a site, based on soil samples collected in 2014.

We assumed that Total AM might vary with Average CWD, because it describes typical temperature and moisture availability at a site. We also hypothesized that Total AM would be higher in locations with more abundant juniper due to greater root colonization from adjacent trees. Finally, we assumed that Soil OM might influence Total AM, because many soil characteristics are known to influence mycorrhizal communities (Gehring and Whitham, 1994). We scaled and centered each variable following methods of Gelman (2008) prior to model fitting, to permit comparisons of effect sizes and increase model stability. We fit each model using the *glmmTMB* package (Brooks *et al.*, 2017) in R software v. 4.4.0 (R Core Team, 2024), and interpreted statistical significance using $\alpha = 0.05$ (Table S1.1).

Table S1.1: Results of fitted generalized linear mixed models used to predict plot-level colonization of arbuscular mycorrhizae (Total AM) in long-term monitoring plots in northern Arizona, USA. Due to sample size limitations (n = 26 plots), we fit three separate models, each with a single fixed effect term, to explain potential drivers of variation in Total AM fungal colonization. Covariates were standardized in the manner of Gelman (2008), so coefficient estimates are on a common scale, rather than the original scale of each predictor. Random intercept terms (1|Site) and response variables (here, the proportion of sampled root tips that were colonized by AM fungi) were held constant across models.

**Model Formula**	**Covariate**	**Coefficient Estimate**	**Std. Error**	***Z***	**Pr(>|*Z*|)**	**Intercept**	**Random Intercept Variance (σ^2 of Site)**
Total AM ~ Average CWD + (1|Site)	Average CWD (1991-2020)	-0.891	0.498	-1.790	0.073	-3.455	0.457
Total AM ~ Juniper BA + (1|Site)	Live Juniper BA (2014)	0.475	0.152	3.122	0.002	-3.599	1.248
Total AM ~ Soil OM + (1|Site)	Soil OM (2014)	1.334	0.563	2.372	0.018	-3.389	0.523

Appendix S2: Description of Variables Used to Predict Tree Mortality

Using field inventories, we summarized metrics that described individual-environment interactions or linked disturbance effects between short-interval drought events (Table 1 in the main text). We used field-derived measurements of diameter at root collar (DRC) to describe tree size. As trees in these systems grow slowly and measurement error of DRC is likely to exceed any changes in tree size between plot remeasurements, we assumed that trees remained a constant size throughout our study period based on the most reliable measurement. Specifically, we used DRC measurements from either 1998-2001 or 2014, as used in Redmond *et al.* (2015), or 2022-2023 measurements for newly established trees. Next, we summarized live basal area (Live BA) as the cross-sectional area (at root collar) of all live trees in a plot in 2014. We summarized Proportion BA Loss as the proportion of Live BA present in initial plot measurements (i.e., 1998-2001) that was dead in 2014. We summarized individual tree health using Live Crown, an ordinal categorical term describing the percentage of a tree crown with live foliage (i.e., < 15%, 16-50%, 51-90%, > 90%) in 2014. We summarized soil organic matter (Soil OM) using soil samples collected at each site in 2014. To characterize microclimatic modification and availability of nurse structures, we summarized Wood Cover using line-point intercept transects collected in 2014 (Redmond *et al.*, 2015). For a targeted subset of plots, we quantified arbuscular mycorrhizal colonization (Total AM) using samples collected in 2023, as the percentage of root intersection points showing evidence of AM (Appendix S1).

To describe variation in moisture stress experienced by plants, we developed 10-m estimates of annual climatic water deficit (Average CWD; mm year $^{-1}$) throughout the study area (Fig. S2.1). Average CWD combines temperature, precipitation, aspect-driven solar heating, and soil texture into a single index that represents the average moisture stress experienced by plants

at a given location (Stephenson, 1998). To calculate CWD, we obtained maps of monthly maximum temperature, minimum temperature, and total precipitation from Daymet (1-km spatial resolution; Thornton *et al.*, 2021). We spatially downscaled these data to a 30-m spatial resolution using Gradient and Inverse Distance-Squared (GIDS) interpolation (Nalder and Wein, 1998; Rodman *et al.*, 2020) with a digital elevation model (DEM) as ancillary data (USGS, 2021). We calculated heat load index using a 10-m DEM following Eq. 3 in McCune & Keon (2002), and soil available capacity in the top 200 cm of the soil profile as the product of fractional water availability and soil depth layers from POLARIS (30-m spatial resolution; Chaney et al., 2016). We could not use locally collected soil texture information for this purpose because they captured only the top 10 cm of the soil profile, though GIS-derived soil information was significantly predictive of field-derived soils at the site level (Appendix S2; $p < 0.01$). Using these inputs, we ran a modified Thornthwaite water balance model at a monthly time step from October 1989 to September 2020 to calculate CWD (Lutz, van Wagtendonk and Franklin, 2010; Redmond, 2022). We summed monthly totals of CWD by water year (Oct 1st of the prior year to Sep 30th of the focal year) and calculated the mean annual value for the 1991 to 2020 normal period. We used the 1990 water year as a burn-in period to initiate soil moisture and snowpack parameters in 1991.

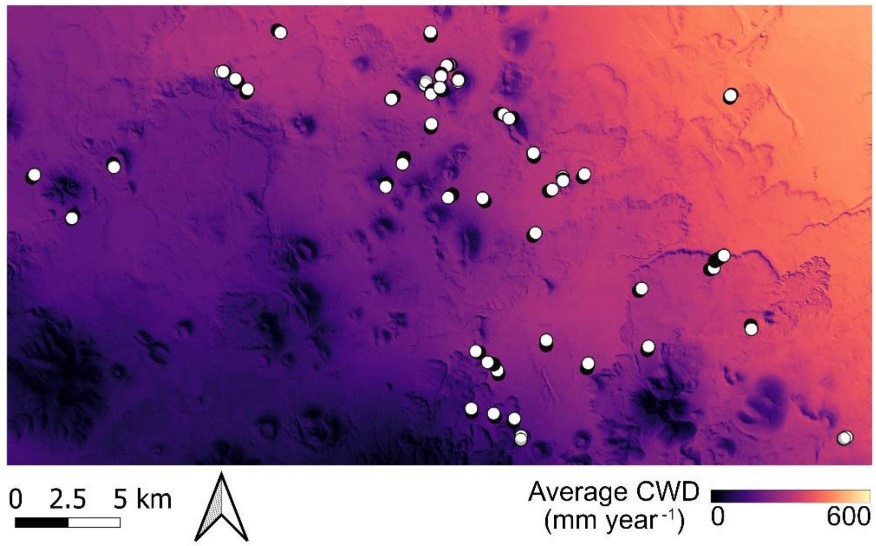

Figure S2.1: Average climatic water deficit (CWD) from 1991 to 2020 across the study area in pinyon (*Pinus edulis*) and juniper (*Juniperus monosperma*) woodlands of northern Arizona, USA. CWD was developed using a monthly water balance model which incorporated spatially downscaled climate data, topography, and soil available water capacity. Locations of permanent monitoring plots are shown with white circles.

Appendix S3: Validation of GIS-Derived Soils Maps Using Field Data

Digitized soils maps (e.g., the Natural Resource Conservation Service, Soil Survey Geographic Database; NRCS SSURGO) are increasingly utilized for ecological research because soils have a strong influence on plant communities and hydrological dynamics. Soil mapping efforts have improved in recent years, with the development of imputation methods that fill unmapped locations and smooth mapping irregularities across administrative boundaries (e.g., county lines, state lines), however, such information is often utilized without local field validation. Here, we utilized datasets from POLARIS (Chaney *et al.*, 2016) as input into monthly water balance models. POLARIS is a gridded soils data product, spanning the United States, that includes information about soil texture, depth, and other important properties at a 30-m spatial resolution. We collected soil cores (2.5 cm diameter x 10 cm depth) from each field site in 2014, and characterized fractional water capacity (i.e., m^3 of water storage/m^3 of soil volume) following equations in Table 1 of Saxton & Rawls (2006). Specifically, these equations incorporate sand, silt, and clay fractions, as well as gravel and organic matter content to estimate plant-available soil water.

At the site-level, we compared these field-derived soil samples (further described in Redmond et al., 2015) to GIS-derived fractional water capacity in our study area. In POLARIS, fractional water capacity is provided as separate gridded products by soil depth (i.e., 0-5 cm, 5-15 cm, 15-30 cm, 30-60 cm, 60-100 cm, 100-200 cm). Thus, we compared average fractional water capacity from 0 to 10 cm in field samples with the same variable from 0-15 cm in POLARIS (using a depth-weighted average of the 0-5 and 5-15 cm datasets). We extracted POLARIS data at the location of each field plot and calculated the mean across plots to determine a site-level average. We then compared field-derived soil samples with estimates of

the same metric from POLARIS using ordinary least squares regression (Fig. S3.1). While GIS-derived soils data were not a perfect predictor of field-derived values, the model indicated a significant relationship ($p < 0.01$), and model predictions closely aligned with the 1:1 line (i.e., intercept near 0 and a slope near 1). We utilized POLARIS data in monthly water balance models because they were available throughout the entire rooting zone (i.e., 0 - 200 cm) and the extent of the study area, whereas field-inventoried soils data were only available for the top of the soil profile in specific locations.

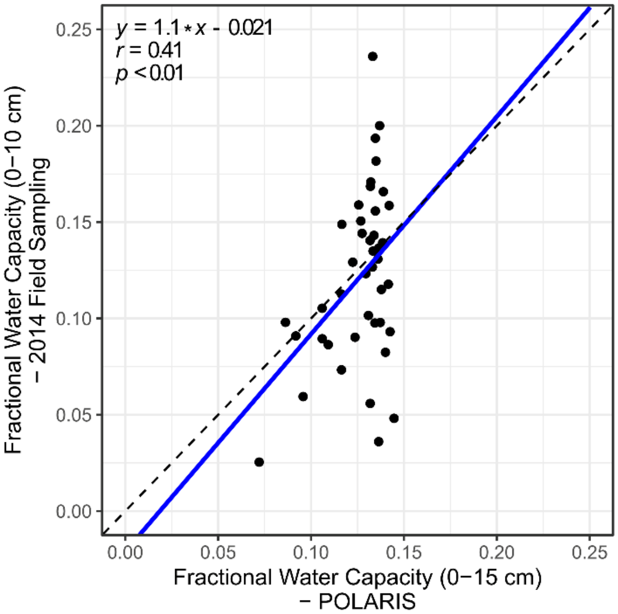

Figure S3.1: Validation of GIS-derived soil fractional water capacity (m^3 of plant-available water storage/m^3 of soil) (x-axis; Chaney et al., 2016), using field-derived soil texture information collected in 2014 from the top 10 cm of the soil profile (Redmond *et al.*, 2015). Parameters from ordinary least squares regression are shown in the top-left, indicating that GIS-derived soils are predictive of field values ($p < 0.01$), with model predictions (solid blue line) closely aligning with the 1:1 line (dashed black line). We used GIS-derived available water capacity of soils to parameterize monthly water balance models in the study area – locally collected field data could not be used for this because they only characterized the top of the soil profile, rather than the entire rooting zone.

Appendix S4: Supplementary Figures and Tables Showing Changes in PJ Woodlands From 1998 to 2023

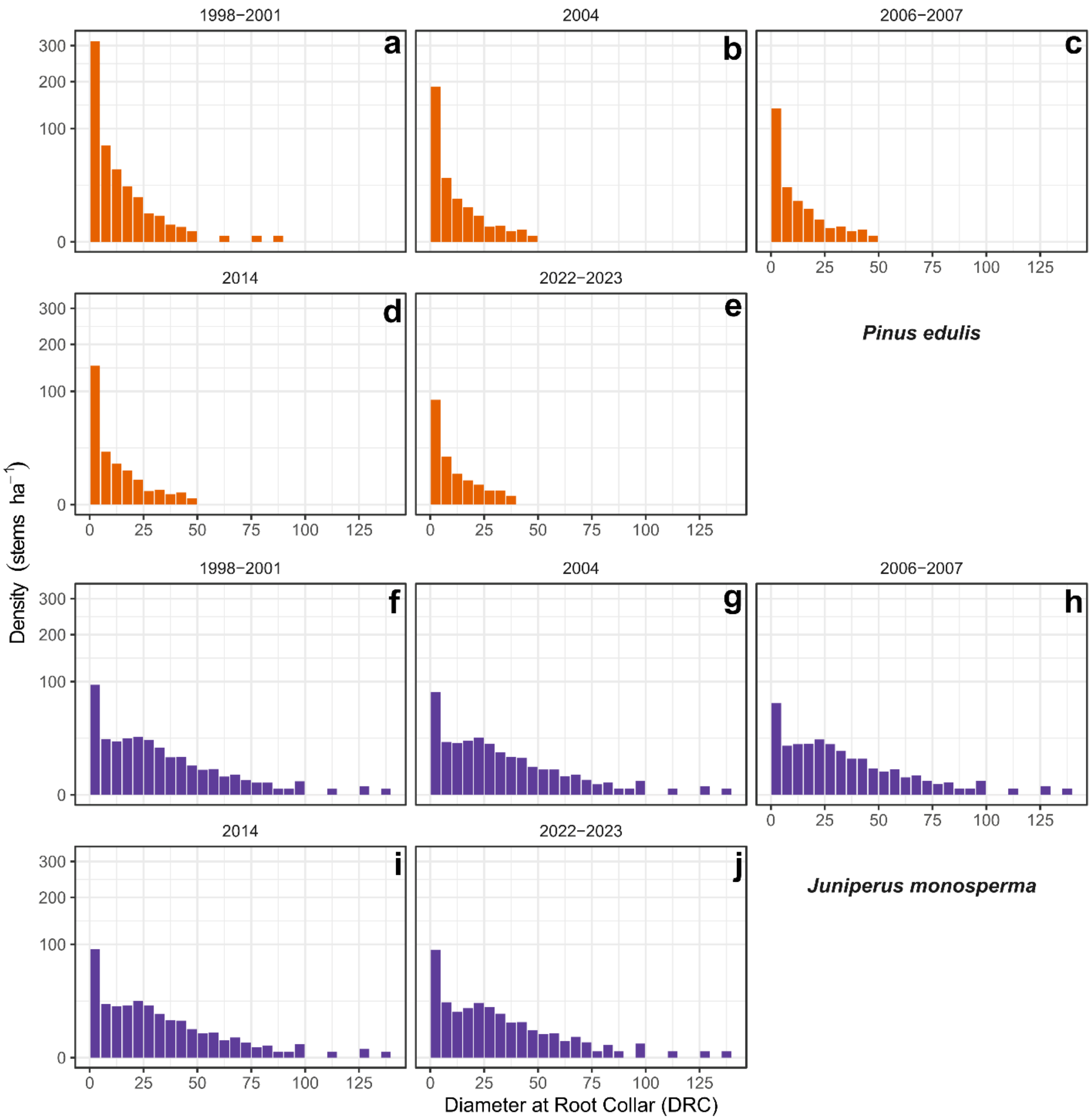

Figure S4.1: Changes in live tree size distributions from 1998 to 2023 in two-needle pinyon (*Pinus edulis*) and one-seed juniper (*Juniperus monosperma*) communities of northern Arizona, USA.

Table S4.1: Mean (standard deviation across sites) of the density, basal area, and relative dominance (ecological importance value [EIV]; Curtis & McIntosh, 1951) for live trees of two-needle pinyon (*Pinus edulis*) and one-seed juniper (*Juniperus monosperma*) at long-term monitoring plots in northern Arizona, USA. Numbers are restricted to the 32 monitoring sites that did not experience severe fire or intensive forest management during the study period. A visual depiction of these metrics is provided in Fig. 3 of the main text.

**Survey Interval**	**Density (stems ha^{-1})**		**Basal Area (m^2 ha^{-1})**		**Relative Dominance (EIV)**	
	P. edulis	*J. monosperma*	*P. edulis*	*J. monosperma*	*P. edulis*	*J. monosperma*
1998-2001	378.6 (50.7)	232.9 (29.0)	3.2 (0.7)	15.6 (2.3)	71.0 (6.3)	110.9 (7.7)
2004	174.9 (28.7)	211.6 (28.5)	0.8 (0.2)	14.9 (2.1)	43.9 (5.4)	136.1 (8.7)
2006-2007	153.0 (26.7)	199.0 (25.2)	0.7 (0.2)	14.8 (2.1)	41.4 (5.4)	139.4 (9.0)
2014	135.0 (25.3)	214.7 (28.1)	0.7 (0.2)	14.4 (2.0)	36.6 (5.2)	144.5 (8.8.)
2022-2023	99.6 (19.0)	211.5 (19.0)	0.6 (0.2)	14.7 (2.0)	29.7 (3.4)	151.0 (9.0)

Table S4.2: All subsets model selection results – based on the sample-size corrected variant of the Akaike information criterion (AICc) – of generalized linear mixed models used to predict two-needle pinyon (*Pinus edulis*) survival at long-term monitoring plots in northern Arizona, USA. Rows represent individual models, where "NA" indicates that a term (shown in columns) was not included in a specific model. Cell values represent the coefficient estimate for a given term and model. Covariates were standardized in the manner of Gelman (2008), so coefficient estimates are on a common scale, rather than the original scale of each predictor. Interactions between two variables are separated using a ":" symbol. For brevity, only models with partial support (ΔAICc < 4) are shown here. The top model, described in the main text, is ***bolded and italicized***. CWD: Climatic Water Deficit; DRC: Diameter at Root Collar; BA: Basal Area; OM: Organic Matter; DF: Degrees of Freedom.

CWD	DRC	Live BA	Live Crown	Prop. BA Loss	Soil OM	Wood Cover	CWD: DRC	DRC: Live BA	Live BA: Prop. BA Loss	DF	logLik	AICc	ΔAICc
0.03	***0.64***	***NA***	***+***	***-0.29***	***NA***	***NA***	***-1.08***	***NA***	***NA***	***10***	***-184.08***	***388.79***	***0.00***
0.02	0.68	NA	+	NA	NA	NA	-1.08	NA	NA	9	-185.18	388.87	0.08
-0.04	0.61	-0.30	+	-0.40	NA	NA	-1.08	NA	NA	11	-183.09	388.92	0.14
-0.01	0.68	-0.16	+	NA	NA	NA	-1.08	NA	NA	10	-184.91	390.45	1.66
0.01	0.68	NA	+	NA	NA	-0.13	-1.09	NA	NA	10	-185.00	390.62	1.83
-0.02	0.60	-0.18	+	-0.18	NA	NA	-1.07	NA	0.47	12	-182.94	390.76	1.98
0.04	0.63	NA	+	-0.29	0.04	NA	-1.08	NA	NA	11	-184.07	390.89	2.10
0.03	0.64	NA	+	-0.28	NA	-0.03	-1.08	NA	NA	11	-184.08	390.90	2.11
0.03	0.68	NA	+	NA	0.03	NA	-1.08	NA	NA	10	-185.18	390.97	2.18
-0.04	0.62	-0.31	+	-0.39	NA	NA	-1.12		NA	12	-183.05	390.99	2.20
-0.03	0.61	-0.30	+	-0.40	0.05	NA	-1.07	NA	NA	12	-183.07	391.02	2.23
-0.04	0.61	-0.31	+	-0.41	NA	0.03	-1.08	NA	NA	12	-183.08	391.04	2.25
NA	0.54	NA	+	NA	NA	NA	NA	NA	NA	7	-188.62	391.56	2.77
NA	0.50	NA	+	-0.27	NA	NA	NA	NA	NA	8	-187.65	391.69	2.91
NA	0.48	-0.27	+	-0.37	NA	NA	NA	NA	NA	9	-186.78	392.06	3.28
-0.02	0.68	-0.15	+	NA	NA	-0.12	-1.09	NA	NA	11	-184.74	392.23	3.44
-0.01	0.69	-0.17	+	NA	NA	NA	-1.14		NA	11	-184.84	392.42	3.63
0.00	0.68	-0.16	+	NA	0.03	NA	-1.08	NA	NA	11	-184.91	392.56	3.77
0.02	0.68	NA	+	NA	0.03	-0.13	-1.09	NA	NA	11	-184.99	392.73	3.94

Table S4.3: Fixed effects from the top generalized linear mixed model used to predict two-needle pinyon (*Pinus edulis*) survival at long-term monitoring plots in northern Arizona, USA. Covariates were standardized in the manner of Gelman (2008), so coefficient estimates are on a common scale, rather than the original scale of each predictor. BA: Basal Area; CWD: Climatic Water Deficit; DRC: Diameter at Root Collar. Live crown, an ordinal categorical variable, was modeled using orthogonal polynomial contrasts, which include linear, quadratic, and cubic components. Interactions between two variables are separated using a ":" symbol.

Covariate	Coefficient Estimate	Std. Error	*Z*
Intercept	-0.220	0.290	-0.760
Average CWD	0.091	0.249	0.364
DRC	0.623	0.229	2.722
Live Crown - Linear	1.593	0.709	2.247
Live Crown - Quadratic	-0.559	0.545	-1.026
Live Crown - Cubic	0.300	0.308	0.975
Prop. BA Loss	-0.304	0.195	-1.554
CWD:DRC	-1.131	0.457	-2.477

Table S4.4: Estimated variance components from random effects of the top generalized linear mixed model used to predict two-needle pinyon (*Pinus edulis*) survival at long-term monitoring plots in northern Arizona, USA. Random intercept terms were modeled hierarchically such that sites were centered around an overall intercept term, and plots were centered around the site in which they were located.

Group	Variance (σ^2)
Site	0.176
Plot:Site	0.133

Table S4.5: All subsets model selection results – based on the sample-size corrected variant of the Akaike information criterion (AICc) – of generalized linear mixed models used to predict one-seed juniper (*Juniperus monosperma*) survival at long-term monitoring plots in northern Arizona, USA. Cell values represent the coefficient estimate for a given term and model. Covariates were standardized in the manner of Gelman (2008), so coefficient estimates are on a common scale, rather than the original scale of each predictor. Interactions between two variables are separated using a ":" symbol. For brevity, only models with partial support (ΔAICc < 4) are shown here. The top model, described in the main text, is ***bolded and italicized***. CWD: Climatic Water Deficit; DRC: Diameter at Root Collar; BA: Basal Area; OM: Organic Matter; DF: Degrees of Freedom.

CWD	DRC	Live BA	Live Crown	Prop. BA Loss	Soil OM	Wood Cover	CWD: DRC	DRC: Live BA	Live BA: Prop. BA Loss	DF	logLik	AICc	ΔAICc
0.30	***2.25***	***0.06***	***+***	***NA***	***0.50***	***NA***	***1.11***	***-1.09***	***NA***	***12***	***-245.81***	***516.04***	***0.00***
0.22	2.23	-0.07	+	-0.29	0.50	NA	1.10	-1.04	NA	13	-244.95	516.39	0.34
0.19	2.19	0.05	+	NA	NA	NA	1.14	-1.07	NA	11	-247.45	517.26	1.22
0.34	2.24	0.06	+	NA	0.49	0.15	1.08	-1.07	NA	13	-245.59	517.67	1.63
0.27	2.21	-0.08	+	-0.32	0.48	0.19	1.07	-1.01	NA	14	-244.57	517.72	1.68
0.11	2.16	-0.08	+	-0.27	NA	NA	1.14	-1.03	NA	12	-246.69	517.80	1.76
0.19	2.24	-0.20	+	-0.47	0.50	NA	1.08	-1.07	-0.47	14	-244.84	518.24	2.20
0.24	2.18	0.06	+	NA	NA	0.19	1.11	-1.05	NA	12	-247.09	518.60	2.56
0.18	2.15	-0.09	+	-0.31	NA	0.24	1.09	-1.00	NA	13	-246.15	518.79	2.74
0.23	2.23	-0.28	+	-0.60	0.49	0.23	1.02	-1.05	-0.70	15	-244.33	519.31	3.27

Table S4.6: Fixed effects from the top generalized linear mixed model used to predict one-seed juniper (*Juniperus monosperma*) survival at long-term forest monitoring plots in northern Arizona, USA. Covariates were standardized in the manner of Gelman (2008), so coefficient estimates are on a common scale, rather than the original scale of each predictor. CWD: Climatic Water Deficit; DRC: Diameter at Root Collar. Live crown, an ordinal categorical variable, was modeled using orthogonal polynomial contrasts, which include linear, quadratic, and cubic components. Interactions between two variables are separated using a ":" symbol.

Covariate	Coefficient Estimate	Std. Error	*Z*
Intercept	1.077	0.278	3.871
Average CWD	0.328	0.284	1.155
DRC	2.310	0.403	5.733
Live BA	0.052	0.269	0.194
Live Crown - Linear	0.620	0.542	1.145
Live Crown - Quadratic	0.447	0.429	1.041
Live Crown - Cubic	-0.247	0.287	-0.858
Soil OM	1.156	0.500	2.315
Average CWD:DRC	0.500	0.298	1.676
DRC:Live BA	-1.121	0.327	-3.428

Table S4.7: Estimated variance components from random effects of the top generalized linear mixed model used to predict one-seed juniper (*Juniperus monosperma*) survival in long-term permanent plots in northern Arizona, USA. Random intercept terms were modeled hierarchically such that sites were centered around an overall intercept term, and plots were centered around the site in which they were located.

Group	Variance (σ^2)
Site	0.145
Plot:Site	0.626

Table S4.8: All subsets model selection results – based on the sample-size corrected variant of the Akaike information criterion (AICc) – of generalized linear mixed models used to predict survival of smaller (< 5 cm DRC) one-seed juniper (*Juniperus monosperma*) in long-term monitoring plots in northern Arizona, USA. This analysis was restricted to plots with field-derived sampling of arbuscular mycorrhizal (AM) colonization. Cell values represent the coefficient estimate for a given term and model. Covariates were standardized in the manner of Gelman (2008), so coefficient estimates are on a common scale, rather than the original scale of each predictor. Interactions between two variables are separated using a ":" symbol. The top model, described in the main text, is ***bolded and italicized***. DRC: Diameter at Root Collar; DF: Degrees of Freedom.

DRC	Total AM	DRC: Total AM	DF	logLik	AICc	ΔAICc
1.986	***1.682***	***NA***	***5***	***-41.361***	***93.523***	***0.000***
2.395	2.133	1.565	6	-40.900	94.951	1.430
1.592	NA	NA	4	-45.019	98.564	5.040
NA	0.843	NA	4	-48.853	106.233	12.710
NA	NA	NA	3	-50.600	107.489	13.970

Table S4.9: Fixed effects from the top generalized linear mixed model used to predict survival of smaller (< 5 cm DRC) one-seed juniper (*Juniperus monosperma*) in long-term monitoring plots in northern Arizona, USA. Covariates were standardized in the manner of Gelman (2008), so coefficient estimates are on a common scale, rather than the original scale of each predictor. This analysis was restricted to plots with field-derived sampling of arbuscular mycorrhizal (AM) colonization. DRC: Diameter at Root Collar.

Covariate	Coefficient Estimate	Std. Error	*Z*
Intercept	0.222	0.281	0.789
DRC	1.986	0.715	2.779
Total AM	1.682	0.756	2.225

Table S4.10: Estimated variance components from random effects of the top generalized linear mixed model used to predict survival of smaller (< 5 cm DRC) one-seed juniper (*Juniperus monosperma*) in long-term monitoring plots in northern Arizona, USA. This analysis was restricted to plots with field-derived sampling of arbuscular mycorrhizal (AM) colonization. Random intercept terms were modeled hierarchically such that sites were centered around an overall intercept term, and plots were centered around the site in which they were located.

Group	Variance (σ^2)
Site	< 0.001
Plot:Site	0.644

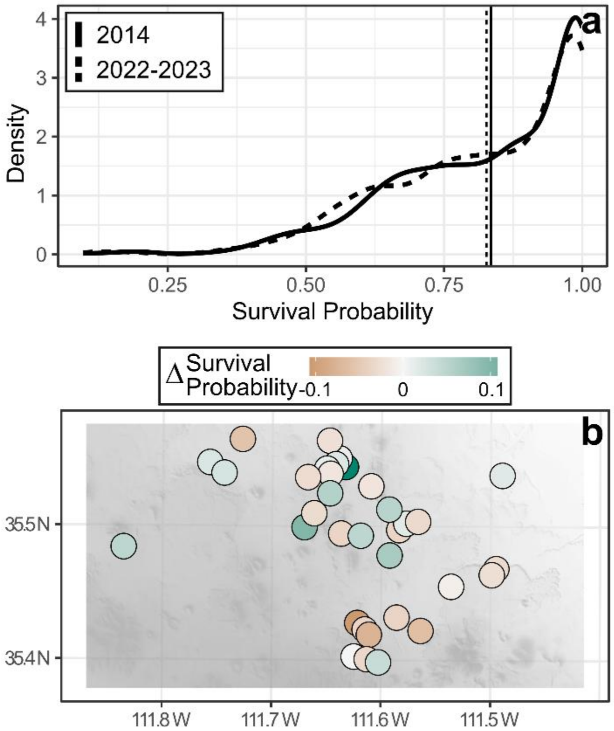

Figure S4.2: Changes in survival probability between 2014 and 2023 in pinyon (*Pinus edulis*) and juniper (*Juniperus monosperma*) woodlands of northern Arizona, USA. The distribution of tree-level survival probabilities in (a) shows predictions of tree survival for all live individuals present in 2014 (solid lines) or 2022-2023 (dashed lines). Mean survival probabilities in (a) are shown with vertical lines. Point colors in (b) show how changes in tree density, size structure, species composition, and crown dieback have altered mean survival probabilities of all pinyon and juniper trees within individual sites.

References

Brooks, M.E. *et al.* (2017) “glmmTMB balances speed and flexibility among packages for zero-inflated generalized linear mixed modeling,” *The R Journal*, 9(2), pp. 378–400. Available at: https://doi.org/10.32614/RJ-2017-066.

Chaney, N.W. *et al.* (2016) “POLARIS: A 30-meter probabilistic soil series map of the contiguous United States,” *Geoderma*, 274, pp. 54–67. Available at: https://doi.org/10.1016/j.geoderma.2016.03.025

Curtis, J.T. and McIntosh, R.P. (1951) “An upland forest continuum in the prairie-forest border region of Wisconsin,” *Ecology*, 32(3), pp. 476–496. Available at: https://doi.org/10.2307/1931725.

Gehring, C.A. and Whitham, T.G. (1994) “Comparisons of ectomycorrhizae on pinyon pines (*Pinus edulis*; Pinaceae) across extremes of soil type and herbivory,” *American Journal of Botany*, 81(12), pp. 1509–1516. Available at: https://doi.org/10.1002/j.1537-2197.1994.tb11461.x.

Gelman, A. (2008) “Scaling regression inputs by dividing by two standard deviations,” *Statistics in Medicine*, 27, pp. 2865–2873. Available at: https://doi.org/10.1002/sim.3107.

Haskins, K.E. and Gehring, C.A. (2004) “Interactions with juniper alter pinyon pine ectomycorrhizal fungal communities,” *Ecology*, 85(10), pp. 2687–2692. Available at: https://doi.org/10.1890/04-0306.

Johnson, N.C., O’Dell, T.E. and Bledsoe, C.S. (1999) “Methods for ecological studies of mycorrhizae,” in *Standard soil methods for long-term ecological research*. New York, NY: Oxford University Press, pp. 378–412.

Lutz, J.A., van Wagtendonk, J.W. and Franklin, J.F. (2010) “Climatic water deficit, tree species ranges, and climate change in Yosemite National Park,” *Journal of Biogeography*, 37(5), pp. 936–950. Available at: https://doi.org/10.1111/j.1365-2699.2009.02268.x.

McCune, B. and Keon, D. (2002) “Equations for potential annual direct incident radiation and heat load,” *Journal of Vegetation Science*, 13, pp. 603–606. Available at: https://doi.org/10.1111/j.1654-1103.2002.tb02087.x.

McGonigle, T.P. *et al.* (1990) “A new method which gives an objective measure of colonization of roots by vesicular—arbuscular mycorrhizal fungi,” *New Phytologist*, 115(3), pp. 495–501. Available at: https://doi.org/10.1111/j.1469-8137.1990.tb00476.x.

Nalder, I.A. and Wein, R.W. (1998) “Spatial interpolation of climatic normals: test of a new method in the canadian Boreal Forest,” *Agricultural and Forest Meteorology*, 92, pp. 211–225. Available at: https://doi.org/10.1016/S0168-1923(98)00102-6.

R Core Team (2024) “R: a language and environment for statistical computing.” Vienna, Austria: R Foundation for Statistical Computing. Available at: https://www.r-project.org/.

Redmond, M.D. *et al.* (2015) “Woodland recovery following drought-induced tree mortality across an environmental stress gradient,” *Global Change Biology*, 21, pp. 3685–3695. Available at: https://doi.org/10.1111/gcb.12976.

Redmond, M.D. (2022) “CWD and AET function.” Available at: https://doi.org/10.5281/zenodo.6416352.

Rodman, K.C. *et al.* (2020) “A changing climate is snuffing out post-fire recovery in montane forests,” *Global Ecology and Biogeography*, 29(11), pp. 2039–2051. Available at: https://doi.org/10.1111/GEB.13174.

Saxton, K.E. and Rawls, W.J. (2006) “Soil water characteristic estimates by texture and organic matter for hydrologic solutions,” *Soil Science Society of America Journal*, 70(5), pp. 1569–1578. Available at: https://doi.org/10.2136/sssaj2005.0117.

Stephenson, N.L. (1998) “Actual evapotranspiration and deficit: biologically meaningful correlates of vegetation distribution across spatial scales,” *Journal of Biogeography*, 25(5), pp. 855–870. Available at: https://doi.org/10.1046/j.1365-2699.1998.00233.x.

Thornton, P.E. *et al.* (2021) “Gridded daily weather data for North America with comprehensive uncertainty quantification,” *Scientific Data*, 8(1), pp. 1–17. Available at: https://doi.org/10.1038/s41597-021-00973-0.

United States Geological Survey (2021) “USGS 3D Elevation Program Digital Elevation Model.” Available at: https://www.usgs.gov/3d-elevation-program.
